# Chemotaxonomic Profiling of Canadian *Alternaria* Populations Using High-Resolution Mass Spectrometry

**DOI:** 10.3390/metabo10060238

**Published:** 2020-06-09

**Authors:** Megan J. Kelman, Justin B. Renaud, Keith A. Seifert, Jonathan Mack, Ken K.-C. Yeung, Mark W. Sumarah

**Affiliations:** 1 London Research and Development Center, Agriculture and Agri-Food Canada, London, ON N5V 4T3, Canada; megan.kelman@canada.ca (M.J.K.); justin.renaud@canada.ca (J.B.R.); 2 Department of Chemistry, University of Western Ontario, London, ON N6A 5B7, Canada; kyeung@uwo.ca; 3 Ottawa Research and Development Centre, Agriculture and Agri-Food Canada, Ottawa, ON K1A 0C6, Canada; Keith.seifert@carleton.ca (K.A.S.); jonathan.mack2@canada.ca (J.M.); 4 Department of Biochemistry, University of Western Ontario, London, ON N6A 5C1, Canada

**Keywords:** *Alternaria*, high-resolution mass spectrometry, metabolomics, chemotaxonomy

## Abstract

*Alternaria* spp. occur as plant pathogens worldwide under field and storage conditions. They lead to food spoilage and also produce several classes of secondary metabolites that contaminate the food production chain. From a food safety perspective, the major challenge of assessing the risk of *Alternaria* contamination is the lack of a clear consensus on their species-level taxonomy. Furthermore, there are currently no reliable DNA sequencing methods to allow for differentiation of the toxigenic potential of these fungi. Our objective was to determine which species of *Alternaria* exist in Canada, and to describe the compounds they make. To address these issues, we performed metabolomic profiling using liquid chromatography high-resolution mass spectrometry (LC-HRMS) on 128 Canadian strains of *Alternaria* to determine their chemotaxonomy. The *Alternaria* strains were analyzed using principal component analysis (PCA) and unbiased *k*-means clustering to identify metabolites with significant differences (*p* < 0.001) between groups. Four populations or ‘chemotypes’ were identified within the strains studied, and several known secondary metabolites of *Alternaria* were identified as distinguishing metabolites, including tenuazonic acid, phomapyrones, and altenuene. Though species-level identifications could not be concluded for all groups through metabolomics alone, *A. infectoria* was able to be identified as a distinct population.

## 1. Introduction

*Alternaria* is a cosmopolitan phytopathogenic fungal genus responsible for the spoilage of numerous agriculturally relevant crops of economic importance to Canada, including wheat, canola and tomatoes [1,2,3,4]. Non-host-specific secondary metabolites from *Alternaria* such as alternariol (AOH), alternariol monomethyl ether (AME), tenuazonic acid (TeA) and tentoxin (TTX) are commonly detected in processed wheat- and fruit-based commodities, including infant foods [5]. The European Union has evaluated these toxins for regulation because of their moderate cytotoxicity in vitro [5,6,7,8,9,10,11,12,13,14,15]. Owing to their structural similarities to the sphinganine analogue mycotoxin fumonisin, there is similar concern over the presence of AAL-toxins in tomatoes and processed tomato-based commodities [16,17,18].

After recent redefinitions based on whole-genome sequencing data, the genus *Alternaria* was divided into 27 taxonomic sections, some of which (such as *Embellisia*, *Nimbya* and *Ulocladium*) had previously been considered as distinct genera [19,20,21,22]. Species concepts, especially those for the most frequently cited species, *A. alternata* and *A. tenuissima*, were also critically re-evaluated; many species, *formae speciales* or pathotypes previously considered distinct because of pathogenicity to specific hosts, or morphological differences such as the length of the apical cell of the conidium, were all combined into *A. alternata* based on Geneological Concordance Phylogenetic Species Recognition (GCPSR) criteria [19]. It is important to note that even when the species of an *Alternaria* strain can be identified, the prevalence of ‘conditionally-dispensable chromosomes’ (CDC) that control the production of host-specific toxins can result in distinct ‘pathotypes’ within the same species [23]. These CDCs can disappear, or be picked up by strains through horizontal gene transfer [23,24,25,26,27]. Uncertainty regarding the genetic classification of *Alternaria* coupled with the presence of CDCs has made phylogenetic analysis and morphological characterization insufficiently reliable to predict their risk to crops or toxigenic potential [2,28,29].

As defined by Frisvad, chemotaxonomy refers to *“the classification and identification of filamentous fungi based on profiles of secondary metabolites”* [30]. Chemotaxonomy is an especially powerful tool to understand the fungal ecology and toxigenic potential of species with CDCs, or whose properties are not fully predicted by genetic markers. Advances in metabolomics, driven by innovations in high-resolution mass spectrometry (HRMS), metabolite databases and data analysis have been beneficial for the secondary metabolite screening of filamentous fungi. Fungal culture extracts can be readily screened for hundreds of known fungal metabolites by liquid chromatography (LC)-HRMS. In a polyphasic approach with morphological and molecular information, chemical data have previously been used to differentiate among *Alternaria* for both large-spored, and the less morphologically distinct small-spored species, and were critical for the delineation of the species *A*. *infectoria*, which is common in wheat, and is also an opportunistic pathogen of immunocompromised humans [2,28,29,31,32,33,34,35,36]. Untargeted LC-HRMS analysis is essential for the characterization of chemotaxonomic groups and profiling, as targeted methods may overlook unknown but related metabolites or modified secondary metabolites that are potentially important chemotaxonomically.

The need exists for a better understanding of which species of *Alternaria* occur in Canada and, equally important, is the need to determine the secondary metabolites they produce. The last major survey of Canadian *Alternaria* was published nearly 75 years ago [37]. Due to the presence of CDCs and the uncertainty surrounding *Alternaria* classification, there is an enormous difficulty in estimating the toxigenic potential of strains and the risk to Canadian consumers. We have taken a chemotaxonomic approach to investigate 128 Canadian strains isolated from wheat, apples, blueberries, tomatoes and various perennial shrubs.

## 2. Results

### 2.1. Principal Component Analysis (PCA) of Secondary Metabolites from Canadian Species of Alternaria

**Figure 1:**
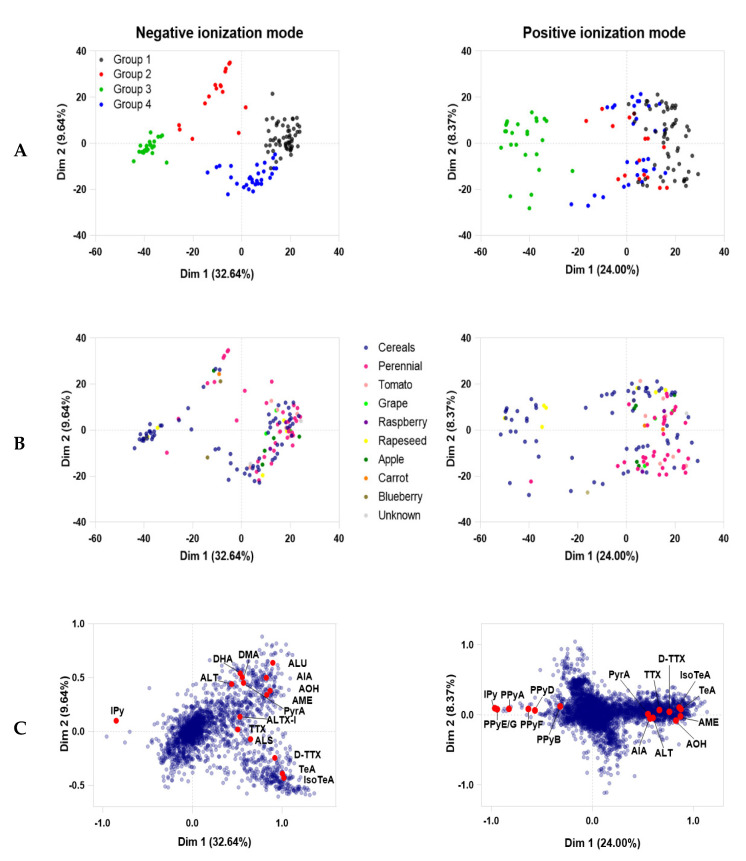
Principal component analysis (PCA) in both negative and positive ionization modes, where each point represents a single strain. (**A**) Strains in the PCA are coloured by *k*-means clustering group assignments from negative ionization mode. (**B**) PCA coloured by the substrate where strain was isolated. (**C**) Loadings plots indicating the positions of the main metabolites detected that influence the PCA separations.

Secondary metabolite data from the 128 strains of *Alternaria* analyzed by LC-HRMS in both negative and positive ionization mode were processed by PCA. Generated peak lists have been included in Supplementary A and B, and uploaded online to Metabolomics Workbench (see Supplementary). To avoid biased group assignment of the PCA plots, samples were statistically assigned into groups based on a *k*-means clustering algorithm (Figure 1A). The metabolomic analysis determined that there were four groups in negative ionization mode based on the clustering algorithm analysis and the sum of squares. Dimensions 1 and 2 represented 32.64% and 9.64% of the data variability, respectively. Clustering data were easily visualized in negative ionization mode, but it was more difficult to ascertain differences between groups 1, 2 and 4 in positive mode due to the lack of separation along the first (24.00% of the variability) and second (8.37%) dimensions. Although group 1 remained distinct between both ionization modes in the *k*-means clustering analysis, isolates belonging to groups 2, 3 and 4, as defined by the negative mode *k*-means analysis, became intermixed and indistinguishable in positive mode *k*-means analysis (Figure 1A). Thus, the groups generated by the *k*-means clustering algorithm in negative mode were assigned to the positive mode data to avoid unbiased visual group assignment.

**Figure 2:**
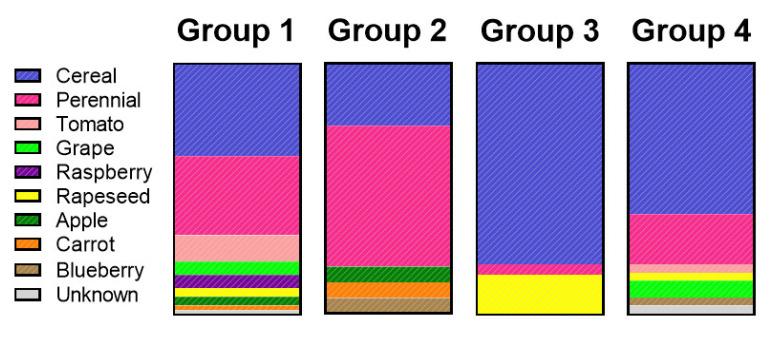
Substrate distribution within groups assigned by *k*-means clustering.

Because of the potential of *Alternaria* species to produce host-specific toxins, we investigated whether the populations observed were related to the substrate from which they were isolated. Individual isolates are coloured by the substrate they were collected from in the PCA plots (Figure 1B) and a host distribution is shown in Figure 2. Group 3 primarily consisted of isolates from grain (80%) however, more host diversity was observed in Groups 1, 2 and 4 (Figure 2). The specific metabolites from each of the assigned populations were further investigated, and are reported in Table 1. All isolates were screened for the production of AAL-toxins, but none were detected.

### 2.2. Statistical and Metabolomic Analysis of Detected Canadian Alternaria Populations

The loading plots (Figure 1C) show the individual metabolites responsible for the strain coordinates within the PCA plots, and have values ranging from −1 to +1 along both dimensions. Metabolite features that are shared by multiple chemotype groups account for less variance and weight in the PCA, have negligible (near 0) loadings, and appear close to the origin. Conversely, metabolites with the strongest influence or effects on the components in the dataset have larger values, close to either +1 or −1. Larger loading values show the metabolite directionality in the PCA plot, corresponding to the potential presence or absence of that metabolite within a particular group. Metabolites with the strongest influence were further investigated for their statistical differences between each group using the Kruskal-Wallis test. Calculated *p* values were corrected based on post-hoc Benjamini–Hochberg (BH) false discovery rate (FDR) correction.

The metabolites responsible for the majority of the variance in the PCA included the phomapyrones (*p* < 0.001, FDR corrected, see Supplementary A and B) and infectopyrone (IPy, *p* = 2.03 × 10^−12^), the amino acid derived tenuazonic acid (TeA, *p* = 4.80 × 10^−17^) and its valine-substituted iso-tenuazonic acid, (IsoTeA, *p* = 1.61 × 10^−16^), and several secondary metabolites known to be produced by *Alternaria*. Metabolites that are directly opposite to each other along the diagonal of the loadings plot are negatively correlated. For instance, the infectopyrones and phomapyrones were observed to be strongly correlated to each other, but were negatively correlated to the other dibenzopyrones, such as alternariol (AOH, *p* = 1.95 × 10^−16^), and alternariol monomethyl ether (AME, *p* = 5.69 × 10^−17^).

Table 1 shows the known secondary metabolites produced by *Alternaria*, and which chemotaxonomic groups they were detected in. Though the list is not exhaustive, it represents the breadth of known and well characterized secondary metabolites, either through analytical standards, or from published LC-MS/MS transitions. Both known and unknown significant (*p* < 0.001) metabolites have been included in Supplementary A and B, and through Metabolomics Workbench, (included in Supplementary). Most of the dibenzopyrones and commonly detected secondary metabolites of *Alternaria* are capable of ionizing in both positive and negative ionization modes, but the majority of phomapyrones, which are commonly associated with *A*. *infectoria*, ionized best in the positive ionization mode (see Supplementary). Ionization in positive mode was only able to distinguish group 3 from the other groups, as it did not share metabolites (Table 1). The PCA separation across both dimensions was best observed in the negative ionization mode. In addition to the better ionization of dibenzopyrones, the variability across the first dimension was predominantly due to the presence or absence of tenuazonic acid (TeA) (Figure 1C). Groups 2 and 3, which cluster in negative dimension 1, do not produce TeA. Both of these groups are also differentiated from each other across the second dimension due to the differentiating metabolites produced solely by the isolates in group 3, such as infectopyrone (IPy). Though group 2 does not produce TeA, it does share other common metabolites with groups 1 and 4, including alternariol (AOH) and alternariol monomethyl ether (AME) (Table 1). Variation in the second dimension of the PCA plots (Figure 1A), especially between groups 1 and 4, was largely influenced by abundances in the detected peak areas. Both groups 1 and 4 include many of the same metabolites, but group 1 tended to have higher abundances of those shared metabolites, (Supplementary A and B). The major difference between groups 1 and 4 was that group 4 did not produce altenuene or desmethylaltenusin.

Of the 4170 metabolite features detected in the positive ionization mode data, there were 1593 significant metabolites (*p* < 0.001) after applying the Kruskal–Wallis test using the assigned groups from the *k*-means clustering (Supplementary A). Similarly, there were 2198 metabolite features detected in negative ionization mode, and 1060 were significant (*p* < 0.001) (Supplementary B).

## 3. Discussion

There was little correlation between the chemotype group and the substrate of origin; a wide substrate diversity was observed within PCA groups 1, 2 and 4. Population 3 had the most unique metabolite profile. It did not produce the dibenzopyrones AOH and AME, nor tenuazonic acid. It was however, the only population that produced infectopyrone and phomapyrones. This population was isolated predominately from cereal (Figure 2) as well as some perennial and rapeseed. Based on the substrate of isolation and the production of infectopyrones, this population is likely comprised of the *A*. *infectoria* species group [31]. It is unsurprising that *A*. *infectoria* was distinguished through metabolomics methods, as it was previously reported to be a very distinct section (section *Infectoriae*) within *Alternaria* [36,38].

The remaining population groups, 1, 2 and 4, all produced varying amounts of dibenzopyrones; however, the major differentiating feature was the presence or absence of TeA, (Supplementary A and B). TeA was produced by groups 1 and 4, but absent in group 2. TeA production has been previously linked to a non-ribosomal peptide synthetase and polyketide synthase (PKS) hybrid enzyme, of which the ketosynthase tenuazonic acid synthetase (TAS1 KS) domain was reported to be imperative for the production of TeA [39]. Similarly, the production of alternariol (AOH) has been correlated with the expression levels from the polyketide synthase gene, *pksJ* [40].

As PCA is particularly sensitive to the relative abundances of metabolites, it was challenging to discern many differences between groups 1, 2 and 4. Andersen (2015) previously reported that production of metabolites was inconsistent between isolates, even for the common dibenzopyrones AOH and AME, making differentiation by metabolomics alone much more difficult [2]. It is unclear whether the observed separation of the 128 *Alternaria* isolates is a consequence of species-level differences, PKS mutations, gene expression levels, or if dispensable chromosomes coding for host-specific toxins were present. There are also extenuating environmental conditions, because there are apparently marked differences between Canadian strains, and other strains of *A*. *infectoria* isolated from around the world; Canadian strains did not produce the novae-zelandins, as reported previously for this species [2,29,31]. The apparent lack of substrate specificity within the groups suggests that the chemotypes identified herein could be detected on numerous commercial commodities. The substantial population of *A*. *infectoria* present on wheat and cereal crops indicates that its secondary metabolites may need to be monitored alongside the other food-relevant *Alternaria* secondary metabolites in produce and processed commodities. While the European Food Safety Authority is considering regulating several *Alternaria* secondary metabolites, including TeA and TTX, there are currently no regulatory limits.

## 4. Materials and Methods

### 4.1. Fungal Material and Identification

The 128 strains of *Alternaria* spp. studied in this work were obtained from the Canadian Collection of Fungal Cultures (CCFC) in Ottawa, Ontario, the Canadian Grain Commission (CGC) in Winnipeg, Manitoba or isolated from local sources. The majority of the strains were isolated from food-relevant crops, including grain, apples, tomatoes and grapes, although several strains were isolated from various perennial shrubs, (Table S1, Supplementary C). Individual isolated strains not obtained from CCFC or CGC were identified to the genus level based on morphology. To identify the species, partial DNA sequences for either RNA polymerase II second largest subunit (RPB2) or complete sequences of rDNA internal transcribed spacers (ITS) were determined for 108 of the strains using the primers, amplification and sequencing parameters of Woudenberg et al. [19]. The data were examined by J. Woudenberg of the Westerdijk Fungal Biodiversity Institute, Utrecht, the Netherlands in comparison with her reference data (Supplementary C).

### 4.2. Agar Plug Extraction

Potato dextrose agar (PDA) was selected for metabolomic analysis due to the large diversity of metabolites produced in comparison to the other culture media tested (data not shown). Strains were transferred as 3-point inoculations onto PDA plates (Sigma Aldrich, St. Louis, MO, USA). Cultures were incubated at 25 °C in darkness for seven days, during which the majority of the strains reached approximately 4 cm in diameter. Following incubation, six agar plugs were removed from each 3-point inoculum using a 6 mm cork borer, followed by extraction by ethyl acetate containing 1% formic acid (Sigma Aldrich, St. Louis, MO, USA). Prior to LC-MS screening, extracts were dried under nitrogen before reconstitution in acetonitrile, and filtration with 0.45 µm PTFE syringe filters (ChromeSpec) into vials [35,41].

### 4.3. LC-HRMS Analysis

High-resolution mass spectrometry (HRMS) data were obtained using a Thermo Q-Exactive Quadrupole Orbitrap Mass Spectrometer coupled to an Agilent 1290 HPLC. Chromatography and mass spectrometry conditions for both polar and nonpolar compounds were previously optimized [16,42]. For chromatographic separation, a Zorbax Eclipse Plus RRHD C18 column (2.1 × 50 mm, 1.8 μm; Agilent) was maintained at 35 °C using mobile phases comprised of water with 0.1% formic acid (A), and acetonitrile with 0.1% formic acid (B) (Optima grade, Fisher Scientific, Lawn, NJ, USA). Mobile phase B was held at 0% for 30 s, and increased to 100% over three and a half minutes. B was held at 100% for 1 and a half minutes, before returning to 0% B in 30 s. Injections were made at a volume of 2 μL and a flow rate of 0.3 mL/min was used. The following conditions were used for negative HESI for full MS: capillary voltage, 3.7 kV; capillary temperature, 400 °C; sheath gas, 17.00 units; auxiliary gas, 8.00 units; probe heater temperature, 450 °C; S-Lens RF level, 45.00; AGC target, 1e6; maximum injection time (IT), 512 ms; scan range *m*/*z* 100–1200. Similar conditions were used for positive HESI, but with a capillary voltage of 3.9 kV. HRMS data for both positive and negative full MS data were acquired at a resolution of 140,000 and maximum IT of 500 ms. Lock masses of Di-n-butyl phthalate (DBP) and sodiated formic acid (FA) dimer were monitored in positive and negative ionization modes respectively to account for instrumental drift.

### 4.4. Principal Component Analysis (PCA) and k-Means Clustering Analysis

Raw HRMS data generated in both positive and negative mode were converted to mzml files and centroided prior to XCMS processing in R (r-project.org) to generate peak lists (see Supplementary) [43,44,45]. The applied XCMS conditions for the production of the peak lists are listed in Supplementary C. Sample carry-over was corrected according to the maximum peak areas detected in the blanks. Due to the minfrac XCMS settings, lower intensity metabolites not present in at least 25% of the sample dataset were not counted as peaks. Thus, the presence or absence of metabolites was confirmed through analysis of the raw data files. Any metabolites from the peak list with peak areas equal to zero were replaced with 2/3 of the minimum peak area value of all metabolites detected [46]. Following zero replacements, a log transformation of the peak areas was performed. Pareto scaling was used to adjust for fold differences between metabolites during PCA generation using MetabolAnalyze and FactoMineR packages. Unsupervised groups from the PCA were assigned by *k*-means clustering analysis using scripts adapted for R [47]. From the logged peak lists of the metabolite datasets, the appropriate *k*-means cluster was investigated for up to six groups using the elbow method of the within sum of squares (WSS) plot. Variations identified by PCA and *k*-means clustering analysis between samples were investigated through statistical analysis of metabolomic data.

### 4.5. Metabolomic Analysis

Each metabolite in the peak list was investigated for its significance using the Kruskal–Wallis test with post-hoc Benjamini–Hochberg (BH) False Discovery Rate (FDR) correction with a threshold value of *p* < 0.01 to account for multiple hypothesis testing. Metabolites with FDR corrected threshold *p* values < 0.001 and log2 of the average peak area values > 1 were further investigated within each group assignment. Significant metabolites were confirmed through comparison to authentic standards, or to the published literature MS/MS data. All unknown metabolites were searched using SciFinder and Antibase 2013. 44 of the 128 strains were subsequently re-cultured, re-extracted and re-analyzed, as described above, to ensure that the metabolomic analyses were consistent.

## 5. Conclusions

Four main chemotaxonomic populations of Canadian *Alternaria* were identified, including *A*. *infectoria*, which was largely present on cereals and rapeseed. Substrate had no apparent effect on chemotaxonomy, though there were marked metabolite differences between Canadian *Alternaria* strains and other reported strains from around the world. Distinguishing secondary metabolites including IPy, the phomapyrones, ALT, DMA and TeA may assist in future organizations of the genus. Identification of *Alternaria* species and linking them to secondary metabolite production remains a great challenge. More work is needed from a mycology perspective with respect to their taxonomy. Metabolomic profiling provides another key component to help aid in identification of *Alternaria*, and more importantly provides critical insight into the toxigenic potential of these strains, and the risk to consumers.

## Figures and Tables

**Table 1:** Known *Alternaria* metabolites detected in 128 Canadian strains of *Alternaria* separated by their population, and percentage of isolates producing them.

Metabolite	Abbr.	RT (min)	Populations of Canadian Strains (% of Strains)			
			Group 1 (*n* = 57)	Group 2 (*n* = 16)	Group 3 (*n* = 25)	Group 4 (*n* = 30)
infectopyrone	IPy	3.03	-	-	100	-
phomapyrone A	PPyA	3.73	-	-	100	-
phomapyrone B	PPyB	2.83	-	-	85	-
phomapyrone D	PPyD	3.04	-	-	100	-
phomapyrone E/G	PPyE/G	2.99	-	-	100	-
phomapyrone F	PPyF	3.01	-	-	50	-
tenuazonic acid	TeA	3.05	100	-	-	100
iso-tenuazonic acid derivative	IsoTeA	2.81	100	-	-	100
alternariol *	AOH	3.1	100	94	-	91
alternariol monomethyl ether *	AME	3.53	100	100	-	94
altenusin *	ALU	2.97	100	71	-	55
desmethylaltenusin *	DMA	2.75	23	18	-	-
dehydroaltenusin *	DHA	3.26	72	41	-	9
alternarienoic acid *	AlA	2.71	100	71	-	30
altenuene	ALT	2.87	70	53	-	-
altechromone A	ALCA	2.82	79	53	-	30
altechromone B	ALCB	2.72	98	82	-	12
altertoxin I *	ALTX-I	2.53	33	59	-	36
altertoxin II *	ALTX-II	2.93	16	35	-	30
altertoxin III *	ALTX-III	3.27	26	18	-	9
tentoxin	TTX	3.11	30	29	-	21
dihydrotentoxin	D-TTX	3.13	33	24	-	15
altersetin *	ALS	4.37	68	53	-	67
pyrenochaetic acid A	PyrA	2.63	93	71	-	21

* Analysis from negative ionization mode data.

## Supplementary Materials

The following are available online at https://www.mdpi.com/2218-1989/10/6/238/s1. Raw data and peak list files are available at the NIH Common Fund’s National Metabolomics Data Repository (NMDR) website, the Metabolomics Workbench, https://www.metabolomicsworkbench.org where they have been assigned Study ID ST001370. They can be accessed directly using Project DOI 10.21228/M8Q114. Supplementary A: List of 1593 significant metabolites by their *m*/*z* (*p* < 0.001) as calculated by the Kruskal-Wallis test from the positive ionization mode dataset; Supplementary B: List of 1060 significant metabolites by their *m*/*z* (*p* < 0.001) as calculated by the Kruskal-Wallis test from the negative ionization mode dataset; Supplementary C: List of 128 *Alternaria* strains used in the metabolomic analysis, including RPB2 primer analysis & xcms conditions used in R.
