# Supplementary material for: Chemotaxonomic Profiling of Canadian Alternaria Populations Using High-Resolution Mass Spectrometry
Source: Metabolites. 2020 Jun 9;10(6):238. doi: 10.3390/metabo10060238 (PMC7345142; doi:10.3390/metabo10060238)
Supplement: Supplementary file 1 [file metabolites-10-00238-s001.pdf]

Supplementary A

Kruskal-Wallis significant metabolites positive ionization mode

[M+H]+	RT (mins)	P value	fdr corrected P value	Group 1 avg peak area	Group 2 avg peak area	Group 3 avg peak area	Group 4 avg peak area
214.05083	2.139767	0.000998	0.002612436	693973.2235	527076.9401	439068.8414	740404.9541
328.91547	4.3568	0.000996	0.002608546	16989459.05	15000997.41	39330964.29	19345945.52
364.18423	2.502483	0.000969	0.002538992	293821.7449	301459.9899	715192.2513	431590.0779
120.04468	2.705258	0.000943	0.002471928	1034837.687	1270351.609	745292.1842	2461414.702
333.2336	3.913392	0.000935	0.002452431	2684924.236	4806873.12	1073771.139	2036508.058
497.28747	3.604425	0.000934	0.002452431	611390.5486	1007706.125	178178.0999	408402.466
184.56017	2.369492	0.000928	0.00244023	1729159.557	1519252.39	2279557.142	1043180.906
341.28719	4.303467	0.000929	0.00244023	2892724.29	2971079.985	2270063.319	2864094.528
132.03531	4.364133	0.0009	0.00236854	19871372.53	18811910.78	39326070.8	20774132.4
196.01683	3.28415	0.000898	0.002364778	12651063.59	12872587.87	19494433.83	12000806.28
183.11283	2.176867	0.000886	0.002334127	618480.4673	572972.5982	914297.9417	711155.9161
216.01795	0.940382	0.000886	0.002334127	1151953.55	957288.3666	2216101.4	1858061.727
316.28463	4.102533	0.000865	0.002281998	457762.9509	453998.478	228771.8346	580955.6753
327.15507	2.537967	0.000865	0.002281998	1757674.222	664675.4022	946367.2511	1047244.101
549.13288	2.7388	0.000849	0.002241491	495702.29	594181.8251	4067214.953	337038.8575
175.06286	2.329567	0.000844	0.002230584	1047109.973	1019922.673	2258702.605	1434152.553
343.12984	2.396792	0.000835	0.002207726	15939585.73	11166916.82	11328214.37	11403313.83
183.98858	4.329783	0.000829	0.002192608	9318689.192	13057357.88	17912208.57	10050005.96
200.20091	4.628583	0.000823	0.002178108	12929615.27	15724173.34	11284698.72	10492145.64
276.13191	2.105358	0.000818	0.002166821	2759432.675	1872396.144	1179693.205	1298560.623
279.23193	4.718392	0.000808	0.002142575	2752781.094	2132525.554	10848177.36	3212521.563
142.0975	5.873883	0.000805	0.002135311	6574047.745	5941805.199	9282444.967	5862616.712
255.21074	4.529958	0.000803	0.002130361	912294.5307	1222501.51	1561438.702	701920.1788
265.02308	0.463363	0.000802	0.002129147	16698803.16	28715291.73	14574399.34	20773116
321.24248	4.719867	0.000799	0.002124186	2917222.615	1709530.446	6267035.232	2861475.721
322.10748	2.819492	0.000799	0.002124186	1748522.144	1575492.243	654221.6689	1208938.251
326.17101	2.32195	0.000787	0.002094347	730255.1255	245366.0224	786865.9879	926952.1275
208.11711	3.458458	0.000785	0.00209149	316672	251706.1858	395339.2631	147582.1875
179.09277	5.193183	0.000784	0.002088002	19462382.3	18371978.94	22483944.76	21706944.25
212.08987	3.407283	0.000783	0.002088002	687198.2608	289350.5207	724396.2641	581730.1434
181.09714	2.38395	0.000779	0.002077779	1634634.291	1805434.478	2430913.743	1955747.828
254.61666	3.6303	0.000774	0.002065494	330395.3782	684818.8668	44645.20564	305427.4684
379.33603	4.521883	0.000774	0.002065494	94535092.75	152755398.2	275112818.1	90716045.91

Kruskal-Wallis significant metabolites positive ionization mode

189.007	1.16794	0.000763	0.002039774	4834379.034	4692834.414	6781440.197	3698760.18
425.23961	2.646317	0.000752	0.002011845	270751.9374	362638.0128	108155.6974	189971.1165
313.92876	4.321317	0.000746	0.00199789	3175081.305	2618707.803	6412347.29	4356612.788
302.96989	1.799717	0.000733	0.001962977	517676.1049	218420.7118	310693.4601	661168.3573
332.19125	3.192633	0.000726	0.001946496	1429788.957	1998769.389	561699.5359	1948461.627
335.21922	3.143342	0.000718	0.001925231	3099958.249	1620611.476	1197811.845	2809813.184
352.24576	2.881558	0.000713	0.001914588	524965.8743	340057.4501	423428.2154	520621.6488
514.28581	3.283058	0.000713	0.001914588	591848.1994	507766.6443	129115.3536	393011.8037
255.94406	0.040663	0.000707	0.001899965	8128.32069	8263.241324	285301.0062	31724.9494
429.26114	4.713233	0.000707	0.001899882	350564.6417	260746.1441	130967.5432	155211.1221
110.52203	3.832583	0.000696	0.00187168	18774787.25	16326903.11	33688531.74	17379915.34
339.25312	4.720517	0.000688	0.001850903	7644358.9	5312274.087	18844445.13	7513909.877
369.29763	4.97535	0.000672	0.001811194	1727490.685	1728769.516	489472.7729	1030202.893
121.06508	2.332625	0.000666	0.001795712	3292868.326	3732101.373	4234074.35	5487382.476
500.38555	4.1988	0.00065	0.001754005	1385391.057	1399983.875	1836665.478	719942.544
340.25644	4.713808	0.000639	0.001723521	1474649.751	921233.66	3605368.792	1376374.625
287.88881	4.31225	0.000634	0.001711436	2508558.618	2489220.978	6984763.603	2852592.582
494.34528	4.147167	0.000628	0.001697829	345529.8109	474442.6156	179127.1829	254774.7686
174.05833	2.137942	0.00062	0.001675527	332753.7029	208186.5506	245081.7294	537091.269
176.07066	2.803083	0.000618	0.001673506	648622.296	541851.6484	361811.2353	682676.2834
307.1904	2.97725	0.000614	0.001661791	2241456.248	2592439.18	965075.0133	2722307.78
239.03387	0.924367	0.000613	0.00166133	466672.7951	501317.1568	997164.5528	841505.4427
327.08631	2.368967	0.000612	0.001659476	824071.5043	729056.5424	1042117.584	526546.9239
145.10118	3.561983	0.000608	0.00165029	485609.0571	905110.4173	287450.1079	463159.7105
110.02045	3.81485	0.000602	0.001633446	279615590.1	214948867.4	481100802	292528580.5
324.26149	4.716558	0.000599	0.001626938	260474.1026	178565.1316	666754.9815	270199.6345
102.97074	0.632984	0.000591	0.001605225	19798927.02	18814531.27	15900972.21	16927104.1
146.0601	2.227383	0.000589	0.001603387	6448552.142	4476594.015	12139889.26	7381845.255
254.13867	2.750225	0.000589	0.001603387	626843.6104	351004.9308	1194804.999	567547.6589
239.16418	2.8905	0.000585	0.001594681	576145.9432	592351.1584	1309352.054	504030.1389
294.21453	3.701583	0.000576	0.001568615	1014116.905	1205860.731	371055.3995	1808333.231
144.98221	2.089717	0.000566	0.001542463	42247014.39	45495688.91	46235945.83	73032228.87
373.32474	4.1991	0.000563	0.001536208	455468.3716	328584.8996	206255.3688	442300.7227
130.05002	0.760997	0.000543	0.00148168	7411177.813	14234641.91	10393699.89	20394716.16

Kruskal-Wallis significant metabolites positive ionization mode

286.13658	2.558883	0.000537	0.00146844	967425.6432	1579721.004	618059.3588	650896.3326
511.36156	4.5305	0.000523	0.001429051	302874.8577	421347.123	1477396.296	270674.9194
378.32322	4.530483	0.000515	0.001410495	1510514.373	1766540.102	3997354.458	1610476.068
269.05906	2.601883	0.000514	0.001407069	317100.7788	135236.3527	407430.7622	350078.9371
273.16714	2.596383	0.000513	0.001404422	656586.9566	902954.3435	661567.9284	660402.3712
403.24796	3.970267	0.000508	0.001393161	752078.2618	795504.1398	465233.5097	919139.3976
179.07027	3.553042	0.000505	0.00138509	840882.4579	2559355.396	492289.4896	712466.774
146.98038	2.750892	0.000502	0.001377388	41772704.68	22370835.27	19585621.87	33832732.57
263.16429	4.486867	0.000478	0.001311997	1806414.12	2838945.15	1236941.419	1286846.982
475.3056	3.919625	0.000459	0.001261105	556534.4435	1198214.361	1184411.73	661917.0799
574.25082	2.6025	0.000454	0.001248669	73771.33839	87242.3914	5100.279302	24459.12609
407.29453	4.7313	0.000448	0.001232821	926932.2676	826535.3856	344268.3412	627373.1868
426.29071	4.713525	0.000442	0.00121706	12556942.15	10480811.28	9081904.339	12840719.88
343.22692	3.269967	0.000427	0.001176877	13688436.01	22830132.58	2658567.552	6459796.187
299.11829	2.593983	0.000419	0.00115516	3801058.75	2718043.796	2402764.371	1361810.531
315.23195	4.914267	0.000413	0.001140533	1682371.32	2253625.118	5661363.689	3918132.947
372.17654	2.256433	0.000413	0.001140533	222739.1654	182141.8479	564497.3172	270644.375
174.11254	2.489083	0.000408	0.001127971	4246967.169	2770655.875	6080657.315	10545209.05
314.3053	3.48405	0.000408	0.001127971	616135.7796	545433.6304	539223.714	723222.0493
276.95436	0.665263	0.000399	0.001104963	1940856.058	1306603.083	5076708.384	4362913.99
151.03891	2.59465	0.000398	0.001100661	3197172.038	5519167.7	7733920.018	6606170.566
313.21616	3.230967	0.000394	0.001092131	828719.095	1315874.286	588715.3485	965155.9675
210.18524	3.247933	0.000393	0.001089511	441043.5682	441768.8309	488487.4571	505644.9647
290.26901	3.117267	0.000392	0.001086237	1822087.573	1099454.151	1252197.832	2105796.503
254.10228	2.369817	0.000377	0.001045317	767914.7368	612506.9928	1853165.544	796114.5191
268.118	2.75915	0.000375	0.00104222	1017271.796	639893.7304	1213012.911	826326.1347
285.14439	2.5589	0.000371	0.001033075	6834636.721	12358553.24	3877785.555	4536307.328
423.28948	4.329367	0.000372	0.001033075	652996.6138	699882.4454	182171.3349	314312.61
277.07904	3.125167	0.000361	0.001005907	1996575.391	2074155.616	1385108.493	5596910.67
510.35814	4.530308	0.000359	0.000999195	1259620.401	2019698.573	4676565.493	1231122.546
215.09149	3.549133	0.000358	0.000997364	2670937.182	8566068.897	1159991.733	1924690.821
425.28739	4.713483	0.000355	0.000991373	55716285.25	46976174.91	40671151.77	56780203.17
293.21107	4.137583	0.000355	0.000991314	8281455.596	8382738.684	2996869.132	15303757.81
463.28195	4.91425	0.000346	0.000966964	662753.1688	811243.0601	2280203.579	1491381.127

Kruskal-Wallis significant metabolites positive ionization mode

322.25036	4.717067	0.000346	0.000966177	1744417.461	1229734.791	4141274.435	1802277.011
449.25108	3.187192	0.000344	0.00096141	479546.4977	243592.3475	58087.21657	367468.7954
266.13632	2.558933	0.000343	0.000959835	610953.4725	757346.6617	1270678.313	651225.3999
408.15547	3.282158	0.000339	0.000949265	84048.0458	62069.901	403646.7258	143324.2011
537.22439	4.382033	0.000336	0.000942896	243964.3796	138176.0329	15920.68832	288394.0485
385.09205	0.452042	0.000334	0.000935542	400186.3202	1236718.78	661890.8168	614398.6046
321.14787	2.392767	0.000332	0.000932464	1834011.359	1030644.883	1226042.561	1186305.615
118.08656	0.764192	0.000327	0.000916854	14063406.68	12771481.24	20875206.95	17042525.86
229.11835	2.193833	0.000322	0.000905135	4770000.432	5326437.711	7692020.364	9491210.896
499.38241	4.203175	0.000319	0.000898105	9927078.527	10062573.05	12969809.61	5075610.294
240.12304	2.599533	0.000314	0.000884222	522031.9977	444395.4073	543351.0089	306698.3526
316.19641	4.09395	0.000304	0.000855416	236206.0469	263186.6783	73626.50572	521629.6413
156.1019	2.283833	0.000299	0.000842288	2197027.179	1665517.668	1608550.66	1884145.443
197.04152	3.544558	0.000298	0.000840362	946835.0476	1165625.04	804660.3769	523968.2714
293.21109	3.702367	0.000296	0.000834911	7305070.746	8046061.361	3063093.385	12444816.18
446.17069	4.4881	0.000293	0.000826342	184654.7062	517111.7554	11995.09758	241967.4878
309.08695	2.950942	0.00029	0.000818934	954008.9817	339378.6716	1501576.299	649775.1738
496.33989	4.171817	0.000279	0.000789362	1710691.539	1409704.217	3718717.8	1344655.507
463.30306	4.355617	0.000272	0.000769187	450320.4891	599941.3154	201026.4689	720876.8932
307.15407	2.932133	0.000269	0.000762788	1108680.652	1368663.353	521385.5972	1162230.571
464.28547	4.914275	0.000266	0.000753361	130619.4404	161110.6815	580565.0575	371759.2174
179.02264	1.185003	0.000263	0.000745186	1313052.05	1113489.073	1633362.042	1092882.861
393.22701	3.003633	0.000261	0.000740722	1894128.921	2415541.4	689606.0829	1440963.742
491.30045	3.20425	0.000258	0.000732918	339565.0982	562286.0188	94601.70313	226602.5708
324.21686	1.7991	0.000257	0.000731229	59837967.58	63359079.76	81939667.05	52519722
334.11549	3.631692	0.000257	0.000731011	2235028.079	645636.267	611719.8343	2502858.444
444.33809	4.746242	0.000256	0.00072767	73324.32931	37754.46215	321480.6522	23479.76863
263.16425	3.432992	0.000252	0.000717845	536947.7998	904542.5446	289798.1394	457179.1839
120.98109	0.633592	0.000252	0.000717575	17543299.38	16902167.61	14370733.34	14808177.81
393.31513	3.728317	0.000247	0.000703009	650370.1721	904794.6463	1390102.818	820318.6131
299.05264	2.536017	0.000239	0.000682945	2908491.517	2081890.642	2325653.4	880893.4352
380.2513	3.243967	0.000231	0.000658069	8890363.27	16153214.59	1126828.238	4940788.908
235.12245	3.231817	0.00022	0.00062813	2737944.617	6709676.818	1733934.823	1834857.434
364.17955	3.112775	0.00022	0.00062813	2018849.959	5434570.45	924880.1314	1385486.336

Kruskal-Wallis significant metabolites positive ionization mode

355.24796	4.716225	0.000216	0.000617065	1900371.608	952476.9617	5182731.799	1575135.463
305.17482	3.289417	0.000215	0.000613936	994161.0578	1250154.991	468914.7356	1446606.766
200.2009	5.001383	0.000209	0.000599028	12958943.86	16406880.58	10577851.81	9825273.578
140.0682	3.0819	0.000205	0.000586543	5735663.945	3947150.022	7347314.987	8055445.005
291.19545	3.191492	0.0002	0.000573228	2737152.828	3498468.873	1422631.242	3512882.406
483.17099	3.562017	0.000199	0.000571573	114952.6436	470298.6159	45358.28843	274464.9844
325.21621	3.314333	0.000198	0.000568412	768529.1669	667034.1314	360120.5946	805121.0297
207.01759	1.175908	0.000196	0.000564537	3578177.703	3083527.107	5001074.528	2689152.186
547.13487	2.74105	0.000196	0.00056442	1056726.719	665682.5898	8196109.662	357875.6918
445.16734	4.486375	0.000191	0.000549815	1316784.212	3682522.34	67419.37009	1178235.692
609.44816	5.490017	0.000188	0.000542318	1398972.054	1957377.757	930532.3694	1108682.541
146.98036	2.083583	0.000188	0.000542174	16209145.78	18714944.39	11594011.06	29300396.79
294.2145	4.107467	0.000188	0.000542174	1358184.454	1272651.027	416578.4329	2480976.818
398.17116	2.733583	0.000186	0.000536042	468483.2603	617194.1374	112325.7096	285760.8236
392.25119	4.162967	0.000181	0.000522522	804751.3244	1255187.009	417160.5596	721178.0788
255.02875	0.80483	0.00018	0.000521637	836913.654	970005.1791	1110767.557	1512420.648
300.17061	2.775542	0.000181	0.000521637	216469.0209	247089.513	297465.601	480897.7599
353.30505	4.631817	0.000179	0.000517129	1930201.354	1345146.414	921276.7045	1085250.336
514.38938	4.408467	0.000177	0.000513222	76063.84049	253391.9657	662403.7036	155733.1514
853.40837	4.379408	0.000177	0.000511668	62306.86531	15939.14016	1422.497443	64807.91715
479.27696	3.687667	0.000172	0.000499561	130537.0249	88100.74556	395702.8473	306586.8753
303.1016	3.696633	0.000166	0.000482322	224589.0227	199034.3091	845627.5748	267644.0471
157.05732	2.5505	0.000163	0.000473466	521554.1701	271951.5279	421000.5228	530654.7491
453.16744	3.627883	0.00016	0.000466188	584266.9206	575530.9758	970289.0375	520458.3617
175.10779	2.376067	0.000159	0.000461941	2016011.707	1536878.962	5021387.134	3173986.5
175.02373	0.757248	0.000157	0.000455584	1455790.002	1632166.468	2584793.899	1669857.35
326.92016	4.312283	0.000155	0.000452653	1769205.134	1544533.681	5215983.704	1982352.864
528.36859	4.530392	0.000153	0.000445157	188808.1723	317301.7466	1031292.763	143078.0127
279.64705	3.424733	0.000152	0.000444003	386023.1226	468667.8782	862749.8269	167134.2426
341.19001	2.445925	0.000144	0.000420604	373381.1399	262665.9537	714334.0889	429509.8103
289.17988	4.385383	0.000144	0.000419403	330723.1027	674739.9569	63376.15896	295202.5132
127.07551	2.10515	0.000143	0.000418759	1094486.122	968939.9672	634434.3742	771017.8246
261.62444	4.490217	0.000142	0.000415758	107929.6303	332864.6685	7148.823548	117026.5012
401.20894	3.788817	0.000142	0.000415758	925290.2671	1558686.486	193311.3906	870649.4196

Kruskal-Wallis significant metabolites positive ionization mode

379.24797	3.239633	0.000139	0.00040809	41009938.03	73429599.42	7080072.407	25257876.49
201.16378	3.56205	0.000139	0.000407666	724651.984	1280048.624	416702.1836	594615.636
242.1023	2.663217	0.000138	0.000404546	414176.748	460365.3957	655896.5553	539887.3411
344.19393	3.116267	0.000137	0.000401828	1821830.567	1566010.513	442795.5871	1488632.669
316.11804	2.63625	0.000136	0.000399478	949591.008	493788.6402	1318084.388	945315.284
145.05103	1.903283	0.000134	0.000393836	433763.8496	377291.026	1722029.842	752211.164
299.13907	2.52605	0.00013	0.000382905	3710150.146	2255744.12	3032327.985	1192173.399
362.16858	2.446158	0.000129	0.000378788	439166.8851	274408.6402	902682.1613	563851.3652
331.22679	3.911583	0.000128	0.000376681	135975887.1	250322754.7	19335297.61	84907815.21
398.26193	3.121508	0.000127	0.000374078	733128.8096	1660998.693	151361.8425	560056.3151
243.13401	2.393175	0.000125	0.00036895	10051121.13	13501682.96	19491249.38	26644434.65
486.35798	4.91415	0.000125	0.00036895	163431.1321	175233.5793	1016783.647	572234.2738
284.2948	3.62215	0.000124	0.000366523	937343.2379	1925502.307	753567.8588	441877.8779
244.13733	2.4022	0.00012	0.000353248	1047862.691	1167000.29	2002185.08	2589766.063
183.55235	2.462717	0.000118	0.000347719	527470.903	599852.3105	800193.4237	632876.7941
475.38245	3.335083	0.000117	0.000346633	1529537.822	1856025.036	2869312.527	693351.4534
201.12333	2.085033	0.000113	0.000335125	260671.1126	241048.5871	317004.8164	607219.4502
152.10697	2.422642	0.000112	0.000331203	709942.4438	606995.1681	482372.3174	565794.6583
142.08629	2.05	0.000111	0.000329378	1321454.105	1284679.279	944983.0232	2078552.881
292.10258	2.163383	0.00011	0.000327223	220936.9365	102177.66	586145.754	209423.1114
218.95863	0.966868	0.000109	0.000324464	814635.48	928755.4818	1585439.787	1021834.831
182.07075	2.401767	0.000108	0.000320164	1157730.712	1325591.791	1801479.89	2407568.532
180.04443	2.968967	0.000107	0.000318284	4976496.445	5173332.92	6341549.123	6386256.746
331.33996	3.553017	0.000106	0.000316288	1578986.569	1542730.613	2414188.756	1839003.207
345.12926	2.481	0.000102	0.000304741	782091.242	627402.526	1405676.503	839546.7633
424.24426	3.096058	0.000102	0.000303521	98824.99497	222765.659	673937.5011	134403.6014
193.03429	0.73667	9.53E-05	0.000284251	1305546.025	1352413.502	2217110.632	1934962.722
156.98083	0.957405	9.44E-05	0.000281745	907148.5693	1032404.924	1692026.977	1497752.892
332.2301	3.9127	9.32E-05	0.00027838	31081683.28	56716997.77	3272504.44	18582635.73
202.14376	2.819683	9.14E-05	0.000273275	1413853.326	1215986.092	1931349.089	2856088.328
191.08489	2.39305	8.86E-05	0.000265291	5879736.061	3552129.588	3814534.535	3569130.413
213.56285	2.358767	8.87E-05	0.000265291	142772.4255	73076.13876	610166.2816	201836.335
287.63318	3.431817	8.85E-05	0.000265072	275134.7523	339120.3179	1004306.236	138332.1014
212.00779	0.752817	8.82E-05	0.000264536	400328.2016	177447.1278	1607545.735	1483365.864

Kruskal-Wallis significant metabolites positive ionization mode

244.15442	2.556833	8.71E-05	0.000261418	481164.9196	350541.2881	204292.3902	370803.145
474.37909	3.338817	8.52E-05	0.000255666	6246790.616	5924005.5	10651001.23	2808406.033
336.22255	3.424667	8.48E-05	0.00025464	392621.7279	366606.7401	87113.51557	489347.8789
253.17982	3.8804	8.20E-05	0.000246602	747499.2659	608341.0732	404174.8937	443049.5061
236.09172	2.35275	8.16E-05	0.000245537	500980.5671	194859.7112	622446.3808	483574.1916
255.12274	2.50135	8.12E-05	0.000244512	1096169.464	542859.4632	2749871.44	944620.1464
295.24211	4.528217	8.09E-05	0.00024363	1747466.123	3623671.836	3685913.45	1603098.341
323.18539	2.8883	7.96E-05	0.000240034	1193886.872	1375125.632	463319.2046	1519633.927
345.24256	4.413692	7.87E-05	0.000237544	179104860.1	427654471.6	17353438.98	92807767.4
346.24587	4.427767	7.86E-05	0.000237326	42339672.53	101221849.5	3359961.26	19931557.19
318.01319	0.757913	7.73E-05	0.000233567	337479.8768	203472.8966	588962.8027	549591.3084
303.13727	2.392633	7.35E-05	0.000222349	1716830.486	1016771.026	1115995.294	1084094.667
238.21655	3.67555	7.04E-05	0.000213136	783130.7016	743885.4293	926430.8658	899141.491
199.06287	2.484992	6.86E-05	0.000207617	1113428.444	886048.2349	1447695.645	1859082.497
396.21307	2.715833	6.78E-05	0.000205444	726399.4253	888079.7788	346338.4439	384154.6132
228.13431	2.122683	6.71E-05	0.000203578	265964.1378	210211.6698	370373.1483	641852.1223
182.07886	2.050433	6.67E-05	0.000202435	4399977.735	5348639.343	3288223.991	9755846.366
273.08174	0.461392	6.57E-05	0.000199643	748133.1013	956051.7423	288992.6754	539018.9297
228.99072	4.528217	6.48E-05	0.000196999	1102542.961	1428065.081	3650883.147	1090725.868
248.99176	0.751005	6.44E-05	0.000195951	2133481.89	1403155.315	4988700.243	4830499.796
118.08655	2.11585	6.39E-05	0.000194503	5734771.519	5316185.825	7260301.284	5788497.882
131.08564	2.636883	6.36E-05	0.000193828	1135613.32	754266.2142	488144.3727	903048.828
672.5258	4.625267	6.36E-05	0.000193828	655312.7489	785712.2583	520295.0229	659535.9454
346.24585	3.820967	6.11E-05	0.000186245	7179667.887	11865078.59	559974.3343	2559484.176
201.16379	3.178233	6.09E-05	0.000185854	673241.4452	1313806.255	317892.7855	413738.5365
361.16047	2.457958	5.97E-05	0.000182365	271963.5958	217254.5023	656329.145	320663.6249
239.07028	2.045692	5.94E-05	0.000181674	570888.0979	667615.3255	589038.5618	1210740.463
393.31518	5.367767	5.93E-05	0.000181559	97470755.88	112254538.7	45145915.18	98917287.7
409.29489	4.155258	5.93E-05	0.000181559	476090.828	309152.1795	793917.3253	385758.0751
444.1766	2.925633	5.83E-05	0.000178619	145914.5983	40483.15708	549507.3488	116605.4826
255.95151	4.530358	5.77E-05	0.000176836	141538.8285	302721.533	819452.3102	170285.7983
351.08635	2.74265	5.66E-05	0.000173717	4219616.419	4185041.482	844750.0933	3963422.89
255.134	2.550017	5.60E-05	0.000171906	1094742.458	570253.8645	2610406.056	858865.2955
261.00913	0.745823	5.48E-05	0.000168514	618441.044	347239.0369	1195147.983	1205292.406

Kruskal-Wallis significant metabolites positive ionization mode

452.2772	3.786517	5.22E-05	0.000160622	779121.7141	960276.0016	337378.4087	796183.5551
338.26891	4.103183	5.12E-05	0.000157629	1312580.388	932226.6613	395165.7118	2200105.134
184.13323	2.642567	5.11E-05	0.000157382	2649523.461	1223957.045	994281.6908	3666814.625
227.13899	2.4096	5.10E-05	0.000157065	4186865.912	3693671.356	1677410.147	2224425.076
299.16017	2.7944	4.93E-05	0.000152063	1295737.65	1316007.688	2431794.681	1617335.545
100.07619	2.047583	4.90E-05	0.000151197	2275640.015	2686633.053	1623081.201	4976567.316
325.20114	3.186875	4.87E-05	0.000150392	934188.7698	1041997.933	456870.9531	1192690.641
357.30012	4.765933	4.77E-05	0.000147492	436626.6358	583297.2834	163216.1183	421135.3773
339.18032	2.768167	4.47E-05	0.000138347	327259.3434	534730.4399	99408.38677	316822.6175
340.10273	2.463833	4.36E-05	0.00013491	313219.2381	255297.6138	1193643.584	392916.9518
132.10202	2.381167	4.34E-05	0.000134589	4224288.742	2991094.967	9188595.543	5652994.239
343.15005	2.516367	4.32E-05	0.000134079	1641141.375	1229337.619	903539.006	1117253.412
170.11758	2.454267	4.32E-05	0.000133897	11989193.86	9263805.859	7369347.465	13498157.54
157.02084	0.75852	4.26E-05	0.000132396	781129.7975	629239.9505	1430372.5	1332787.19
158.08118	2.061617	4.24E-05	0.000131708	2714619.404	1944427.348	3717947.022	2132732.502
279.23193	4.495483	4.11E-05	0.000127728	3424776.937	2607938.689	6068038.117	2547385.58
294.11243	2.497033	4.11E-05	0.000127728	649732.3313	447900.5032	773206.7322	1266473.658
270.01724	4.529958	4.04E-05	0.000125808	190577.7119	304453.6413	1143254.434	205674.4283
336.12324	3.081983	4.04E-05	0.000125808	3198857.374	1563432.939	909372.196	2496924.354
312.94298	2.9097	4.00E-05	0.000124622	2644714.424	3330924.697	4491372.728	3879634.784
228.57719	3.467	3.83E-05	0.000119615	341028.691	640785.3305	127561.0391	268848.2971
317.08099	3.134667	3.72E-05	0.000116224	1470548.843	2507011.803	363130.3528	830109.3103
330.33664	3.553242	3.71E-05	0.000116059	8510687.023	8247871.989	12765247.36	9733038.513
316.09689	2.724658	3.68E-05	0.00011501	653707.5369	164233.8838	612432.9211	850999.3063
198.05806	3.003692	3.60E-05	0.000112765	3151329.043	4326077.268	6622795.197	163369230.3
265.09721	2.52385	3.31E-05	0.000103738	918068.4208	1050682.424	4511558.969	722294.5591
210.08387	2.796192	3.25E-05	0.000102026	305277.8344	459456.2232	515910.9104	399993.1211
168.07324	2.497283	3.23E-05	0.000101218	946024.2738	585379.9051	1145974.194	2086716.563
146.06003	2.775808	3.12E-05	9.80E-05	543291.119	611790.2674	997592.436	686356.607
375.13982	2.445017	3.11E-05	9.76E-05	343975.6412	369458.6573	752523.8248	411756.182
239.07911	2.6025	3.07E-05	9.66E-05	523306.0706	710568.3357	779196.349	565106.7093
276.98671	0.737503	3.07E-05	9.66E-05	3145230.556	1323254.433	6883352.49	6839404.79
229.03191	1.9571	3.02E-05	9.50E-05	291688.193	233573.6571	999444.3668	542800.9612
306.11829	2.366067	3.00E-05	9.46E-05	1069562.596	706414.8047	2964699.68	1260094.675

Kruskal-Wallis significant metabolites positive ionization mode

472.20092	3.16765	2.99E-05	9.45E-05	191299.5867	835420.6271	120639.0115	129501.9764
192.97942	0.481082	2.95E-05	9.32E-05	588128.4293	475154.8455	1592149.165	956390.563
340.18674	2.4535	2.92E-05	9.22E-05	2669557.101	1789231.78	5322307.35	3116592.062
242.03352	1.983117	2.91E-05	9.19E-05	348060.7395	322455.673	701336.3657	362710.7192
153.05377	2.836975	2.87E-05	9.08E-05	981903.7108	731440.1507	667724.9109	1162027.093
252.17073	2.537083	2.85E-05	9.02E-05	1072241.57	980326.7927	1782147.924	1501274.523
359.10368	2.392567	2.81E-05	8.90E-05	798729.6556	588677.3502	586345.3941	562722.4466
365.19354	2.977133	2.81E-05	8.90E-05	3904522.621	3379724.891	827281.8961	5439755.415
190.10731	1.957267	2.80E-05	8.89E-05	235212.0384	191745.6065	471101.9288	455705.8419
196.00632	2.6111	2.79E-05	8.85E-05	1938431.166	2002489.248	1144707.064	1740938.712
186.07607	2.067417	2.78E-05	8.85E-05	884793.1278	691576.8616	505869.2053	640516.938
401.28737	4.129025	2.78E-05	8.83E-05	683699.1059	525042.7538	380565.0664	760776.0049
148.53629	0.459932	2.77E-05	8.83E-05	989863.662	1917310.264	830711.3325	1331843.283
379.25666	2.967983	2.77E-05	8.82E-05	440728.6705	282692.0067	225616.749	177582.6216
293.1131	2.199992	2.71E-05	8.64E-05	944323.3168	1162794.054	1756652.988	2924909.142
308.11852	2.558633	2.61E-05	8.33E-05	592027.7912	1115473.378	385558.2551	530274.7317
437.97273	0.748321	2.55E-05	8.15E-05	22257.64912	6191.398486	218239.761	161020.7526
307.11523	2.558833	2.52E-05	8.05E-05	3607218.473	6733547.949	1971207.189	1892054.728
373.12415	2.232758	2.37E-05	7.59E-05	183752.8802	119321.2477	464339.3075	190313.0149
146.08117	1.655943	2.36E-05	7.57E-05	870616.4749	1110296.804	1885745.087	2988051.895
546.40033	4.346942	2.32E-05	7.44E-05	1365891.683	1564021.579	663916.4343	2286660.44
237.14856	4.48545	2.31E-05	7.41E-05	417197.4125	772662.9451	138548.4867	323826.2796
353.2516	4.705275	2.26E-05	7.24E-05	682712.3433	486951.4273	351938.6282	447288.215
221.15364	2.85565	2.24E-05	7.19E-05	1173801.885	1112416.107	3284148.036	884000.8014
356.09176	2.485417	2.20E-05	7.08E-05	431573.9892	595255.3181	676573.9907	896464.719
265.11831	2.458017	2.19E-05	7.04E-05	550989.8854	647183.8113	1199357.708	540772.5881
407.18064	3.522633	2.17E-05	6.98E-05	454770.0736	1041197.643	154752.3148	356503.1766
438.98065	0.7473	2.10E-05	6.77E-05	52292.95609	11773.45988	555172.2932	386123.9858
193.00198	0.956672	2.09E-05	6.74E-05	1834393.723	2023975.15	4242712.385	3737424.25
418.25309	3.134583	2.01E-05	6.49E-05	163478.7332	178831.0124	3090.205468	308832.778
213.60126	3.162692	2.00E-05	6.46E-05	514298.9975	1932330.43	381861.6662	329332.8696
529.27736	2.994742	1.99E-05	6.43E-05	809255.5091	725386.1965	104295.3472	296674.728
335.18532	3.256558	1.97E-05	6.37E-05	3293156.54	6797148.425	828845.3452	3861640.568
433.26968	3.59835	1.92E-05	6.20E-05	446010.9645	612724.7111	39651.2288	587411.7901

Kruskal-Wallis significant metabolites positive ionization mode

203.07027	2.829533	1.87E-05	6.06E-05	2313374.868	3412832.615	4550637.238	1952038.477
202.98621	0.957835	1.85E-05	6.01E-05	506805.6888	509082.2734	1035413.805	824472.4246
387.17627	2.784092	1.83E-05	5.93E-05	9046044.271	24140253.08	1066337.863	4963887.56
198.0831	3.0821	1.82E-05	5.90E-05	15436215.8	12967423.68	2043820.054	5043069.895
102.53598	3.245933	1.81E-05	5.88E-05	8149211.404	11259396.05	10813516.48	7202495.3
243.06526	2.722642	1.81E-05	5.87E-05	1532839.479	2607085.298	601313.8766	1038638.647
329.17238	3.27375	1.79E-05	5.83E-05	8929853.777	11905372.77	1437327.599	7281586.043
394.31867	5.358858	1.77E-05	5.78E-05	28375575.52	33561592.64	12589226.9	28562689.24
273.14854	3.16385	1.75E-05	5.71E-05	3261312.413	3611205.513	7111230.865	1740123.127
185.12849	2.363467	1.75E-05	5.70E-05	908622.9594	976962.5158	1342604.52	2389897.906
401.17262	3.518542	1.73E-05	5.63E-05	1307124.54	1387053.39	134660.7523	725307.8985
242.98249	0.755312	1.72E-05	5.61E-05	340600.8058	236440.9193	817672.988	769898.431
352.08968	2.9419	1.72E-05	5.61E-05	1575381.092	1469857.895	319020.2269	1058473.495
384.11493	2.054258	1.71E-05	5.58E-05	339223.4541	230966.8648	735837.8504	324794.9732
331.21803	3.160975	1.69E-05	5.54E-05	7029939.197	11242064.61	2102139.927	7093476.641
352.21752	2.994333	1.69E-05	5.54E-05	1228754.351	696670.343	338015.3068	768941.3397
327.23193	3.815842	1.67E-05	5.49E-05	592276.5104	862154.1	181757.742	369376.1665
295.22681	4.216317	1.65E-05	5.40E-05	3044196.635	2837603.603	1373048.581	4828586.85
333.24239	4.422033	1.63E-05	5.35E-05	1383204.295	2294780.743	380730.9501	1721438.176
747.48087	3.789158	1.57E-05	5.16E-05	132039.2569	330466.4133	5318.980424	81992.61476
398.26687	3.570733	1.57E-05	5.15E-05	636011.9168	334269.986	216835.9486	272556.6168
230.98125	0.748458	1.57E-05	5.15E-05	536111.1665	441479.4899	1089216.706	1134463.105
379.24795	2.911975	1.53E-05	5.04E-05	5308944.714	7147031.025	809546.5186	2676978.582
563.18173	4.382017	1.53E-05	5.04E-05	58047.71447	29980.21355	1615.130732	64461.60032
590.42651	4.338383	1.49E-05	4.93E-05	1352690.64	1484349.584	646769.6724	2185244.783
233.09552	3.562267	1.46E-05	4.83E-05	2882648.671	6162836.795	10366787.3	2626642.855
503.37751	4.347417	1.43E-05	4.72E-05	253198.7015	354544.9475	71473.04498	459062.8508
202.58391	2.407192	1.42E-05	4.70E-05	319432.413	483406.8723	727468.183	955352.6633
395.32198	5.367817	1.40E-05	4.64E-05	3657380.908	4357040.546	1510291.933	3647069.602
128.10716	2.485383	1.39E-05	4.62E-05	2837424.785	1386254.279	2977209.651	5840185.777
160.09685	2.052808	1.39E-05	4.60E-05	9413670.047	11369646.01	7415813.326	22577368.47
154.51809	0.753982	1.35E-05	4.47E-05	699640.6037	354186.5471	1692437.821	1555848.939
568.03609	2.672892	1.32E-05	4.37E-05	1063954.633	952145.0311	67308.00961	640671.8186
292.95888	0.687554	1.31E-05	4.35E-05	573951.8866	341336.2674	1527248.253	1240864.246

Kruskal-Wallis significant metabolites positive ionization mode

360.16536	2.554267	1.30E-05	4.34E-05	525959.9896	148845.2627	327766.6937	582589.8894
189.01378	0.46312	1.30E-05	4.32E-05	597465.7593	884634.1108	1255847.887	742607.8925
591.4299	4.3317	1.25E-05	4.18E-05	281213.0326	340013.0756	77186.96273	520435.5668
163.07535	3.0866	1.25E-05	4.17E-05	704614.6558	376632.2575	469194.7216	610483.9081
177.5341	0.75956	1.22E-05	4.09E-05	260989.5251	119511.4722	806704.4684	721605.1952
317.14961	2.968617	1.22E-05	4.06E-05	856157.9834	487177.3877	313763.3116	492505.7679
313.21621	3.913483	1.21E-05	4.03E-05	3223037.918	5959997.685	1078426.883	2577529.807
397.25863	3.125617	1.19E-05	3.97E-05	3853159.294	8433049.32	842788.6769	2407057.805
303.11863	2.3177	1.19E-05	3.97E-05	292473.673	200074.6978	527795.1763	311789.3199
409.19635	3.1485	1.16E-05	3.88E-05	348734.3742	601516.6656	79261.47772	296942.0732
743.44932	3.728533	1.13E-05	3.80E-05	244693.1332	892562.3679	6567.757686	126091.3516
423.0081	0.750772	1.09E-05	3.66E-05	113308.429	15265.30271	1143587.584	951878.3866
230.13875	2.411417	1.09E-05	3.65E-05	2805962.202	1368663.05	1414924.174	1896721.836
413.28739	4.723433	1.09E-05	3.65E-05	511799.6488	489773.9987	240163.9881	455869.4692
234.10339	4.3559	1.08E-05	3.62E-05	982962.2328	1788622.532	349064.1849	817016.4289
375.25307	3.7627	1.07E-05	3.60E-05	818716.093	3118782.438	118516.2144	717518.9918
416.24316	3.9474	1.03E-05	3.46E-05	294171.4084	216107.0306	28665.92226	236578.7748
104.10738	3.431533	1.02E-05	3.44E-05	336861.761	357581.8835	1186498.195	356314.5899
147.1168	3.16745	1.02E-05	3.44E-05	599416.5056	1064751.618	1252131.963	536554.9735
242.09983	2.06225	1.01E-05	3.42E-05	493070.072	393260.7635	1254438.917	507937.8117
148.05749	2.377417	1.00E-05	3.39E-05	434351.2146	405379.6788	944305.6867	583222.8647
145.51283	0.755853	9.94E-06	3.36E-05	680094.2423	319401.51	1768074.655	1710673.594
346.16499	3.003467	9.74E-06	3.30E-05	1087099.418	1662962.929	270105.3416	703022.2673
315.13383	2.402733	9.47E-06	3.21E-05	1057805.895	497324.7432	2877074.149	451366.6092
263.16426	3.826933	9.38E-06	3.18E-05	725742.9633	949334.2506	427031.1718	572605.4315
222.0761	2.39785	9.35E-06	3.17E-05	437417.4771	403449.3482	835645.178	429650.7157
256.56012	2.933908	9.24E-06	3.14E-05	362449.8242	400903.5695	48465.96315	260181.7063
367.22451	4.445567	9.07E-06	3.08E-05	14152961.39	45649502.93	572694.6528	11684652.9
261.15981	2.829667	8.73E-06	2.97E-05	132303.1149	177803.29	116516.0858	774957.9557
200.20089	5.7954	8.71E-06	2.97E-05	20997836.95	24951564.45	14643026.01	15374345.82
352.09548	2.551383	8.16E-06	2.78E-05	860806.3737	489891.7488	664323.2165	1270577.728
276.07146	2.008333	8.10E-06	2.76E-05	318122.7652	285202.8932	655582.5182	362557.9202
343.20573	2.502183	7.99E-06	2.73E-05	343733.2666	292233.1662	836895.052	501272.6612
215.01617	0.735565	7.85E-06	2.68E-05	2692076.139	1045719.112	8843967.117	7972498.836

Kruskal-Wallis significant metabolites positive ionization mode

299.0914	2.06185	7.84E-06	2.68E-05	666036.2702	271191.3815	816051.2603	632696.586
331.11349	2.290567	7.55E-06	2.58E-05	84313.91597	52933.26018	372498.8849	124185.2923
351.08635	2.942908	7.55E-06	2.58E-05	8245635.604	7949743.974	1409543.143	5457657.024
458.19221	3.04415	7.53E-06	2.58E-05	301726.97	432364.5155	1419079.48	201813.574
210.57587	2.50875	7.41E-06	2.54E-05	182681.0347	112705.1461	476724.3337	167870.8622
154.04988	2.113517	7.22E-06	2.48E-05	1249815.83	1275418.994	4364944.877	1658134.254
125.07113	2.033033	7.21E-06	2.48E-05	1053063.825	951369.4513	1677573.321	935534.0161
345.24247	3.740592	7.00E-06	2.41E-05	6230941.193	6362762.987	957302.8831	3005899.283
552.29836	4.198867	6.88E-06	2.37E-05	250801.8316	292638.6194	519737.9763	123953.9858
331.06013	3.431167	6.85E-06	2.36E-05	1866696.889	1841777.506	161274.8576	1452044.93
238.96737	0.749947	6.70E-06	2.31E-05	140286.4934	117632.6647	656193.4213	454134.3043
314.21956	3.911017	6.14E-06	2.12E-05	564162.4291	964942.4267	108238.2808	485565.7966
367.26323	3.389783	6.10E-06	2.11E-05	559350.741	1072158.357	18024.80976	383825.3073
210.06082	0.726501	6.02E-06	2.08E-05	998558.1257	425145.6019	2807936.595	2912910.144
168.01614	2.933692	5.96E-06	2.06E-05	3968450.634	5159538.714	5564727.593	4979558.064
213.1597	2.63725	5.96E-06	2.06E-05	253010.4777	357106.8993	325017.8642	825204.6328
201.16382	4.386808	5.93E-06	2.05E-05	6152531.333	4100527.896	878525.0133	6686434.384
289.98041	0.747641	5.88E-06	2.04E-05	303801.5852	117047.3968	810224.3636	811257.9715
441.2388	3.134833	5.83E-06	2.02E-05	184898.6782	316448.141	13540.88447	145132.0342
423.28531	2.8014	5.76E-06	2.00E-05	392468.02	674891.2238	46239.28068	277653.6478
282.13366	2.6344	5.74E-06	1.99E-05	1787710.072	547707.4284	2627923.397	1147304.231
332.23012	3.518542	5.60E-06	1.95E-05	7198256.802	9989601.891	424468.3915	4099151.767
744.45274	3.731917	5.51E-06	1.92E-05	142291.3917	397595.2948	4986.88463	65415.99187
634.45272	4.329658	5.46E-06	1.90E-05	1111066.594	1359994.653	381981.8018	1872334.121
221.58751	2.783917	5.17E-06	1.80E-05	260650.7685	624211.9491	78203.4313	162851.3701
173.1285	1.983117	5.16E-06	1.80E-05	1094048.183	1012561.09	921114.9229	3341839.382
463.17782	3.803858	5.05E-06	1.76E-05	475757.2081	721998.5901	36429.26083	599116.1895
211.14423	2.523792	4.93E-06	1.72E-05	109379575	100452147.3	180092366.6	139640355.6
191.17939	3.829233	4.92E-06	1.72E-05	1233879.677	2000571.456	357514.2101	860050.651
349.23742	4.861742	4.91E-06	1.72E-05	395066.8977	647024.1022	51776.17809	298970.8351
473.26479	3.6306	4.76E-06	1.67E-05	717508.1167	612348.5786	1451199.298	785703.9235
360.02367	0.762105	4.61E-06	1.62E-05	80473.42759	38066.5931	392699.2871	595959.1328
305.07675	2.22945	4.60E-06	1.61E-05	594186.994	347240.249	1755055.111	667436.7454
356.21802	2.639383	4.60E-06	1.61E-05	522338.5648	472555.4553	1140397.753	868851.0085

Kruskal-Wallis significant metabolites positive ionization mode

332.1433	4.495617	4.53E-06	1.59E-05	146603.1659	57551.8757	582277.1889	189396.3027
126.03896	1.9116	4.51E-06	1.59E-05	1841781.186	47203825.73	2952090.62	2608845.607
221.15365	3.22785	4.49E-06	1.58E-05	975482.167	1022835.333	4374526.097	888196.1242
220.118	2.063733	4.47E-06	1.58E-05	4620429.047	3680602.582	12478558.35	5813769.679
272.00771	0.75625	4.44E-06	1.57E-05	1243880.596	450841.3782	3234924.546	3179356.67
287.97999	0.747829	4.35E-06	1.54E-05	3982482.094	1483753.223	10236679.26	9223788.881
197.0809	3.553133	4.22E-06	1.49E-05	4717894.721	14390962.04	1247763.435	3113102.085
574.33762	3.6777	4.20E-06	1.49E-05	46139.85758	105186.5386	701311.461	142266.4996
288.98359	0.753175	4.17E-06	1.48E-05	294333.6503	159183.7881	690631.2094	685724.8558
234.11466	3.16935	4.14E-06	1.47E-05	3580940.288	11668118.44	4919989.418	1998281.191
678.47891	4.320842	4.11E-06	1.46E-05	847568.4097	1068577.389	278505.6049	1448334.939
403.24554	3.362558	4.08E-06	1.45E-05	5345232.125	7447820.251	735907.5674	3229025.003
353.22982	3.082033	4.01E-06	1.42E-05	1767409.913	1179001.345	593506.5519	3120031.793
129.10239	2.361867	3.96E-06	1.41E-05	675158.4086	595365.9497	1945894.577	966377.7658
244.19083	3.056733	3.95E-06	1.41E-05	1014315.118	1598566.689	1711735.103	1216470.745
290.01819	0.754757	3.93E-06	1.40E-05	299904.1955	116412.5451	824029.2043	736460.5139
166.08631	2.49355	3.87E-06	1.38E-05	1382938.589	1502406.342	3009499.563	1945126.113
128.10716	2.0533	3.69E-06	1.32E-05	1919914.73	1699607.083	6393970.615	4501361.007
376.25633	4.175075	3.68E-06	1.31E-05	189630.4874	1171826.16	18859.25903	234801.5854
331.22679	3.509633	3.60E-06	1.29E-05	29449257.93	49533005.11	4441787.325	13574773.72
325.20105	2.920992	3.56E-06	1.27E-05	1990832.805	3408132.964	968349.182	2567019.306
409.17504	3.584283	3.08E-06	1.10E-05	394633.1883	452936.3494	184050.8707	85875.65555
214.13365	2.650092	3.00E-06	1.08E-05	1557627.92	885042.2533	926058.3289	1378053.456
553.06723	2.672683	2.98E-06	1.07E-05	376722.847	359073.925	26875.74011	189050.7791
766.53125	4.30305	2.94E-06	1.05E-05	316058.6929	446952.4059	56947.73817	577373.083
331.06014	3.8322	2.93E-06	1.05E-05	2810501.648	3190689.735	749365.0371	1729580.556
365.27719	3.33025	2.89E-06	1.04E-05	770546.3636	715149.4805	153501.9984	695497.1251
205.15467	2.045475	2.85E-06	1.03E-05	320919.8147	382273.1428	202879.0528	570637.0562
164.02862	1.963217	2.78E-06	1.00E-05	282847.7946	240955.3846	748955.3035	441729.7616
296.23015	4.216617	2.72E-06	9.79E-06	336781.8701	381837.8206	126508.6261	623630.8339
323.07609	2.75795	2.68E-06	9.67E-06	810524.776	577482.4173	256796.0096	351439.3759
333.23386	3.5197	2.55E-06	9.19E-06	1454670.872	1369870.415	410392.2842	1237195.581
325.21634	3.735308	2.51E-06	9.08E-06	1059542.3	1584858.098	285138.0341	679088.9109
593.09027	2.6725	2.49E-06	9.01E-06	542222.7138	500532.256	29291.37639	317522.3511

Kruskal-Wallis significant metabolites positive ionization mode

322.10741	2.311875	2.38E-06	8.60E-06	1452452.85	1830580.107	2352905.05	2907575.233
221.1173	3.911133	2.36E-06	8.55E-06	602497.8162	901315.8693	1774069.159	404293.3998
513.28253	3.2831	2.34E-06	8.50E-06	2052386.3	1899514.746	306940.6423	1229354.016
679.48233	4.316917	2.34E-06	8.50E-06	197759.4975	302945.6075	27455.43296	347110.2811
168.0995	2.053083	2.32E-06	8.44E-06	811740.6938	711095.9011	2920001.072	1828778.464
344.31614	4.517467	2.28E-06	8.30E-06	802423.5778	815512.5099	342917.1921	697992.6801
167.01285	0.041394	2.21E-06	8.05E-06	126867.1352	3570483.267	22090462.58	3932313.52
389.23211	3.527133	2.08E-06	7.58E-06	2452292.905	4657346.145	391874.6475	1727537.754
374.17111	2.625117	2.06E-06	7.50E-06	381195.1501	376166.3147	650157.5756	336221.9252
419.17613	3.064917	2.00E-06	7.30E-06	6849133.29	6681668.231	457561.4457	3805971.536
241.10419	3.10975	1.99E-06	7.28E-06	660600.448	2797668.951	515131.5802	378597.471
336.18864	3.256975	1.98E-06	7.23E-06	486716.923	1070175.762	108629.0481	826498.3418
520.10822	4.495483	1.97E-06	7.21E-06	297958.2807	141233.6765	1581023.322	285265.4643
444.18344	3.63155	1.95E-06	7.15E-06	666145.4256	1327152.656	34230.38354	771215.2037
111.04434	1.974525	1.89E-06	6.93E-06	5365420.317	3421833.983	14695358.88	2391780.993
467.17292	3.227783	1.88E-06	6.90E-06	448919.2016	1129004.665	41517.97107	316534.3186
343.19059	3.117075	1.82E-06	6.66E-06	4280445.935	7292016.476	717863.991	3095705.783
263.10264	2.343833	1.80E-06	6.62E-06	1943578.928	1449736.226	5100764.598	2152812.866
445.33151	4.303533	1.78E-06	6.56E-06	272618.674	1413427.798	34873.52355	422603.7755
223.98847	5.961033	1.75E-06	6.43E-06	22517206.42	36500341.52	70453969.93	26038259.99
170.56263	2.062083	1.71E-06	6.32E-06	429885.3503	361376.675	1161783.082	556744.1611
342.20243	2.506367	1.66E-06	6.14E-06	1786619.82	1622802.125	5284177.86	3089123.583
223.98846	4.32085	1.65E-06	6.10E-06	55758080.79	79939090.28	122764849.1	70861239.62
140.06818	3.549083	1.65E-06	6.09E-06	12770890.03	7989906.041	5385150.569	10680310.49
279.08642	2.490817	1.65E-06	6.09E-06	2073743.629	1815019.985	1405995.707	898618.6725
387.21419	2.797117	1.60E-06	5.93E-06	35804806.06	52139618.49	3830988.795	20718309.86
251.20059	3.290325	1.59E-06	5.89E-06	1329363.336	1219356.288	719408.3605	1356385.765
291.00223	1.956942	1.58E-06	5.85E-06	100871.3583	89736.84443	671998.9757	318785.1564
147.1209	2.052875	1.56E-06	5.80E-06	451501.8666	465254.5517	2046565.947	1347717.179
225.07227	3.108217	1.55E-06	5.75E-06	1893387.713	2548534.681	828859.9171	1776733.739
162.05903	2.8292	1.46E-06	5.43E-06	763831.2491	375213.6656	365073.9289	833352.6239
393.3152	5.1847	1.45E-06	5.39E-06	18298772.87	20733839.85	6743288.361	12890092.57
362.24065	3.822667	1.45E-06	5.38E-06	12386974.15	18857791.1	560398.6942	6396059.711
301.9957	1.95635	1.44E-06	5.37E-06	207136.3177	173381.7857	934634.809	381804.5056

Kruskal-Wallis significant metabolites positive ionization mode

407.24039	2.925767	1.42E-06	5.31E-06	1098834.836	1099013.324	393286.1098	527130.3992
268.61407	4.382067	1.42E-06	5.29E-06	248520.2995	75193.29404	2430.191155	288666.1397
127.03915	2.528508	1.40E-06	5.25E-06	3987778.554	3839617.59	2187591.59	2210449.069
394.23067	3.407267	1.40E-06	5.24E-06	699636.4873	1161341.6	99564.66398	361925.0782
277.11838	2.485	1.40E-06	5.23E-06	4981180.078	5908232.067	9951716.83	16998526.66
380.05887	3.535683	1.37E-06	5.14E-06	879590.8725	199116.9594	85100.68344	139490.7533
348.05839	3.5359	1.31E-06	4.90E-06	1558602.81	983289.3987	130035.8608	505056.5691
146.11762	2.054408	1.29E-06	4.84E-06	7045200.125	8237008.966	33737263.95	23990012.83
353.19604	3.117033	1.27E-06	4.77E-06	1136605.285	1491037.64	296525.7952	1345665.72
177.007	0.482612	1.27E-06	4.77E-06	1100566.417	1132385.984	2785711.203	1992038.025
368.22783	4.486317	1.26E-06	4.75E-06	3192612.552	10443504.23	56046.18731	3233984.072
458.40338	3.60375	1.25E-06	4.69E-06	285757.6168	421812.7988	58447.17392	117240.5406
485.04321	3.431583	1.18E-06	4.45E-06	488081.9092	448863.6667	9717.203004	376300.3257
319.15166	2.93385	1.18E-06	4.45E-06	1542986.123	2099219.366	485703.7216	1287963.227
382.23063	3.160617	1.17E-06	4.43E-06	469503.3064	1082654.182	73361.07902	262092.1106
166.08629	2.837225	1.11E-06	4.20E-06	1971214.522	1010485.967	1043616.723	1774318.394
297.04882	2.897992	1.10E-06	4.17E-06	1326207.942	2569804.147	2663794.413	2014490.344
365.102	2.977217	1.10E-06	4.16E-06	2705584.77	2895737.855	561693.1672	1538158.688
227.17541	2.810083	1.08E-06	4.10E-06	191405.3232	229232.0336	197874.4921	706414.5766
405.26121	3.33025	1.06E-06	4.01E-06	4659022.953	4317221.864	724011.1799	4647601.438
219.06039	2.506583	1.04E-06	3.95E-06	687835.5122	705434.0222	562413.4274	285789.1394
301.14772	3.108125	1.00E-06	3.80E-06	969723.4614	416466.1232	614871.0351	808448.9168
266.17266	3.056033	9.78E-07	3.72E-06	940164.6229	429903.8158	339854.2558	797781.5802
388.21753	2.801783	9.46E-07	3.60E-06	7775231.572	11634252.49	978208.2565	4427532.388
237.07551	2.502733	8.94E-07	3.41E-06	969797.2224	663859.378	1929409.456	390691.1053
416.23752	3.1169	8.87E-07	3.38E-06	801980.728	1200983.482	56482.99399	652364.5876
384.24611	2.769567	8.63E-07	3.30E-06	961616.1925	1814604.794	248145.453	576081.0261
221.03457	2.298775	8.53E-07	3.26E-06	242910.1323	71766.99697	68986.6234	389563.1262
187.14814	3.169467	8.38E-07	3.21E-06	915208.5069	1501843.576	349790.2406	674596.7256
363.25293	3.359833	8.36E-07	3.20E-06	4988163.139	10110836.8	201074.4299	2874689.337
389.23224	4.051233	8.04E-07	3.08E-06	919274.6023	3242639.516	45037.78491	928103.1657
221.12131	2.0623	7.81E-07	3.00E-06	416031.5895	345310.6242	986745.0145	472520.6993
443.15176	4.408275	7.78E-07	2.99E-06	290489.1246	417275.5587	12370.74761	239602.5814
496.1977	4.382067	7.29E-07	2.80E-06	187417.9156	77646.36944	1793.784192	223346.4511

Kruskal-Wallis significant metabolites positive ionization mode

722.50503	4.311833	6.98E-07	2.69E-06	571375.375	741333.0403	106779.9938	945140.6395
259.06018	2.54325	6.82E-07	2.62E-06	1735628.06	1374919.073	926216.4341	693603.8575
357.1656	2.6672	6.58E-07	2.54E-06	721248.9998	660777.567	1632419.858	928502.7097
323.02845	0.504262	5.54E-07	2.14E-06	456292.7545	507339.1108	1676964.874	712912.8709
325.16461	2.93325	5.37E-07	2.07E-06	2411593.673	3137868.464	937583.3208	2692806.55
551.28284	2.839633	5.30E-07	2.05E-06	153464.3195	158215.0003	4795.326471	71061.45506
144.04784	1.911283	5.19E-07	2.01E-06	14870959.51	19803656.55	45791906.86	17754058.26
287.09138	3.674992	5.14E-07	1.99E-06	703917.1589	194783.0052	106361.3487	165666.3478
124.03952	0.671957	5.14E-07	1.99E-06	1813185.9	1643962.548	5696664.974	4018108.708
213.00701	1.958183	4.62E-07	1.79E-06	506898.6727	507488.82	1332613.591	512980.3384
449.18479	2.792633	4.55E-07	1.77E-06	875736.2631	1855464.611	123720.7366	609159.0789
380.25133	3.720467	4.40E-07	1.71E-06	1389874.489	3402644.126	488820.3667	1090045.806
177.05463	2.5447	4.37E-07	1.70E-06	478797.2229	431601.8658	1308839.995	446137.4871
347.05512	3.535817	4.37E-07	1.70E-06	9120146.719	6654258.396	1348218.9	3428053.358
518.32433	4.146833	4.37E-07	1.70E-06	584053.0049	663837.4941	2452976.414	366428.3705
355.33195	4.439317	4.21E-07	1.64E-06	905702.4902	559246.6922	450744.6766	904676.4432
404.24893	3.3642	4.16E-07	1.63E-06	1100216.26	1771288.828	160956.0467	675539.9233
457.40005	3.604858	4.13E-07	1.61E-06	1352502.977	1833335.769	292805.9962	679685.2489
182.98524	0.058838	4.12E-07	1.61E-06	75679642.79	587209.5511	37231.46899	116103071.7
544.36343	3.68395	4.09E-07	1.60E-06	168901.2238	443917.4205	1340609.802	500660.8713
223.13283	3.330983	4.02E-07	1.58E-06	3960947.101	3466185.676	1340181.809	2970692.485
349.22851	3.169233	4.02E-07	1.58E-06	4925611.462	7605368.756	1521358.518	3840174.957
395.33099	3.745208	3.86E-07	1.52E-06	282451.8364	287462.2216	1029754.494	413466.7803
398.23265	4.01575	3.81E-07	1.50E-06	1512182.47	1237940.583	131634.9094	1485539.641
328.14191	3.195683	3.52E-07	1.39E-06	733618.0842	1440657.08	2326534.173	843448.2806
386.23262	4.216267	3.40E-07	1.34E-06	392786.814	340164.5713	20986.19962	310527.9569
186.58908	2.514892	3.39E-07	1.34E-06	1314583.722	1427841.809	2655334.267	1647385.608
199.08582	3.064767	3.38E-07	1.34E-06	34903923.55	2702129.171	664152.2338	12967659.1
303.19549	3.888525	3.38E-07	1.34E-06	691402.7634	892724.1294	123038.6988	537847.913
298.09679	2.133467	3.37E-07	1.33E-06	699856.571	578983.7185	1904086.378	1112948.729
321.20611	3.30825	3.32E-07	1.31E-06	949490.215	1079026.121	282249.5641	1070393.009
437.32225	3.59735	3.32E-07	1.31E-06	109536.4343	227149.2533	593771.7524	150812.724
441.22489	3.562033	3.25E-07	1.29E-06	518743.5434	598169.8782	48069.00808	340940.2824
209.19002	3.64025	3.08E-07	1.22E-06	770793.5377	967283.4044	488935.4205	732608.3484

Kruskal-Wallis significant metabolites positive ionization mode

255.17038	2.537983	2.98E-07	1.18E-06	486414.5838	402564.6375	899352.9413	354320.2072
167.07028	2.8399	2.88E-07	1.14E-06	2160648.356	877333.1694	1340842.89	1419154.315
228.60665	3.343742	2.85E-07	1.13E-06	695648.2656	841215.9283	96230.46095	599750.5283
380.25132	3.533433	2.67E-07	1.06E-06	1949241.845	4128151.985	338115.6593	1255507.034
186.11253	2.556083	2.66E-07	1.06E-06	1187656.779	770241.8758	922022.3192	2138148.104
190.06809	2.427133	2.59E-07	1.03E-06	1126628.314	344476.9234	587553.5853	962088.6803
315.06512	3.5358	2.43E-07	9.73E-07	1367051.745	1332602.061	454516.8343	377203.2443
549.26719	2.803925	2.34E-07	9.37E-07	525714.4183	635099.3218	17277.87279	259218.791
289.17986	3.152392	2.20E-07	8.81E-07	1490086.969	2246976.618	327877.9535	1790025.307
264.08679	2.645767	2.18E-07	8.75E-07	255451.7219	277675.5886	820911.3008	403608.4007
818.41674	4.415925	2.17E-07	8.70E-07	198285.4675	3880.474833	2803.135279	239134.8612
335.08878	3.082233	2.14E-07	8.60E-07	19981592.85	5114627.725	4296586.073	16259240.33
139.08663	2.12285	2.06E-07	8.29E-07	1425129.974	1091892.45	5168541.497	1273397.622
361.23732	4.491458	1.98E-07	7.96E-07	2025428.508	5010587.885	57245.36312	1471430.189
450.16568	3.630458	1.89E-07	7.59E-07	347645.8147	641167.4893	16589.21041	387420.9698
467.19541	2.776533	1.82E-07	7.33E-07	535317.2975	1229902.967	52954.18029	451322.5986
120.08106	2.581033	1.81E-07	7.29E-07	1439413.018	2036526.531	3273157.401	3733267.937
454.35096	3.381483	1.78E-07	7.20E-07	56743.08544	22192.16537	1441527.249	50322.28523
552.06373	2.675433	1.73E-07	7.01E-07	1474374.595	1397746.272	100746.8206	947075.4333
378.32366	3.745617	1.66E-07	6.73E-07	326407.1174	591658.554	2610486.91	559521.1946
398.20199	3.9259	1.61E-07	6.54E-07	2126145.029	3110751.71	176931.4525	1210558.129
345.20614	3.710658	1.60E-07	6.50E-07	7110603.856	9712639.251	781362.8348	4959662.631
412.21755	4.043133	1.60E-07	6.48E-07	226569.5221	1074671.343	7740.342067	193962.4889
453.34758	3.380467	1.57E-07	6.38E-07	786865.3138	438180.0755	6696189.337	573525.4794
449.2411	4.4869	1.52E-07	6.18E-07	146808.7711	387967.1668	5146.363144	107526.6659
188.97061	0.550102	1.49E-07	6.07E-07	418887.2764	538101.9571	1409575.428	514682.1772
381.26366	3.562183	1.45E-07	5.90E-07	1366653.782	1555830.941	103472.5709	918109.357
243.11993	2.794317	1.38E-07	5.64E-07	3414158.591	7221324.415	709514.7687	1881412.661
209.19002	3.231642	1.33E-07	5.44E-07	626759.7416	813590.34	344769.7895	573431.4211
211.06015	2.480167	1.33E-07	5.43E-07	6082650.017	6788257.66	45038023.6	69758382.45
141.05462	2.351683	1.30E-07	5.32E-07	1120572.407	1059533.776	4973505.541	1232184.474
507.29558	3.63145	1.27E-07	5.21E-07	180793.2728	480341.4374	15207.80865	149270.7221
455.07956	2.675633	1.27E-07	5.19E-07	3384469.723	2765062.757	197823.3759	2259499.878
196.06815	2.584858	1.21E-07	4.97E-07	1386273.395	653366.792	880445.8475	1330578.041

Kruskal-Wallis significant metabolites positive ionization mode

367.2391	3.108508	1.17E-07	4.80E-07	2062732.163	2985717.862	531254.3848	1886150.678
449.16224	3.631867	1.13E-07	4.63E-07	1849604.205	3335721.698	94969.96838	1798448.11
497.19422	4.381983	1.12E-07	4.61E-07	141508.1203	63163.47715	1394.238047	144047.9218
185.07845	2.098367	1.12E-07	4.60E-07	547992.7512	467999.3663	207847.4864	370550.5807
343.15184	3.117067	1.10E-07	4.55E-07	8791078.476	11304241.35	846485.295	7589683.37
289.1228	2.836317	1.10E-07	4.55E-07	740724.3005	283319.8904	291099.413	373613.8561
547.28795	3.187183	1.10E-07	4.55E-07	742982.1391	918827.6107	43540.17197	1025180.92
377.32033	3.749042	1.06E-07	4.39E-07	1422331.904	2489567.338	9653303.035	2193805.695
344.15513	3.118275	1.05E-07	4.36E-07	2164315.694	2962367.204	229165.1017	1693276.76
343.22696	4.347592	1.05E-07	4.35E-07	9210282.703	21504774.4	777873.0787	3719613.545
395.24293	2.787308	1.01E-07	4.17E-07	2576182.063	4293973.308	531260.6823	1667353.921
344.2303	4.351867	9.84E-08	4.08E-07	1643337.664	4664655.861	107017.755	1019443.856
461.16217	3.731942	9.38E-08	3.89E-07	577058.2948	1059130.761	26469.00765	372302.1281
404.21263	3.169233	8.74E-08	3.63E-07	1395663.259	1998318.953	128982.2383	787636.929
222.04318	2.829133	8.50E-08	3.54E-07	521392.2002	326103.1554	198045.5342	383554.8909
265.14355	3.021342	8.50E-08	3.54E-07	418386.8669	546086.915	2682056.585	345313.4225
382.2669	3.365017	8.36E-08	3.49E-07	375453.7073	688868.3865	10334.05331	254409.5646
564.2862	2.827767	8.23E-08	3.44E-07	280793.4786	367640.2992	3908.117492	132910.3893
376.21999	3.92835	8.08E-08	3.38E-07	543948.1156	1717944.734	60374.50804	388610.7204
194.05788	2.24665	8.06E-08	3.37E-07	939974.9114	585400.3085	350915.4383	549811.0629
383.24283	2.775733	6.98E-08	2.92E-07	5119946.005	9666683.133	903712.2038	2420494.025
415.24552	3.7637	6.69E-08	2.80E-07	637199.4728	1151790.665	34735.00759	523552.6603
224.01171	3.012892	6.67E-08	2.80E-07	5040168.937	6176917.557	9343666.001	5929623.336
386.23826	3.7924	6.58E-08	2.76E-07	4615495.079	12094378.37	154368.7863	3897457.504
511.36148	3.745608	6.52E-08	2.74E-07	341823.3802	760076.0624	3224778.589	755831.0958
376.11805	2.537433	6.50E-08	2.73E-07	672201.9195	708083.969	94107.09724	392735.2362
403.18821	2.801267	6.48E-08	2.73E-07	3029453.511	3672326.492	357991.9252	2061333.607
518.34546	3.902275	6.47E-08	2.73E-07	671735.603	564161.9349	3086726.701	436821.1764
327.23191	4.486733	6.39E-08	2.70E-07	1731336.62	4604859.265	79672.45532	1275326.212
403.15678	3.910958	6.36E-08	2.69E-07	1047545.873	1032481.911	421495.3266	1061266.034
381.06052	2.75265	6.34E-08	2.68E-07	604034.1232	527776.2974	100505.9327	237122.2284
406.26455	3.332617	6.28E-08	2.66E-07	992490.5661	929738.6932	99964.89507	1030027.462
347.24933	4.485617	6.15E-08	2.61E-07	8822528.84	20161004.01	261586.1097	6136648.232
418.22812	3.379	6.06E-08	2.57E-07	822317.4909	1521420.821	281625.1698	486888.0683

Kruskal-Wallis significant metabolites positive ionization mode

243.62159	2.794317	6.03E-08	2.56E-07	714848.6818	1623192.34	74410.89973	410095.6099
159.11684	3.593842	5.98E-08	2.54E-07	496776.2171	921346.7635	1593030.61	450966.562
174.99141	0.958115	5.94E-08	2.53E-07	2516397.654	3541181.035	10930722.71	6280533.89
361.23733	3.805233	5.93E-08	2.53E-07	53837142.11	109009148.6	2483283.574	29520319.61
333.23372	4.36445	5.89E-08	2.51E-07	2733285.147	4876770.779	375000.3194	2977765.965
560.35839	3.6731	5.59E-08	2.39E-07	77484.23328	153976.4865	783472.4697	311095.3445
519.34886	3.904683	5.35E-08	2.29E-07	154355.5101	140080.0326	1036320.443	118572.6469
191.1794	4.481067	5.03E-08	2.15E-07	1037766.997	2664969.506	107338.3097	586360.3229
391.22263	3.5359	4.87E-08	2.08E-07	737283.1863	551911.0348	140654.2411	617840.5365
257.13834	2.507675	4.73E-08	2.03E-07	1207040.031	1666027.655	368154.298	966527.5701
254.61661	4.356467	4.71E-08	2.02E-07	436826.1458	1211478.616	9776.723886	485263.4695
290.11688	2.8371	4.61E-08	1.98E-07	791511.6529	226972.848	186064.5992	499578.7224
529.27743	3.628267	4.53E-08	1.95E-07	552441.0906	1084624.974	42963.87505	381528.0446
455.22652	2.916425	4.50E-08	1.94E-07	342118.9648	161727.8505	17301.84518	241485.3678
250.09826	3.5179	4.36E-08	1.88E-07	219063.0966	790148.6178	28325.95241	315113.3166
372.25347	4.381983	4.35E-08	1.88E-07	671311.2059	254380.3072	21855.8824	580088.5168
510.35807	3.746783	4.25E-08	1.84E-07	1234094.861	2392932.367	10201395.1	2363237.216
250.11641	3.7865	4.21E-08	1.82E-07	195856.5622	437731.1875	12418.26888	171959.2427
389.15579	2.829167	4.14E-08	1.79E-07	714274.2824	355016.8503	90028.15885	518416.2857
349.22852	3.5615	4.12E-08	1.78E-07	6588732.268	8340068.069	904212.554	3095649.052
313.21621	3.52715	4.09E-08	1.77E-07	1853648.457	2736997.689	794079.9267	1518848.082
385.23492	4.034008	4.02E-08	1.75E-07	2993759.605	7263282.742	82679.77346	1773674.149
467.2617	2.977433	4.01E-08	1.74E-07	915959.5087	702012.6634	27529.471	378707.1379
437.06909	2.67205	3.91E-08	1.70E-07	1607848.635	1172037.694	166613.2547	531505.611
621.3116	3.2743	3.91E-08	1.70E-07	220655.1686	25436.58367	3240.047199	281431.4001
383.27922	3.330242	3.86E-08	1.68E-07	2408343.526	1820699.202	178656.4843	2052109.494
319.19054	3.483533	3.86E-08	1.68E-07	625390.5506	1011096.344	203306.0286	604162.3238
168.5653	2.543233	3.61E-08	1.58E-07	2856977.721	778830.3019	1383126.134	5723886.155
415.20916	3.144333	3.60E-08	1.57E-07	3364917.317	2966117.347	281250.1468	1890960.918
260.11294	2.662967	3.53E-08	1.55E-07	806648.0253	834510.8889	1803953.869	1176814.885
416.24324	4.382317	3.51E-08	1.54E-07	271452.624	227492.7377	4087.892253	169346.342
471.14435	3.631658	3.33E-08	1.46E-07	214745.1429	592980.4039	20471.83626	234854.9807
416.24324	3.378817	3.31E-08	1.45E-07	1949920.472	1427938.688	52594.74871	1706824.458
419.29044	3.724975	3.15E-08	1.38E-07	3585254.102	5898378.129	200667.965	2904790.926

Kruskal-Wallis significant metabolites positive ionization mode

366.06905	3.064417	3.10E-08	1.37E-07	3975697.66	2796042.666	367567.1677	1737772.829
202.16716	4.382	3.08E-08	1.35E-07	307653.5459	82682.17234	2154.223087	277428.4589
437.25127	3.116533	3.04E-08	1.34E-07	2183627.991	1946604.852	161107.9196	1300269.297
205.02781	3.553283	3.03E-08	1.34E-07	6044588.438	4779426.211	2083765.876	2460596.28
437.1865	2.596433	2.96E-08	1.31E-07	713770.4875	398813.0713	153855.8139	591889.2169
196.09682	2.218433	2.86E-08	1.26E-07	2775133.518	1435951.52	952126.1854	2676400.115
331.19042	4.556475	2.78E-08	1.23E-07	2093804.349	3175023.843	201423.185	1545345.506
399.24104	4.382308	2.74E-08	1.22E-07	470477.6164	263842.7564	12399.732	248692.0462
275.00734	0.975591	2.66E-08	1.18E-07	288611.8024	324871.481	1284160.878	791163.9989
385.23493	3.795333	2.60E-08	1.16E-07	23133678.75	55749828.4	575379.5002	16204186.25
383.2193	3.723392	2.56E-08	1.14E-07	44503071.52	117819390.3	1254895.862	27770977.9
261.14858	3.7256	2.45E-08	1.09E-07	857915.4533	1348061.23	150787.096	517768.8173
325.23738	3.439617	2.40E-08	1.07E-07	1062463.368	919561.0391	403246.4492	1138472.573
362.20431	3.475	2.40E-08	1.07E-07	10230411.88	18043357.05	290221.2197	7922940.798
377.19596	2.95965	2.31E-08	1.03E-07	2114215.204	2150715.877	419676.3717	1452661.324
456.08302	2.672175	2.28E-08	1.02E-07	836838.184	702699.1168	84022.09159	522837.8815
311.06297	2.98635	2.18E-08	9.76E-08	2280196.993	708691.4074	770673.8481	196136.6984
384.28256	3.334125	2.18E-08	9.76E-08	433708.8515	367506.2545	31305.33632	434059.3369
240.10329	3.725933	2.17E-08	9.75E-08	700794.407	1098847.578	43703.30745	435233.2382
384.22264	3.721133	2.17E-08	9.74E-08	10252647.5	27475667.57	310568.5152	6020248.124
203.07039	2.560333	2.10E-08	9.45E-08	1397649.933	2576244.228	536665.2471	964459.3328
221.15365	3.465583	2.09E-08	9.39E-08	617003.2075	577079.4599	4719404.393	510239.5898
311.20057	3.159217	2.08E-08	9.39E-08	5721842.12	10175215.48	1650813.607	4617948.975
312.20393	3.56205	2.01E-08	9.07E-08	590321.605	1292307.734	177460.7015	630854.5609
343.22685	4.049892	1.96E-08	8.84E-08	8171218.136	20836761.38	430760.6027	3990906.681
337.08876	3.082067	1.95E-08	8.81E-08	1747225.011	281816.1477	767413.1889	1439984.388
536.22094	4.382017	1.94E-08	8.78E-08	2117330.307	1113653.603	216422.2006	1979088.945
347.24929	3.821383	1.89E-08	8.54E-08	2867967.817	3509266.272	392551.2589	1467334.6
345.24249	4.04145	1.76E-08	7.97E-08	27436613.55	67752813.06	399853.9535	11657588.38
343.22688	3.7155	1.75E-08	7.93E-08	80181602.66	188652532.2	2719405.785	46757593.16
263.08154	3.5505	1.69E-08	7.67E-08	556611.4795	271835.8714	232404.737	145341.1432
495.19426	4.382033	1.67E-08	7.57E-08	911359.6536	278935.3036	4037.099994	1186719.537
176.55146	2.55925	1.63E-08	7.41E-08	462196.149	127603.4178	138338.5763	757267.6337
411.21423	4.031717	1.62E-08	7.37E-08	1357339.497	4951644.044	32307.35898	1049851.968

Kruskal-Wallis significant metabolites positive ionization mode

419.20403	2.987133	1.61E-08	7.32E-08	4355615.371	2595258.211	233715.8352	6818492.188
365.23239	2.7855	1.58E-08	7.23E-08	44873802.83	89127480.37	3080401.181	22705691.58
144.53649	2.2443	1.54E-08	7.03E-08	1240673.809	1214439.37	288113.2644	251877.8133
384.18629	3.474617	1.53E-08	7.01E-08	7390204.223	10444833.82	445684.9362	5337786.949
189.16378	3.9109	1.49E-08	6.84E-08	844152.168	1528547.379	85534.38896	555125.1703
431.15184	4.355817	1.42E-08	6.51E-08	1291159.604	3033868.746	13918.09409	1245451.103
346.24582	4.041817	1.41E-08	6.47E-08	5448123.784	15401905.37	49021.00435	2270574.028
153.0522	1.439762	1.40E-08	6.45E-08	2365469.831	1810904.404	1160625.073	1506715.704
219.17439	4.147117	1.35E-08	6.24E-08	1706094.667	1581555.616	3873989.586	1578085.343
360.05362	3.257092	1.36E-08	6.24E-08	650672.9121	170070.9132	190884.4077	151566.7695
415.20921	3.430117	1.27E-08	5.86E-08	2575306.055	4888057.979	418786.1132	1693286.678
250.12765	3.239517	1.27E-08	5.85E-08	423000.1412	1020323.307	2172605.978	253801.2028
420.24381	3.125033	1.21E-08	5.60E-08	4198988.551	8255306.376	251244.2252	1853324.616
190.56013	2.660483	1.20E-08	5.56E-08	1106956.02	1321502.119	2569384.371	1501794.707
200.09169	2.42925	1.20E-08	5.56E-08	1429775.98	664277.6423	644242.6453	1049320.614
437.21613	3.116017	1.18E-08	5.49E-08	3699778.363	3629005.514	93029.0812	1106570.407
400.19662	3.726333	1.14E-08	5.29E-08	867252.631	1583364.211	37721.33062	535478.1057
342.21458	3.344167	1.12E-08	5.22E-08	1342424.983	1297117.86	176452.1314	1469882.618
288.04349	2.671667	1.08E-08	5.01E-08	482074.7941	558858.726	27882.9856	225995.9572
307.08122	3.215717	1.06E-08	4.95E-08	813718.0548	210782.0284	370734.8782	61289.83149
311.20058	3.566375	1.06E-08	4.93E-08	4021666.898	7674873.638	1337308.175	3075526.569
363.08275	3.117217	1.05E-08	4.92E-08	1157044.723	2344377.883	216984.329	338404.4296
346.20943	2.977608	1.02E-08	4.77E-08	1782619.706	3437481.251	289779.0219	1442121.04
361.20098	3.475567	1.01E-08	4.72E-08	48435788.04	80650052.91	2046823.626	35632789.56
222.60659	2.784633	1.00E-08	4.69E-08	671420.5184	1534782.131	65295.07045	303085.6249
322.06949	2.838167	9.97E-09	4.67E-08	2369996.84	1324949.227	462411.1832	1319513.263
238.10504	2.548333	9.88E-09	4.64E-08	2028439.645	586757.3872	746600.6307	4172387.734
348.22508	2.792442	9.40E-09	4.42E-08	6606588.579	12891390.05	554087.6041	3700721.34
290.08121	2.61725	9.37E-09	4.41E-08	686067.2948	111754.7896	252363.7024	625998.5528
563.28282	2.827833	9.32E-09	4.39E-08	1282043.511	1590342.354	11151.39775	502845.4629
417.22483	3.43175	9.26E-09	4.37E-08	3940078.881	7779282.819	306542.9516	2572100.099
328.64359	3.195033	9.12E-09	4.31E-08	193578.6268	516501.7522	834728.3338	211664.8311
426.17266	4.353942	8.89E-09	4.20E-08	639103.1705	1479643.994	31992.41399	552061.3573
476.27736	3.689967	8.62E-09	4.08E-08	812819.4536	828142.9203	2718715.926	582524.4261

Kruskal-Wallis significant metabolites positive ionization mode

344.23022	3.715	8.49E-09	4.02E-08	19135874.48	43094068.64	604350.3901	11812084.75
221.05987	2.704892	8.47E-09	4.02E-08	504153.6619	514365.6213	460990.7382	308530.9454
207.13797	3.762042	8.36E-09	3.97E-08	593813.8367	567690.3901	1223990.416	493812.5907
279.12036	2.507392	7.99E-09	3.80E-08	1373770.949	1494966.359	463135.0668	649493.743
366.19544	3.167617	7.74E-09	3.68E-08	2408631.316	2965694.606	104847.801	1568940.844
327.21661	2.986058	7.64E-09	3.64E-08	1513317.984	1290458.32	404865.6634	1310502.132
328.23535	4.490475	7.58E-09	3.62E-08	361586.7862	978801.3076	23678.79994	363033.6636
339.08646	2.6637	7.51E-09	3.59E-08	887447.4038	1100237.182	2015513.341	1207824.17
341.21126	3.347067	7.33E-09	3.50E-08	7039785.556	6860806.573	1005018.529	6797442.328
400.21761	4.033433	7.22E-09	3.46E-08	1001518.513	1434812.88	16669.89845	767865.3987
360.22504	3.345492	7.20E-09	3.45E-08	2951651.153	3650053.818	324117.2404	2181598.022
422.30941	3.789742	7.19E-09	3.45E-08	391252.681	676445.6872	15502.44985	354307.4123
366.2357	2.7926	7.16E-09	3.44E-08	10937481.73	19954941.27	922530.6587	5570357.11
333.18905	4.381708	6.99E-09	3.36E-08	2037857.584	2926608.935	136397.818	2719056.317
381.22728	3.160617	6.99E-09	3.36E-08	3607216.237	6434168.627	469659.1209	2254611.636
421.19868	2.769567	6.89E-09	3.32E-08	1134924.169	1581135.765	266238.6724	1328675.205
430.22259	3.292083	6.74E-09	3.25E-08	176109.3529	163449.2948	6473.474867	292604.9838
226.14378	2.829517	6.56E-09	3.17E-08	1052935.201	307509.0627	2233893.561	1068264.422
376.25645	3.763025	6.50E-09	3.14E-08	159828.0576	620599.1375	5667.642519	217291.785
109.10157	3.562442	5.99E-09	2.90E-08	1181375.159	2211579.044	427658.8205	876289.1301
379.06038	3.116267	5.60E-09	2.71E-08	705584.2656	1448649.392	116731.8378	103613.7
273.10971	3.017983	5.03E-09	2.44E-08	72534832.67	34304785.96	17495140.67	3280379.573
359.22177	3.127125	5.03E-09	2.44E-08	7385159.857	11736427.17	719969.523	5202008.011
383.24112	4.382042	4.86E-09	2.36E-08	2540678.207	1628066.142	55251.47066	1774088.114
365.20893	4.34375	4.81E-09	2.34E-08	589192.4492	1473448.011	32583.70186	285632.2811
191.04153	2.717858	4.78E-09	2.33E-08	933520.3721	952422.242	579265.5263	582648.8698
365.06572	3.056033	4.74E-09	2.32E-08	21906899.04	14318439.82	1638591.976	10282759.21
411.23783	3.073392	4.69E-09	2.30E-08	521655.9412	1171829.084	102504.9834	791360.7084
556.22221	3.117917	4.68E-09	2.29E-08	225106.0386	6492.555905	3465.736931	274773.7404
421.3061	3.793833	4.66E-09	2.28E-08	2035806.622	3519692.917	164463.9034	1837981.619
273.13322	3.679992	4.54E-09	2.23E-08	4197950.186	3186291.748	389366.4982	3044022.992
317.08089	2.872117	4.37E-09	2.15E-08	2805563.856	2230437.208	425484.4032	1541701.952
363.20782	3.117333	4.16E-09	2.04E-08	1946750.529	2386469.818	247039.6976	1503666.33
487.05883	3.38805	3.85E-09	1.89E-08	495893.5071	386997.3007	6514.73776	393499.771

Supplementary A

Kruskal-Wallis significant metabolites positive ionization mode

455.14659	3.0733	3.66E-09	1.81E-08	1026497.99	520938.6776	85888.62522	942551.6256
153.05462	3.631667	3.50E-09	1.73E-08	1161633.52	1712674.009	585018.3064	1027352.911
431.20417	3.0473	3.50E-09	1.73E-08	1580857.281	2707034.343	168918.9268	855089.589
404.19172	3.112675	3.49E-09	1.73E-08	1381524.956	1423065.666	100877.0703	911565.306
406.22816	2.776883	3.33E-09	1.65E-08	6711415.771	9412690.708	414268.4135	2748213.01
225.10932	3.176017	3.30E-09	1.64E-08	1406639.119	2753825.037	543420.6425	1323495.429
364.25629	3.788883	3.29E-09	1.64E-08	7997662.8	20014185.18	67572.69731	5410714.217
175.57311	2.6933	3.21E-09	1.60E-08	1284565.488	413958.405	443339.1339	1663328.719
419.15171	3.155133	3.15E-09	1.57E-08	7661794.835	5169098.668	361578.0588	4550349.02
363.25291	3.79755	3.06E-09	1.52E-08	35000681.24	97762549.23	610251.7262	24031336.07
420.29376	3.7273	2.95E-09	1.47E-08	963783.1449	1694686.261	131303.7275	919197.7028
588.24975	3.330242	2.79E-09	1.39E-08	1718013.124	195351.3515	14730.62888	604930.586
144.98225	2.46305	2.72E-09	1.36E-08	136007869.2	50238421.21	46179280.04	117271260.3
416.27255	3.1172	2.62E-09	1.31E-08	397678.5997	634769.6435	11526.17958	144825.2984
405.22483	2.777883	2.44E-09	1.22E-08	31172877.02	46355062.59	1635491.783	13516700.38
226.12664	2.837108	2.42E-09	1.21E-08	732242.991	210672.1953	2027264.809	921652.4988
747.35533	3.562042	2.39E-09	1.20E-08	182024.83	1169983.219	2201.795225	173703.801
232.11804	2.47205	2.36E-09	1.19E-08	504713.5689	513554.4648	1242075.737	868522.8418
369.23142	4.487017	2.35E-09	1.19E-08	476707.9756	1365031.037	10293.33545	323595.6561
219.1744	3.3642	2.28E-09	1.15E-08	990391.7125	819896.4284	4448632.56	734643.2652
313.21621	4.389525	2.22E-09	1.12E-08	3509133.341	6825755.33	611846.3394	2984573.36
515.2618	3.576708	2.11E-09	1.07E-08	329704.3795	241156.7683	10814.57973	115437.7445
189.16378	4.49025	1.98E-09	1.00E-08	627024.5415	1712480.8	51311.16678	407017.6039
409.22212	2.92495	1.95E-09	9.86E-09	603564.9524	1045025.912	117178.1196	500113.8671
231.61191	2.780608	1.93E-09	9.79E-09	1565199.215	3710204.833	144620.4916	749105.0516
203.17948	4.1566	1.85E-09	9.38E-09	340908.0918	470706.5105	1972694.03	227463.5758
440.23521	3.135458	1.83E-09	9.33E-09	1656247.958	2063483.94	26659.31663	993258.5941
257.08082	2.8729	1.83E-09	9.30E-09	2143300.191	1054629.797	352475.7079	538387.449
543.25662	3.195467	1.80E-09	9.16E-09	429461.2024	281280.3965	11219.36322	183058.1189
381.20362	3.876033	1.75E-09	8.91E-09	617353.9762	1171401.861	57133.40453	575995.127
358.17061	3.26075	1.70E-09	8.71E-09	429890.0645	1007277.362	71925.63347	426496.0107
403.20916	2.57225	1.67E-09	8.57E-09	2693648.713	8160808.929	478252.6089	1866227.588
405.18684	2.506617	1.65E-09	8.43E-09	11191293.32	819527.2173	494104.3904	11654475.24
365.22327	3.51825	1.53E-09	7.83E-09	2165078.12	3740589.805	214715.4519	1640946.054

Kruskal-Wallis significant metabolites positive ionization mode

402.19697	3.099633	1.53E-09	7.83E-09	3228235.842	2963843.792	267931.2157	919106.4447
411.21191	3.221683	1.50E-09	7.70E-09	218369.5016	394455.1399	27563.53602	383076.3048
419.24045	3.1259	1.50E-09	7.70E-09	20307040.82	36758012.67	1299741.498	8373950.483
317.08087	3.954683	1.47E-09	7.56E-09	5865967.383	3930019.898	439907.8182	3063617.614
153.05221	1.683217	1.43E-09	7.36E-09	3949240.42	2710608.447	1490696.203	2658589.679
497.36674	3.90245	1.43E-09	7.36E-09	146016.1612	153985.8341	1726860.594	76316.73414
221.0886	3.125917	1.39E-09	7.20E-09	1678710.843	3682803.217	534711.1829	1075343.937
247.0489	2.106158	1.37E-09	7.11E-09	543127.543	505413.4547	209004.2323	368727.2639
391.24542	3.216783	1.34E-09	6.93E-09	949999.6048	1026800.89	113693.0856	731859.5143
362.06929	2.986533	1.32E-09	6.84E-09	833139.7108	179156.3817	153653.3062	54201.10242
103.95581	2.443358	1.30E-09	6.76E-09	2793913.781	4837605.927	4321206.377	3043344.236
267.12277	2.558767	1.29E-09	6.71E-09	1163713.773	1509256.275	3677137.523	700464.3426
229.58503	3.518333	1.28E-09	6.68E-09	177474.8593	678853.4431	9998.039643	259087.0434
496.36333	3.90225	1.26E-09	6.59E-09	692010.8425	667151.5371	6197673.988	385769.7418
379.21164	3.090975	1.25E-09	6.52E-09	7118735.771	12025694.9	742387.6578	4455447.231
390.23319	3.225933	1.24E-09	6.50E-09	4636326.679	6971865.184	268933.1657	2526550.325
422.18366	2.611433	1.23E-09	6.46E-09	933512.0812	961860.0369	46751.94086	502621.8191
388.19669	3.622733	1.22E-09	6.41E-09	676222.4747	776558.2645	111212.3255	652965.3611
318.08423	3.954283	1.17E-09	6.15E-09	1106712.307	705049.2347	44879.73323	595626.8718
363.20746	3.46705	1.13E-09	5.95E-09	1407896.794	1734255.718	137077.6377	902390.2054
367.24801	3.222067	1.09E-09	5.76E-09	10535584.79	17902293.01	888778.2858	6733975.273
422.22298	2.590508	1.09E-09	5.76E-09	946110.3714	1690170.516	68772.11967	636099.4327
435.23547	2.986008	1.07E-09	5.67E-09	3437131.653	4844808.563	403603.3985	1928510.119
346.12668	2.84615	1.01E-09	5.36E-09	451845.3418	57303.18879	48712.09592	296332.5034
233.03324	1.685	1.01E-09	5.33E-09	4430562.414	3488725.526	1361380.747	2867131.472
432.15526	4.3498	9.76E-10	5.17E-09	195648.8454	550770.0985	2511.321619	262761.4549
367.18807	3.31765	9.64E-10	5.11E-09	1940618.82	2571315.86	277777.7393	1551078.05
320.10856	3.082367	9.60E-10	5.10E-09	1184030.346	233528.606	184763.7967	1074791.547
455.20569	3.1346	9.35E-10	4.97E-09	675490.2045	635475.798	18264.16542	270681.7317
143.05339	3.0647	9.21E-10	4.91E-09	1084411.361	1357698.548	1622988.763	896071.7188
294.10516	2.87215	8.96E-10	4.78E-09	1493780.049	421765.5799	259644.7857	171044.0631
312.20393	3.1619	8.05E-10	4.30E-09	1104198.577	1886737.409	182554.0226	989552.6901
387.19334	3.591333	7.64E-10	4.09E-09	2325622.577	3253817.282	678468.1883	1765740.155
186.10115	2.836917	7.38E-10	3.95E-09	1274507.416	253377.5948	601090.6778	1037066.143

Kruskal-Wallis significant metabolites positive ionization mode

364.10676	2.557683	7.27E-10	3.90E-09	512904.0013	831828.0958	77682.5332	260760.1314
319.06037	3.1085	6.93E-10	3.72E-09	7099287.249	2853535.15	481357.6339	9526804.225
396.24632	3.465783	6.67E-10	3.58E-09	608188.5713	839736.0618	39212.00059	382342.4657
363.1778	2.908267	6.18E-10	3.32E-09	4092763.363	5863424.097	428699.4408	4301486.481
401.25175	4.381625	6.15E-10	3.31E-09	6257321.442	1673935.662	33811.53129	7677897.413
438.22498	3.282267	6.12E-10	3.30E-09	1800119.051	416188.4047	9706.707049	1185372.565
397.19864	3.927058	5.43E-10	2.94E-09	3892815.177	12814576.66	79468.74871	2321892.051
368.19139	3.710417	5.05E-10	2.73E-09	697745.3932	885717.6158	14650.3318	731441.3161
189.07854	2.556667	4.99E-10	2.70E-09	581468.699	282625.3535	231899.9414	1021523.292
231.06044	3.117083	4.88E-10	2.64E-09	1668633.534	2859297.732	455961.2022	328264.3596
287.16419	3.671708	4.80E-10	2.61E-09	807199.0686	1602604.539	131807.2469	751833.385
169.06691	3.117167	4.72E-10	2.57E-09	1815084.76	733856.9521	849470.1066	1477337.159
128.04247	2.243967	4.66E-10	2.54E-09	1345670.982	1318451.459	694092.6363	564765.2093
249.11984	3.338342	4.55E-10	2.48E-09	934242.6798	903827.955	14388363.6	696646.744
222.12053	3.321675	4.48E-10	2.45E-09	344988.9332	201961.6965	8995984.982	215368.2067
402.25535	4.39095	4.38E-10	2.39E-09	1311755.809	836751.0369	10034.50668	1150401.046
380.21505	3.099083	4.12E-10	2.25E-09	1073475.41	2212693.11	64288.834	567711.5255
465.15729	3.143483	4.07E-10	2.23E-09	802808.8246	3057894.491	115292.278	629944.9967
127.03918	2.244458	3.78E-10	2.08E-09	27528141.84	23053404.39	10574855.17	9081332.371
193.04966	2.582417	3.73E-10	2.05E-09	3602366.783	3781360.47	921040.1283	1321369.215
437.25107	2.863708	3.71E-10	2.04E-09	753062.7259	1116128.375	22953.58303	495504.0725
220.10809	2.387217	3.69E-10	2.03E-09	623917.2337	641284.4445	1965332.67	441482.3812
244.62944	3.2223	3.68E-10	2.03E-09	165885.275	621767.4886	4583.388757	114902.7694
187.08114	2.643042	3.67E-10	2.03E-09	1403305.937	157558.7413	412335.062	838061.0608
322.17302	2.81025	3.56E-10	1.97E-09	1129001.904	3333569.219	90340.8321	1610345.705
332.18574	4.382042	3.52E-10	1.95E-09	12037278	13138422.81	77610.92618	7500025.487
413.22985	4.155167	3.36E-10	1.86E-09	544898.0515	1605982.666	36177.82271	811978.0852
414.22759	3.443192	3.29E-10	1.83E-09	390391.3784	188340.0789	9194.844534	256773.1941
223.61443	3.227567	3.28E-10	1.82E-09	499881.9521	1691655.667	15309.70081	274231.3984
375.17795	3.098417	2.98E-10	1.66E-09	1587192.128	1708045.353	148055.3626	987872.5606
383.21921	4.491017	2.90E-10	1.61E-09	1012995.38	1988415.801	21736.39703	784145.8684
432.17772	3.56205	2.86E-10	1.60E-09	358998.7536	779133.5322	10548.15913	486681.8473
546.27573	3.108633	2.85E-10	1.59E-09	366970.2216	648765.382	5732.617619	110985.5895
269.14967	2.663775	2.82E-10	1.58E-09	1017319.957	284726.4263	8914281.622	916940.0784

Kruskal-Wallis significant metabolites positive ionization mode

368.25132	3.221767	2.80E-10	1.57E-09	1901012.136	3443530.188	50999.28222	1181804.765
382.2378	4.382008	2.80E-10	1.57E-09	2278108.887	819226.5379	3696.644944	3613997.49
326.16785	2.9241	2.54E-10	1.43E-09	421681.2027	536367.8055	58534.7221	453643.8699
385.34649	4.9316	2.40E-10	1.35E-09	1000312.773	1563749.837	372097.8623	1359474.786
534.27587	3.108483	2.37E-10	1.34E-09	1052739.149	1177383.01	6495.171809	852856.2575
213.13156	2.64995	2.20E-10	1.24E-09	16286939.42	1824028.767	3153209.939	10951299.23
364.25627	4.043017	2.08E-10	1.18E-09	1902236.396	5181596.156	23914.94239	943679.8285
545.27243	3.108383	1.96E-10	1.11E-09	2305053.758	2816025.784	52482.21192	1269497.76
321.16971	2.810158	1.84E-10	1.04E-09	6676632.355	19413986.26	483434.8982	9153560.829
190.06169	2.7207	1.81E-10	1.03E-09	704052.1835	471008.167	178971.6657	359066.8682
209.05218	2.722217	1.81E-10	1.03E-09	1087125.302	867502.8512	1460033.911	440232.3414
453.06391	2.773867	1.73E-10	9.82E-10	626823.0828	609310.6765	40851.69644	362269.2184
111.02062	2.129133	1.66E-10	9.44E-10	69453164.47	19214968.34	16467509.75	62838249.77
431.17435	3.56195	1.63E-10	9.31E-10	1560608.977	4094243.347	78634.45317	1302322.733
214.6091	3.23055	1.57E-10	8.97E-10	362946.0259	1033962.648	11940.15201	284678.9498
399.19339	3.72145	1.56E-10	8.93E-10	2792696.669	3609927.283	64307.77779	2274693.543
393.22716	3.422433	1.48E-10	8.48E-10	3214115.343	5910222.227	379036.2032	1825470.644
341.07971	3.073517	1.47E-10	8.45E-10	1519852.574	356937.7966	136218.0136	1380340.358
263.07021	2.837108	1.45E-10	8.34E-10	3269605.362	414063.096	710065.5266	2679265.423
485.18663	3.6282	1.45E-10	8.34E-10	214385.5695	528864.747	7365.110903	266689.0313
187.14815	3.56595	1.31E-10	7.57E-10	578135.8865	1330588.636	172663.9794	545700.9215
403.18837	3.115967	1.28E-10	7.40E-10	4860417.534	6342694.339	329940.843	2672648.206
474.21648	3.236367	1.25E-10	7.25E-10	339818.4238	935819.7657	15035.91677	294548.26
321.07276	2.8377	1.21E-10	6.97E-10	4176469.729	4222766.46	287310.82	2337383.452
511.2906	3.1164	1.17E-10	6.80E-10	602760.8124	1427540.701	9247.150658	583851.7276
109.10157	3.169658	1.15E-10	6.68E-10	1097053.92	1934913.133	319197.6189	740536.4986
125.02344	3.064758	1.14E-10	6.63E-10	1090676.683	417847.7243	444080.4407	790399.1913
363.25295	4.224267	1.14E-10	6.63E-10	7199105.021	14156817.17	257516.7612	4032126.696
111.02061	2.926017	1.09E-10	6.33E-10	28377456.36	14501960.23	13401942.65	28707627.41
599.24966	3.1764	1.06E-10	6.17E-10	481250.1756	13633.76886	10801.74785	184387.4969
331.22685	4.362067	1.05E-10	6.14E-10	124043747.2	257011620.2	5509322.898	100967863.2
332.23018	4.36445	1.04E-10	6.07E-10	27175439.9	56618832.68	472275.4991	21054266.98
247.09653	2.898967	1.02E-10	6.00E-10	1148327.479	678943.4545	3131904.784	455812.5001
387.24162	3.790283	1.03E-10	6.00E-10	1016434.298	2064375.472	67669.4305	800135.645

Kruskal-Wallis significant metabolites positive ionization mode

327.19548	2.898983	9.60E-11	5.64E-10	2410969.028	3882949.383	600429.4893	2135886.662
446.15567	2.7765	9.14E-11	5.38E-10	504173.874	35779.59604	80612.69486	24460.08118
262.10749	2.651008	8.56E-11	5.04E-10	2490557.178	1391984.003	578040.3783	399947.1413
182.57039	3.027183	8.48E-11	5.00E-10	20259.01751	39267.58038	1891564.303	45544.10058
385.2258	3.722967	7.98E-11	4.72E-10	1801040.188	3989076.016	113191.462	1537083.144
215.05215	2.986292	7.87E-11	4.66E-10	3939405.779	3355955.2	1779549.442	1007893.661
400.21765	3.352192	7.88E-11	4.66E-10	1965003.086	2180818.126	65758.76118	1341870.49
347.22173	3.924883	7.77E-11	4.61E-10	12027393.15	23897986.89	723531.5907	7675547.283
287.16422	3.161075	7.41E-11	4.40E-10	892789.9705	1698092.636	133936.6124	973548.0278
190.03366	3.109975	7.10E-11	4.22E-10	4673815.537	2183425.703	11896505.69	10447126.82
335.09141	2.541217	7.10E-11	4.22E-10	5735406.817	5908802.399	196940.7811	3490712.446
247.13297	3.161417	7.06E-11	4.21E-10	2681733.262	4419115.655	371425.7627	1460862.883
444.2123	4.381667	6.96E-11	4.16E-10	613159.6717	592415.502	4319.300674	1050923.29
395.24296	3.4667	6.90E-11	4.13E-10	2673413.975	4521775.831	255331.0794	1792482.925
391.21148	3.047367	6.45E-11	3.86E-10	1156142.094	2430641.14	164675.7818	918975.2126
343.29948	4.149475	5.70E-11	3.42E-10	836683.2779	1323074.025	73039.35994	421510.2225
428.33723	3.33425	5.67E-11	3.41E-10	234129.264	242623.1476	8186.919928	397193.0881
207.05469	2.879467	5.60E-11	3.37E-10	969536.0674	530239.1021	676686.3722	262575.2359
289.17984	3.4903	5.58E-11	3.36E-10	1254805.921	2087550.275	125396.4127	1121757.414
439.23178	3.143258	5.56E-11	3.36E-10	7894767.519	3260458.654	67618.12498	6390707.639
358.20934	3.499533	5.53E-11	3.34E-10	333148.2011	573878.2246	9709.14147	278495.4511
433.19025	3.62925	5.45E-11	3.30E-10	1412996.025	1895956.883	233371.7422	1340913.88
242.11204	3.179483	5.19E-11	3.15E-10	1550354.255	2750701.888	102185.8854	1397193.968
384.20599	3.139033	5.19E-11	3.15E-10	3086582.042	4412832.468	84542.79645	1720862.838
261.10845	3.223467	5.03E-11	3.06E-10	1897129.97	952877.7587	417883.7232	557153.1816
374.18145	3.561967	5.02E-11	3.06E-10	312419.1959	1757584.33	6754.114449	349842.3383
121.96642	2.432383	4.98E-11	3.04E-10	694950.2004	226450.4085	194380.6832	595558.0122
355.18801	3.544833	4.99E-11	3.04E-10	387233.1964	764792.761	26852.66116	486850.1033
235.13286	3.688483	4.90E-11	3.00E-10	2258133.922	3376859.404	764456968.7	1761421.363
361.23725	4.1544	4.86E-11	2.97E-10	9710915.811	25068710.09	911402.2261	6656613.342
407.06187	3.038308	4.77E-11	2.92E-10	18782.2624	119229.2021	1085503.876	4546.114614
362.20438	3.117258	4.35E-11	2.67E-10	9539000.914	15595736.43	155203.3377	7541356.86
386.27714	3.544367	4.33E-11	2.66E-10	859881.9989	730356.5286	24709.43618	254129.1808
460.26941	3.631667	3.91E-11	2.41E-10	351518.8974	210910.5916	1781576.757	769832.2624

Kruskal-Wallis significant metabolites positive ionization mode

331.21806	3.561983	3.62E-11	2.24E-10	7297137.17	12987191.09	1000206.074	5542383.287
365.23246	3.117117	3.49E-11	2.16E-10	99353585.79	152387385.3	2296462.456	61471355.24
533.27228	2.854483	3.45E-11	2.13E-10	610061.3102	602793.216	2840.330064	360105.8568
421.21963	2.719633	3.42E-11	2.12E-10	2482609.628	5662214.759	267662.6924	1542657.524
399.21427	4.034067	3.39E-11	2.11E-10	2238627.666	4279011.667	35122.48801	1623525.935
345.1809	3.07325	3.08E-11	1.92E-10	3707144.617	4516768.461	232975.2322	2315719.405
400.21778	3.133592	2.95E-11	1.84E-10	1422608.789	3176492.856	54055.19291	749336.8894
217.12248	3.742425	2.87E-11	1.79E-10	323954.6479	437582.5525	1253669.71	398005.6884
230.59286	3.117375	2.57E-11	1.60E-10	521812.0173	2115351.701	38831.05975	270448.8469
366.23581	3.11695	2.36E-11	1.48E-10	20545499.42	33900328.13	472181.3599	13786338.11
318.08407	2.8496	2.36E-11	1.48E-10	1367491.334	626757.3609	166621.1554	1184716.186
451.20047	3.230108	2.30E-11	1.44E-10	926775.2387	2281079.191	57871.89559	799194.385
459.16764	3.519	2.26E-11	1.42E-10	1018749.564	700017.6915	90276.21242	738153.9711
184.09687	2.569342	1.92E-11	1.21E-10	3662600.01	1528290.195	1232046.094	4315093.829
327.18023	2.942283	1.87E-11	1.18E-10	956799.9714	1230138.633	227493.2537	990525.823
348.25284	4.494975	1.81E-11	1.14E-10	476664.8131	1026376.021	8410.752792	304419.6272
378.23563	4.034225	1.59E-11	1.01E-10	590861.085	1711916.039	13210.84296	497282.1988
242.07638	3.539567	1.47E-11	9.32E-11	2734984.451	3459587.565	54835.07363	2449303.329
344.08053	3.037008	1.41E-11	8.92E-11	373781.9378	78721.77559	814813.6935	240339.7371
154.04964	3.064767	1.32E-11	8.39E-11	1714437.732	518046.8752	649269.4342	1660299.288
531.25668	3.0647	1.31E-11	8.32E-11	10475491.99	10019278.78	359566.7382	5192360.765
391.24787	4.153633	1.30E-11	8.31E-11	1356247.377	3429011.008	40503.2178	1801996.358
347.1466	2.933883	1.29E-11	8.25E-11	2471973.019	2639717.822	129589.9128	1043894.013
415.26919	3.117542	1.28E-11	8.20E-11	3314206.175	3391502.305	66490.61355	1217584.624
245.08091	2.986117	1.28E-11	8.19E-11	979855.8437	880867.1113	309189.2832	328804.9544
282.13365	3.652483	1.27E-11	8.13E-11	635260.87	10275.75522	40053.55078	452934.5471
203.1795	3.187167	1.22E-11	7.84E-11	180778.4533	206852.3918	29984184.56	150091.29
113.06006	1.70225	1.20E-11	7.70E-11	20582337.41	18270019.57	3922505.872	13198668.53
532.26005	3.06475	1.19E-11	7.68E-11	2436268.155	2787793.765	50931.16824	1515852.821
604.27174	3.422275	1.16E-11	7.50E-11	719347.8954	32814.56447	96822.96848	488395.2878
413.1937	3.090867	1.14E-11	7.37E-11	664627.2001	1415243.713	33443.69928	426592.229
337.10717	2.651442	1.10E-11	7.11E-11	2426584.209	399035.274	354784.0176	1164785.722
365.2598	3.781658	1.02E-11	6.60E-11	1357358.764	2618162.736	67546.77986	1052317.583
398.23271	3.671342	9.90E-12	6.42E-11	4091898.663	3986386.652	42054.86573	3534167.057

Kruskal-Wallis significant metabolites positive ionization mode

371.21935	3.23105	9.82E-12	6.38E-11	14897132.49	16830008.9	359624.8959	10787241.85
400.14329	3.11705	9.45E-12	6.15E-11	362149.2673	863762.2134	2829.623367	188377.5622
357.20605	3.485483	9.18E-12	5.98E-11	1617206.338	2299759.509	178469.3024	1784791.017
275.08902	2.898133	9.09E-12	5.93E-11	2381640.721	656887.7364	874170.9845	227519.2751
435.23788	3.68405	8.81E-12	5.76E-11	545229.5009	704824.2483	25016.58359	369067.0767
221.1173	3.16085	8.71E-12	5.70E-11	2602469.717	3730524.437	130879834.8	1555414.842
509.27478	3.064733	8.62E-12	5.65E-11	1194284.123	2857969.087	14424.49286	752429.8041
234.61629	3.562225	8.40E-12	5.52E-11	566753.4749	1795810.683	6907.109253	533533.8824
147.02881	0.743704	8.34E-12	5.48E-11	1232534.042	2018174.648	2143423.009	1557280.998
395.24297	3.212842	8.24E-12	5.43E-11	2426172.825	5165940.483	443388.7569	1716818.723
433.21984	2.986117	8.17E-12	5.39E-11	2359652.582	2784686.429	111772.0481	993908.9532
399.15702	3.475358	7.86E-12	5.19E-11	796130.4339	1090458.517	35498.31138	756036.4152
381.22721	2.871925	7.59E-12	5.02E-11	3591945.777	7876250.026	540272.2741	2596456.282
131.07041	1.685117	7.33E-12	4.86E-11	54036193.78	38742662.86	6627566.009	35235248.89
341.06326	2.5959	7.06E-12	4.69E-11	1271985.424	859094.7348	277643.922	316447.3405
155.07025	2.625083	6.96E-12	4.63E-11	1210612.059	1223764.466	6789182.992	1163058.048
157.16994	0.400124	6.88E-12	4.59E-11	101263.5399	314710.3098	402490.2196	120250.5382
131.0704	1.452023	6.74E-12	4.50E-11	29560172.61	21263538.63	3879682.031	19599672.83
400.24843	4.38235	6.67E-12	4.46E-11	25497552.35	8889324.157	80480.64687	31274516.63
215.0226	1.684983	6.35E-12	4.25E-11	2007371.07	1445182.838	319666.799	1281910.378
351.24416	3.23615	5.58E-12	3.74E-11	4585318.435	7391079.729	612177.4032	3784044.616
530.03171	3.474967	5.44E-12	3.65E-11	306600.5306	112073.9483	1344.476288	28298.74012
314.21967	4.479383	5.40E-12	3.63E-11	392445.9501	1317539.307	16048.00166	323397.9219
359.2217	3.355158	4.76E-12	3.20E-11	13166751.01	16089760.86	606628.17	9533281.276
224.08544	2.824575	4.65E-12	3.14E-11	1048178.595	290717.136	370585.1454	1085832.031
212.58217	3.562033	4.63E-12	3.13E-11	981949.5806	2148953.513	30625.22354	905970.9802
391.14441	3.3218	4.61E-12	3.12E-11	1277534.114	41431.14597	80027.30462	845334.6166
350.07976	2.7257	4.50E-12	3.05E-11	921156.6797	115805.1458	143524.712	1011523.064
364.21995	3.178017	4.36E-12	2.96E-11	11892883.87	22579862.17	813331.0758	8012915.703
221.19002	3.1961	4.17E-12	2.84E-11	777570.9857	1109517.694	7039944.695	822390.6183
235.1693	3.1736	4.10E-12	2.80E-11	814960.4803	839379.7851	7958683.574	587179.7644
189.16386	3.163167	3.97E-12	2.71E-11	1564275.003	2736323.56	101522.3082	886140.416
417.24972	3.1428	3.82E-12	2.61E-11	6786772.266	6460762.99	100283.0246	4644862.154
222.59534	3.631142	3.71E-12	2.54E-11	1077298.057	2029232.53	19656.7082	945371.6492

Kruskal-Wallis significant metabolites positive ionization mode

404.28775	3.39825	3.57E-12	2.45E-11	412752.0079	638579.2265	14541.89568	153894.67
235.13294	4.486883	3.53E-12	2.43E-11	642995.4312	1209926.978	5684647.611	471048.2651
590.15452	3.358467	3.38E-12	2.32E-11	509924.4322	100952.1848	2728.344135	3394.545477
127.07552	2.611183	3.21E-12	2.21E-11	1978517.105	1939962.639	560019.5233	722839.2005
472.17849	4.408233	3.10E-12	2.14E-11	175563.9474	834968.5612	1776.481601	150058.4446
241.10423	3.474625	3.05E-12	2.11E-11	1311320.871	2827400.101	78831.17565	1154718.835
375.21667	3.928758	3.04E-12	2.10E-11	2772293.038	7971881.736	64775.1457	1867470.206
247.13297	3.561775	2.95E-12	2.05E-11	1953416.867	4012103.647	230674.274	1407967.193
399.27421	3.8938	2.94E-12	2.04E-11	831061.8042	1373919.118	202994.62	638416.6459
386.18096	3.562017	2.87E-12	1.99E-11	1178344.962	1425959.855	19329.25103	898533.5149
389.22976	3.12665	2.84E-12	1.98E-11	6094972.467	9706035.564	372304.8996	3381110.39
233.11729	3.651633	2.77E-12	1.94E-11	1119696.227	1841358.459	16615424.68	873835.273
377.23229	4.034233	2.68E-12	1.87E-11	3273017.895	8436456.896	105213.8735	2767708.383
400.21767	3.649283	2.66E-12	1.87E-11	1608824.157	3974530.21	22186.40876	957590.9277
433.18988	3.231133	2.61E-12	1.83E-11	425765.5613	863754.1075	30940.528	443495.6913
385.17779	3.174817	2.54E-12	1.79E-11	3228418.076	2243868.799	125236.4242	4169777.498
385.27372	4.102983	2.42E-12	1.71E-11	929006.4178	1773451.578	58689.30065	431081.1
310.06093	3.0736	2.40E-12	1.69E-11	4271081.349	5524405.164	129366.8605	2418626.578
309.07579	2.837058	2.36E-12	1.67E-11	1088458.102	137162.0783	275203.8311	909966.4687
243.61038	3.631392	2.31E-12	1.63E-11	663509.5094	1149135.503	7038.588716	745381.3733
385.17773	3.562067	2.30E-12	1.63E-11	2694561.811	4649118.377	175643.2412	2343746.916
251.09145	2.68685	2.28E-12	1.62E-11	1206548.773	737429.077	1414767.619	210233.7297
375.21669	3.239108	2.27E-12	1.62E-11	4078508.598	8291112.625	328654.119	3672297.783
362.10489	2.609567	2.11E-12	1.50E-11	427326.4483	39526.99229	53108.67838	259220.7186
347.22178	3.169317	2.07E-12	1.48E-11	264854542.8	410722647.3	3381131.182	139321368.2
399.21432	3.352783	2.05E-12	1.47E-11	9713182.469	10129184.69	265371.9495	7358997.195
418.2281	2.976883	2.00E-12	1.43E-11	1563340.363	2085354.936	138477.9838	974587.7696
415.23412	3.1172	1.99E-12	1.43E-11	5132392.857	5155419.778	164472.172	2938601.638
403.2092	2.87335	1.97E-12	1.42E-11	9608826.385	20562528.57	409255.3437	6479845.228
348.22512	3.169358	1.93E-12	1.39E-11	57970253.22	93466561.59	786792.5599	30454552.74
431.05467	2.98605	1.85E-12	1.33E-11	799604.9892	271347.6084	360648.5596	26784.36684
321.16989	3.125658	1.84E-12	1.33E-11	13851441	26226241.93	579682.7107	18260886.73
332.06344	3.423983	1.83E-12	1.33E-11	886306.0798	261222.5264	16435.57223	814966.2965
283.16538	2.651617	1.83E-12	1.32E-11	1100191.712	338392.3848	93034.49452	1161698.847

Supplementary A

Kruskal-Wallis significant metabolites positive ionization mode

366.69712	3.560275	1.81E-12	1.31E-11	163075.4223	1281207.753	1364.263088	72302.19453
139.07534	2.837083	1.73E-12	1.26E-11	2850088.678	891914.3855	988438.1148	2376055.899
439.18219	3.073333	1.73E-12	1.26E-11	2776346.151	2822186.572	60717.50304	1895525.302
134.03681	2.246258	1.68E-12	1.23E-11	1924026.248	732585.3509	75489.2307	631071.2503
244.12776	3.225933	1.66E-12	1.21E-11	1028095.073	2617732.019	22126.96916	567746.6707
348.10058	3.309	1.63E-12	1.19E-11	462466.6731	15868.81399	33021.70818	330131.3275
417.2248	2.977358	1.61E-12	1.18E-11	7042216.636	10159902.73	417034.2082	4306778.293
367.18807	3.710283	1.59E-12	1.17E-11	3561398.828	4507295.464	134855.7496	3096354.732
357.16728	3.261733	1.55E-12	1.14E-11	1914736.599	4885642.168	111529.3917	1948306.871
396.03612	3.117092	1.54E-12	1.14E-11	719348.8954	846303.6098	7232.334306	27234.95225
371.20651	2.855392	1.47E-12	1.09E-11	670436.0207	1004129.417	71138.81116	658880.0103
421.18185	2.61625	1.42E-12	1.05E-11	10362532.13	8081092.129	367026.8096	5745461.13
236.09987	2.653292	1.34E-12	9.95E-12	3032832.856	1829013.065	367496.927	473291.4464
533.2725	3.110608	1.34E-12	9.93E-12	4768399.589	4159042.314	35182.50174	3239292.253
417.06873	2.985933	1.32E-12	9.85E-12	831044.365	245389.0434	305609.1531	21267.3783
393.22718	3.218783	1.27E-12	9.48E-12	2493885.098	4370040.28	231579.77	1832779.22
275.07969	2.986008	1.25E-12	9.37E-12	1243250.66	492730.0462	1015725.68	50707.98596
388.21186	3.673825	1.20E-12	9.00E-12	1477966.225	5586101.908	163410.3577	1039501.172
401.26878	3.631883	1.20E-12	8.98E-12	254250.6561	630836.6454	5315.084393	96147.27151
228.12313	2.625583	1.17E-12	8.80E-12	1224650.846	200329.2632	450189.3211	1363962.985
261.14866	3.151783	1.16E-12	8.74E-12	2280104.357	1228052.932	439580.4854	820768.4024
243.10867	3.631733	1.15E-12	8.64E-12	2700895.626	5171944.794	135033.3023	2860100.42
191.07029	2.828783	1.13E-12	8.54E-12	20954769.03	8438716.294	1126318.913	3967051.482
377.23235	3.143058	1.13E-12	8.54E-12	6324672.212	11435672.29	467630.3265	4529746.918
156.10197	2.575783	1.13E-12	8.54E-12	5699455.827	1301682.074	1678315.713	5943902.59
305.10017	2.837767	1.11E-12	8.43E-12	4201113.206	194291.5396	486579.213	3494363.045
322.1731	3.126	1.10E-12	8.36E-12	2151092.24	4409787.779	34365.71745	3534271.117
346.20945	3.711	1.09E-12	8.28E-12	1531974.457	2127484.157	31624.5514	1071568.91
399.21438	3.133667	1.04E-12	7.95E-12	8124590.788	13001510.26	213090.6227	5204630.476
242.11202	3.518617	9.91E-13	7.58E-12	1505994.541	2738673.597	26912.07817	1195771.029
401.14107	3.562567	9.67E-13	7.41E-12	434598.0396	1207138.295	8204.381134	329742.1054
199.03904	3.116517	9.54E-13	7.33E-12	465259.7351	732345.8157	15199.14503	31353.02092
399.21433	3.64905	9.51E-13	7.32E-12	7336212.577	17811284.02	111093.8166	5458111.56
440.17462	3.467925	9.39E-13	7.24E-12	369920.5805	880451.1357	6710.608864	350753.4345

Kruskal-Wallis significant metabolites positive ionization mode

360.22502	3.873758	9.23E-13	7.13E-12	884443.7735	1657785.936	61055.93704	644808.4333
588.15493	3.354833	9.21E-13	7.12E-12	2046278.5	163084.9384	2799.173296	517550.038
243.62164	3.134508	8.27E-13	6.41E-12	650986.1685	3375089.556	32101.59166	316678.3129
376.22003	3.583158	8.25E-13	6.40E-12	3813358.138	8353728.954	68341.85456	3300926.447
359.22173	3.872433	7.94E-13	6.17E-12	4893757.874	10204622.84	502654.8084	3055072.849
420.1792	3.07335	7.87E-13	6.13E-12	11686452.44	8414189.844	139658.871	4896131.293
345.20617	3.518367	7.74E-13	6.05E-12	4689439.319	8672519.914	493169.4176	3495590.834
407.16656	2.52435	7.60E-13	5.95E-12	498481.4645	116282.1291	74771.03913	362043.2536
213.60125	3.562117	7.55E-13	5.92E-12	447054.2704	1402101.311	13413.63684	293254.4695
421.2198	2.999175	7.33E-13	5.76E-12	1528622.042	1862340.402	84435.19655	1069636.492
210.11251	3.021817	7.25E-13	5.70E-12	1010585.342	208220.4862	222059.9787	1622177.505
329.21121	3.913383	7.16E-13	5.65E-12	13688788.2	33872176.94	429666.9828	7454569.227
319.12651	2.610417	7.14E-13	5.64E-12	1515738.595	928755.4107	353445.5463	148620.9589
289.07071	2.995242	6.95E-13	5.50E-12	3366215.119	1951412.166	2311921.448	381779.9922
313.06422	3.5445	6.94E-13	5.50E-12	1696578.073	939580.3423	94577.89698	142301.5319
365.23242	3.553117	6.93E-13	5.50E-12	13913957.74	36425285.98	807364.573	9125690.596
189.16379	3.560817	6.74E-13	5.36E-12	1210053.787	2738368.079	133414.3943	942172.8524
385.2738	3.152383	6.39E-13	5.09E-12	1443890.455	1223549.059	4534.025826	891238.2745
361.20105	3.117092	6.26E-13	5.00E-12	44577385.06	70477956.15	769372.8273	27255844.53
297.16974	2.92475	6.00E-13	4.80E-12	2194216.38	2703569.252	270460.546	1443972.419
602.26515	2.837208	5.81E-13	4.66E-12	264626.2918	2755.852058	3622.654534	178166.1544
330.21454	3.919617	5.79E-13	4.65E-12	2558568.445	6741646.295	46463.65253	1534746.326
449.18484	3.14295	5.74E-13	4.62E-12	2079744.43	9999217.767	80316.00553	1377474.376
218.13872	2.465342	5.51E-13	4.44E-12	2362613.471	389202.7738	484552.0225	1908343.608
456.14691	3.472383	5.37E-13	4.34E-12	830957.8343	1566384.647	2276.92042	546204.9177
375.21671	3.579433	5.27E-13	4.27E-12	18741566.12	38356933.39	228127.3054	12660276.81
235.62411	3.62895	5.11E-13	4.15E-12	435872.2357	1141402.671	7427.771038	399092.4423
468.14207	2.829392	5.01E-13	4.07E-12	255534.1485	3995.31713	12167.57145	259745.519
584.28967	3.238508	4.38E-13	3.57E-12	358374.2768	7776.337697	5579.400773	232426.4539
415.18403	3.064733	4.31E-13	3.52E-12	2459131.506	904884.7739	36768.08344	857655.608
404.21253	2.87225	4.20E-13	3.43E-12	2022637.657	5100554.425	167355.2026	1418617.329
113.06006	1.438148	4.13E-13	3.38E-12	10611748.92	7914430.109	2447918.358	7326773.522
371.21044	3.561725	4.06E-13	3.33E-12	3787504.699	6897074.006	293887.3416	2869735.341
442.16768	3.562	4.04E-13	3.32E-12	1167966.573	2198034.382	9644.733457	865510.2582

Kruskal-Wallis significant metabolites positive ionization mode

384.18651	3.117083	3.90E-13	3.22E-12	2816371.843	5071907.532	38150.15221	1412338.234
383.18293	3.475467	3.86E-13	3.19E-12	34909949.43	47921489.07	339662.0708	22952262.85
114.51679	2.441117	3.76E-13	3.11E-12	1655622.797	163027.2604	132123.5661	874919.5158
403.28438	3.398667	3.62E-13	3.00E-12	1523313.81	2451848.596	23548.77787	611314.0685
345.20613	3.16075	3.60E-13	2.99E-12	8186388.641	11694176.33	368692.0476	6994602.412
241.60584	3.467325	3.39E-13	2.83E-12	275941.7165	761308.5532	4339.788675	223438.5905
399.1779	2.968433	3.32E-13	2.77E-12	968316.886	1211870.396	37197.96605	591932.9711
378.2357	3.15225	3.26E-13	2.72E-12	1237114.643	2615458.425	42677.79985	776019.1413
715.41819	3.176425	3.09E-13	2.59E-12	382506.5043	604324.6866	1480.091373	136947.1637
334.10733	2.725975	2.90E-13	2.43E-12	920404.4112	117320.0867	248195.6783	1057852.418
269.08752	2.837033	2.87E-13	2.41E-12	1185369.682	205674.1245	5189564.686	1106590.634
514.05942	3.474917	2.84E-13	2.39E-12	257711.1086	180222.9946	1366.177509	17039.07911
614.26389	3.099667	2.81E-13	2.37E-12	753903.8856	13716.2774	3070.532389	269938.9189
378.23566	3.640092	2.77E-13	2.35E-12	3494305.87	7947046.09	29526.50719	2246197.514
364.21998	3.524217	2.66E-13	2.25E-12	14068050.31	25915776.78	139766.9461	10529887.03
376.1971	3.623483	2.65E-13	2.25E-12	568732.5649	1773387.17	6534.008917	661265.1994
402.15482	3.0038	2.50E-13	2.13E-12	1906744.098	860451.807	262637.195	331753.7766
258.1128	3.108217	2.45E-13	2.09E-12	1482353.531	217114.3791	60159.93739	1367712.476
265.10711	3.038492	2.38E-13	2.03E-12	641873.2784	745330.1781	617536640.3	561284.949
597.23848	3.559192	2.38E-13	2.03E-12	275920.9412	2566.631201	1590.457632	206606.3617
290.07402	3.2827	2.36E-13	2.03E-12	922950.2017	531446.851	118390.7423	112398.9818
329.21115	4.2076	2.34E-13	2.01E-12	6968502.3	15183383.03	506553.7377	4008936.137
221.1173	3.56565	2.30E-13	1.98E-12	1999059.434	3705230.646	25981477.05	1412003.062
125.09628	3.064917	2.18E-13	1.89E-12	1843196.626	752601.2182	869253.7767	1423181.801
387.21423	3.5444	2.15E-13	1.86E-12	11096466.64	36256186.54	1176252.473	4686390.221
220.5602	2.721475	2.11E-13	1.83E-12	2118109.092	1669051.815	987558.4926	890233.5068
225.12336	2.837117	2.08E-13	1.81E-12	3388535.939	621545.5359	20211442.66	2876714.133
308.08971	2.836717	1.97E-13	1.72E-12	636604.6585	34405.42268	77774.25792	657729.4632
398.2021	3.587992	1.92E-13	1.67E-12	4399025.153	7288580.85	16308.72766	2126021.404
233.59724	3.562283	1.80E-13	1.57E-12	525423.1501	1335088.488	6057.516529	562842.2054
403.20925	3.16105	1.79E-13	1.57E-12	9384212.598	11745684.59	331484.0748	5172824.925
222.60662	3.1344	1.72E-13	1.51E-12	2716885.228	12451555.95	97350.34295	1059597.308
189.05279	2.994483	1.68E-13	1.48E-12	1112056.055	839518.5353	689773.3155	171026.7072
458.16252	3.518833	1.66E-13	1.47E-12	1437143.042	2857810.807	4465.546864	1010925.022

Supplementary A

Kruskal-Wallis significant metabolites positive ionization mode

223.0965	2.933625	1.60E-13	1.41E-12	1309793.126	977774.3062	15037009.18	1342456.506
291.06301	3.082117	1.59E-13	1.41E-12	4141478.569	2382179.882	144532.6849	1005538.377
212.12816	3.309	1.59E-13	1.41E-12	7964714.955	699223.2539	1451593.702	6500986.153
433.16484	3.073383	1.59E-13	1.41E-12	2911490.12	1633519.863	50922.4327	2377556.932
246.11251	2.905167	1.49E-13	1.32E-12	405077.6365	164343.5811	76377.55434	828791.6605
363.21664	3.5269	1.41E-13	1.26E-12	65246058.26	115680623	505042.0324	48015497.36
351.24417	3.630867	1.39E-13	1.24E-12	4512789.64	8311726.617	222524.8945	4471963.152
206.04693	3.544567	1.33E-13	1.19E-12	951047.2877	810113.2822	89788.48189	203506.0101
293.09345	2.977425	1.30E-13	1.17E-12	1573226.661	622052.8575	78701.61095	621187.6968
397.19872	3.582617	1.30E-13	1.17E-12	16326315.38	32083972.8	155064.4358	9301055.066
405.20384	3.22195	1.24E-13	1.12E-12	1373595.17	563042.9931	38075.25106	998324.0205
324.06507	2.836717	1.23E-13	1.11E-12	605171.6968	56180.60484	77798.63085	582825.2707
236.13621	3.748883	1.20E-13	1.09E-12	430446.6285	340867.7147	307387754.4	252940.6758
589.15102	3.357333	1.19E-13	1.08E-12	1739983.365	329184.0449	5708.846599	3798.988064
319.08129	2.599142	1.15E-13	1.05E-12	3845364.042	2836960.527	925378.4475	483778.3258
211.0488	3.11705	1.11E-13	1.01E-12	1591889.44	1791017.555	38895.40702	70613.31247
145.9855	2.44355	1.11E-13	1.01E-12	4747096.127	1351456.491	1274351.812	3455080.015
330.21452	3.160617	1.09E-13	9.96E-13	75851219.36	123992660	1295395.619	36349401.6
367.26322	3.815033	1.08E-13	9.90E-13	718191.4639	1080439.173	7679.265299	286368.6573
281.0809	2.829217	1.06E-13	9.74E-13	1776086.94	494658.8658	403163.1071	1354145.978
350.24073	3.233725	1.02E-13	9.43E-13	48191757.95	78397170.06	327441.0433	31268330.39
602.30021	3.22515	1.01E-13	9.37E-13	889072.8575	5596.05353	3691.859611	434750.578
349.23742	3.63165	1.00E-13	9.26E-13	237486361.6	417834160.6	2073260.397	184862123
447.14661	3.562	1.00E-13	9.26E-13	2098598.593	3967159.308	13487.72747	1302965.76
387.21428	3.115867	9.93E-14	9.24E-13	291342490.6	356546161.7	3430248.846	123834947.8
218.05661	2.98625	9.83E-14	9.17E-13	2372601.972	1074994.825	993391.1515	98271.07969
386.20194	3.524692	9.59E-14	8.97E-13	10333197.22	16304763.66	31070.23499	6974449.567
349.2374	3.231542	9.43E-14	8.83E-13	212364961.4	341112231.8	1714993.261	139998189.7
377.23233	3.639783	9.26E-14	8.69E-13	16654851.84	37764938.69	215150.7661	10164972.46
346.20948	3.317267	9.07E-14	8.54E-13	1974643.203	3160849.58	36794.45778	1220654.716
442.1901	3.518417	8.53E-14	8.04E-13	501885.8407	1161753.101	4161.536164	398656.9534
217.08604	3.082233	8.51E-14	8.04E-13	5742973.7	667315.6422	681419.9734	4971049.089
216.12316	2.55635	8.44E-14	8.00E-13	3859573.402	357746.9217	199737.0699	9033364.898
350.24074	3.637908	8.42E-14	8.00E-13	54144173.4	95906312.18	489821.8255	42756122.88

Supplementary A

Kruskal-Wallis significant metabolites positive ionization mode

255.63569	3.237525	8.18E-14	7.78E-13	1135805.145	2994782.306	13646.04507	767239.6746
388.21763	3.1162	8.17E-14	7.78E-13	64549871.75	81742004.46	835233.6413	29043125.52
253.60879	3.56595	7.93E-14	7.58E-13	373845.9939	1071837.789	3835.800142	388138.6911
243.11997	3.1344	7.81E-14	7.49E-13	4271285.651	14600228.85	246418.9339	2836205.649
385.19854	3.518442	7.55E-14	7.25E-13	47879910.22	73665449.8	146061.544	29343296.96
212.07569	2.829133	7.52E-14	7.24E-13	1204974.816	208326.187	263065.0679	890052.3618
266.11037	3.04465	7.27E-14	7.02E-13	287238.7063	313938.602	118534521	189287.3258
349.23737	3.837408	7.19E-14	6.96E-13	28042210.65	48511569.12	735355.8426	19018005.57
371.21939	3.631717	7.20E-14	6.96E-13	94863770	157230450.2	741163.4885	76556287.98
329.21117	3.148583	6.95E-14	6.76E-13	341212634.8	545469745.2	2673864.08	166607953.9
366.19546	3.56165	6.69E-14	6.52E-13	1269331.371	4352602.435	15872.05208	791725.5474
409.0588	2.98625	6.58E-14	6.43E-13	1698232.399	588603.9903	251649.9422	147210.4166
123.53631	1.903233	6.36E-14	6.23E-13	855204.9046	343573.1664	348599.5955	571802.1706
273.02946	3.544467	6.37E-14	6.23E-13	1163185.061	401358.9907	37588.12695	113561.3999
366.23579	3.553117	6.10E-14	6.00E-13	2618056.112	9855434.733	105878.7637	1866224.51
253.07065	2.082267	6.03E-14	5.95E-13	1053247.046	790402.9627	161429.0314	156983.655
386.2019	3.180742	5.99E-14	5.91E-13	3140232.413	4504382.319	19045.67423	1892163.813
309.10258	2.5991	5.80E-14	5.75E-13	5672046.467	816347.703	798757.5467	4286954.393
405.22716	3.4829	5.73E-14	5.69E-13	8280597.879	970220.2153	49864.02684	6122055.598
372.22272	3.63165	5.14E-14	5.12E-13	21048454.39	35680394.7	120533.6098	17155211
383.18304	3.125942	4.79E-14	4.78E-13	16297042.2	17225355.64	135939.2626	7629507.631
405.16017	2.837117	4.72E-14	4.72E-13	11394287.34	41553646.66	496415.2271	4777550.284
185.10021	2.837358	4.67E-14	4.68E-13	7888610.961	522632.7822	541027.9968	6662949.391
401.19361	3.090867	4.43E-14	4.45E-13	15184619.29	14625581.84	397324.01	5521597.359
253.14346	3.055758	3.63E-14	3.66E-13	217811.1174	358848.9056	39914256.68	241210.1147
369.20373	3.169842	3.41E-14	3.44E-13	77220102.06	100170736.6	993405.9239	38261392.22
251.12785	3.012283	3.34E-14	3.38E-13	539754.8056	689874.6976	431702105	425858.6233
406.16349	3.418017	3.27E-14	3.32E-13	8448787.186	342862.3053	260104.4464	5830672.876
218.10237	2.424867	3.19E-14	3.24E-13	3868233.46	274949.1537	359972.2074	2798755.508
385.19857	3.178333	3.13E-14	3.19E-13	14910085.04	23480965.63	155357.9465	10296974.41
376.07719	2.986717	3.11E-14	3.18E-13	1216106.459	493839.3003	2146836.158	64282.85426
312.05626	2.977667	3.00E-14	3.08E-13	689727.1586	541833.0565	162169.9304	98762.60965
385.27377	3.4622	2.96E-14	3.04E-13	4785771.357	4952197.116	12708.83729	1590484.282
236.0588	3.0736	2.91E-14	2.99E-13	2114100.56	231751.3237	332983.7988	1814043.562

Kruskal-Wallis significant metabolites positive ionization mode

373.2262	3.630367	2.75E-14	2.84E-13	1853921.999	3324374.76	44602.47123	1786413.761
135.08052	3.06895	2.70E-14	2.79E-13	1777172.673	517367.4604	566866.5121	1441013.133
205.52952	3.559983	2.68E-14	2.78E-13	877695.5539	540120.3684	11041.54322	252846.8769
261.06725	3.1172	2.50E-14	2.60E-13	1537628.083	966518.3755	147126.0324	567087.4553
370.20707	3.174683	2.49E-14	2.59E-13	16767457.61	22093296.01	235649.5042	8578862.577
363.21659	3.169233	2.35E-14	2.46E-13	56775830.05	104137426.3	696824.1789	36822075.94
330.21451	3.562067	2.34E-14	2.45E-13	80506295.02	148646586.2	351475.4047	50124398.99
139.03903	3.098717	2.25E-14	2.36E-13	2668406.11	16007703.83	20953824.21	1917914.756
329.21119	3.561992	2.14E-14	2.25E-13	362171502.1	653754459.7	1813324.157	224510959.1
170.08118	2.618408	2.01E-14	2.12E-13	4886357.419	1106256.511	629692.1257	3800126.683
297.07578	2.424358	1.88E-14	1.99E-13	1420286.279	152411.7671	193900.5705	1018779.154
345.1235	2.837367	1.88E-14	1.99E-13	1831483.458	37188.49634	86846.65175	1551880.797
625.25081	3.422567	1.87E-14	1.99E-13	632257.2453	10892.09846	5584.438824	447508.8596
347.22179	3.56195	1.74E-14	1.86E-13	115630557.4	237196648.4	972827.9196	75659308
385.27361	3.814817	1.72E-14	1.84E-13	1287198.83	2423321.273	5475.629928	595262.7803
181.10522	3.0733	1.71E-14	1.84E-13	2730021.369	548416.9727	708968.9802	2603086.919
431.17432	3.1773	1.62E-14	1.74E-13	925324.8152	3054905.367	38132.02485	856823.323
125.05988	3.0648	1.55E-14	1.67E-13	1656444.634	740758.0177	888860.8904	1345703.986
766.39359	3.570233	1.52E-14	1.65E-13	638361.183	25074.44671	1588.391399	351894.2603
225.54107	3.553533	1.48E-14	1.60E-13	7965852.998	4641657.849	132760.5685	2102978.894
348.22511	3.561983	1.48E-14	1.60E-13	25501461.55	52649909.97	118806.1437	15768579.96
235.09658	2.652317	1.41E-14	1.54E-13	28244046.79	13555476.98	1325473.327	3431798.538
235.6241	3.230858	1.37E-14	1.49E-13	631318.2964	1571350.093	5484.726522	361477.9965
327.06388	2.837075	1.32E-14	1.45E-13	630931.4078	58779.97864	94965.43047	467694.9795
536.25501	2.615358	1.33E-14	1.45E-13	315720.9599	5853.939137	34256.73538	243320.3391
280.08969	2.721333	1.21E-14	1.33E-13	1132819.608	452584.8523	126032.5782	77424.97433
237.13948	3.758267	1.19E-14	1.31E-13	364991.5914	403519.9499	12394920.35	263251.2229
582.2643	3.484858	1.11E-14	1.23E-13	783781.3406	3442.377223	3118.116883	422869.7813
288.11932	2.83685	1.02E-14	1.13E-13	5316979.207	105088.6476	536445.9484	4096775.515
319.93772	2.43325	9.93E-15	1.10E-13	396522.4206	18206.75892	7716.171595	259904.5694
354.00601	3.117192	9.73E-15	1.09E-13	1202319.874	1266529.534	3051.87346	52433.90894
226.05398	3.278683	9.43E-15	1.05E-13	470018.9031	343779.3766	50509.76811	79596.77199
584.30711	3.13435	9.41E-15	1.05E-13	911968.3861	13788.3995	3126.190838	518239.0569
251.04378	1.904325	8.68E-15	9.76E-14	526514.5075	72456.50694	157409.6241	385189.7668

Kruskal-Wallis significant metabolites positive ionization mode

1064.4061	3.527567	8.10E-15	9.12E-14	344854.6407	1792.262261	1446.290935	126344.3078
135.04414	1.904242	7.96E-15	9.00E-14	2562815.612	1201555.594	916856.62	1884498.823
280.90933	2.410267	7.94E-15	8.99E-14	597320.5661	32759.32801	16399.34914	454774.425
290.07849	2.986317	7.10E-15	8.07E-14	1290649.931	569819.724	304451.5114	101568.773
364.07706	2.717567	6.38E-15	7.27E-14	530169.9602	219702.5728	41627.64389	44011.42454
598.2464	3.176167	6.38E-15	7.27E-14	1485956.735	6304.719472	12583.11088	539277.6671
391.14437	2.445642	6.17E-15	7.07E-14	840790.0985	17733.74513	29634.43397	611302.1321
448.08933	3.117333	6.13E-15	7.04E-14	8954079.544	2301582.821	15171.40081	4572690.513
370.20709	3.561933	6.11E-15	7.03E-14	44089219.19	78711339.29	218236.6588	29250309.94
369.20375	3.561833	5.71E-15	6.60E-14	197550888.3	348278227.1	1269244.798	130219606.2
607.26545	3.5184	5.41E-15	6.26E-14	536618.3485	5227.380749	2203.646098	287865.8132
295.09662	2.5501	5.30E-15	6.16E-14	1507709.406	501004.8426	354035.6978	2791751.614
204.0325	1.911967	4.98E-15	5.80E-14	864838.9253	226848.8963	86495.69323	592914.5675
489.15585	3.407217	4.36E-15	5.09E-14	1001025.839	52020.35962	31546.58429	490739.365
618.27262	3.227542	4.14E-15	4.85E-14	515449.1962	4667.356021	2509.651124	208000.2434
635.08783	3.5444	4.10E-15	4.82E-14	390679.4016	198000.6673	1617.815681	23068.28751
447.14661	3.177883	3.99E-15	4.70E-14	5636051.117	3159667.327	23984.9607	892311.1244
113.51772	2.419	3.92E-15	4.63E-14	4762910.866	391817.2213	355812.3475	2852197.977
147.98366	2.438167	3.86E-15	4.58E-14	1931001.288	618732.8731	538305.6167	1377633.806
320.11952	3.073283	3.58E-15	4.25E-14	1648411.704	216590.2689	149595.5741	1272564.126
201.1199	3.073475	3.25E-15	3.87E-14	2689720.188	299514.3905	286824.5705	2059151.365
189.9798	2.330058	3.24E-15	3.87E-14	7913491.488	16990331.83	16067833.82	7101529.995
234.61631	3.17285	3.14E-15	3.76E-14	908668.9527	2649620.199	16394.71129	416575.5612
569.28854	3.562017	2.99E-15	3.59E-14	756530.3404	5079.529432	1926.730575	499963.6489
254.62789	3.178117	2.91E-15	3.51E-14	1056337.14	3371813.104	6877.925915	603582.6163
351.22789	3.599067	2.80E-15	3.38E-14	2294734.884	8270356.23	201566.8397	1888229.384
251.1173	3.15235	2.73E-15	3.31E-14	365663.3728	995590.393	42745683.73	264415.0144
221.03397	2.763817	2.69E-15	3.27E-14	2520506.495	563364.9263	14042.5236	980933.1513
765.39021	3.570383	2.64E-15	3.22E-14	1839399.562	146741.0596	1703.577885	715308.6377
599.25408	3.622933	2.56E-15	3.13E-14	267939.0064	2730.063277	1835.991764	116357.7753
571.30419	3.619325	2.52E-15	3.09E-14	580404.4094	3341.736265	2354.17072	328525.7912
282.11101	2.044133	2.20E-15	2.71E-14	2722461.778	256935.5492	356999.4429	1869331.942
691.46828	4.726483	2.13E-15	2.63E-14	2042666.5	21857.1824	13609.62827	1373163.772
289.07065	3.28275	2.00E-15	2.47E-14	6138001.293	2960852.463	344516.6568	267244.8986

Kruskal-Wallis significant metabolites positive ionization mode

182.08941	3.064717	1.73E-15	2.14E-14	1302131.618	68281.76965	126220.4632	961258.3516
321.06056	2.8457	1.72E-15	2.14E-14	11449126.43	4384476.086	361191.1905	1956033.865
434.11358	3.432775	1.71E-15	2.13E-14	351985.0187	2510.983773	3666.082852	182994.6757
449.08179	3.11725	1.65E-15	2.07E-14	2149080.247	320969.5069	4741.706731	1206862.721
460.17149	2.613867	1.57E-15	1.97E-14	525861.1578	20102.12995	79746.71745	356744.7481
420.09787	2.829325	1.55E-15	1.96E-14	1334990.982	56329.35351	28801.87693	674833.0739
347.13288	2.985983	1.54E-15	1.95E-14	1101655.537	854928.5918	98360.22189	157628.806
585.28343	3.517567	1.43E-15	1.81E-14	1070410.174	3261.016053	4504.788793	566687.2421
456.10259	2.977508	1.40E-15	1.78E-14	474280.934	136133.2057	2743.612318	3153.084835
631.3004	2.634667	1.40E-15	1.78E-14	1036258.319	1685.305651	2396.857612	338783.1062
181.08592	3.07335	1.34E-15	1.71E-14	15363528.47	861569.785	4346089.913	11428554.48
617.26037	3.134667	1.31E-15	1.68E-14	1481678.13	13337.27411	2124.300902	523226.864
226.14378	3.514917	1.22E-15	1.57E-14	945610.857	192416.8542	27352.04981	617172.5844
202.55832	3.119042	1.21E-15	1.56E-14	770286.1385	1527145.343	38608.35771	176382.9474
406.16348	2.5279	1.18E-15	1.53E-14	5504819.361	652607.3436	377845.5495	4092341.63
156.10199	3.073517	1.15E-15	1.49E-14	5237002.501	1425034.371	1295611.695	3892030.644
304.05328	2.75795	1.05E-15	1.37E-14	3979930.441	1069419.642	147851.4036	12854352.02
169.04953	2.176733	1.05E-15	1.37E-14	6584684.651	3808902.423	960684.3233	910424.4909
422.18364	3.523133	1.04E-15	1.37E-14	1196040.99	24979.25962	41452.97318	870093.161
265.03313	2.977667	9.93E-16	1.31E-14	1390590.471	552076.4316	307839.2393	250773.1049
411.04846	3.544583	9.89E-16	1.31E-14	2428028.605	1422896.531	3537.775965	317589.5209
146.5521	1.912367	8.88E-16	1.18E-14	1438079.551	324976.0431	278097.7024	1096827.83
245.62264	3.176425	8.83E-16	1.17E-14	829640.7223	2009314.381	9530.370284	640369.5835
139.07534	3.0734	8.69E-16	1.16E-14	2265505.208	664274.5017	781090.5308	1833024.868
448.14987	3.56225	8.59E-16	1.15E-14	7052426.057	725235.3397	8958.233794	2775209.903
450.05145	3.544617	8.59E-16	1.15E-14	1578567.585	525322.5836	4078.93043	589834.7433
480.12368	3.116608	8.58E-16	1.15E-14	932398.9395	8430.968487	40505.71956	41701.01478
185.53348	2.986217	8.47E-16	1.14E-14	1044367.314	360512.6469	10216.90309	34949.96364
329.12254	3.553267	8.28E-16	1.12E-14	973967.0884	1952380.907	11772.463	250465.3836
418.19025	3.0735	7.75E-16	1.05E-14	2701544.917	824220.0033	20427.70736	1762764.156
278.91121	2.318342	7.64E-16	1.04E-14	2192009.284	261923.298	152586.0574	1862081.414
416.06535	2.986083	7.58E-16	1.04E-14	4710493.356	1331156.351	416789.2435	64475.13424
413.04413	3.545058	7.44E-16	1.02E-14	1008186.134	460535.7811	1877.599272	153162.3284
767.40601	3.619592	7.33E-16	1.01E-14	587513.8132	3230.197186	1467.373083	301721.6575

Kruskal-Wallis significant metabolites positive ionization mode

210.54712	3.124225	7.12E-16	9.83E-15	10762381.97	9211662.425	61163.82084	691846.4505
673.20947	3.553283	7.12E-16	9.83E-15	1138627.063	1043388.75	1387.648122	112795.2744
191.97794	2.336817	6.89E-16	9.58E-15	2283317.561	184194.3288	168371.0566	1750770.56
300.05308	3.073492	6.82E-16	9.51E-15	878395.2398	55876.75737	39512.58393	894036.8988
218.05662	3.544583	6.62E-16	9.26E-15	3055208.643	2441182.167	71528.11017	668729.5348
298.54702	3.108483	6.31E-16	8.86E-15	783618.8086	746957.4843	1285.906517	8804.771735
598.24631	3.562167	5.71E-16	8.04E-15	1191276.558	6582.543504	2166.327219	610571.5282
303.13858	3.08205	5.55E-16	7.85E-15	2015038.861	204391.2444	190295.7555	1279963.86
137.5468	1.912833	5.46E-16	7.75E-15	560987.9828	113174.2698	100129.4577	379274.2045
237.59937	3.090275	5.34E-16	7.60E-15	29850115.17	656449.2733	86704.79868	26118678.56
360.14262	3.082283	5.33E-16	7.60E-15	1997767.556	78835.63745	356687.195	1726235.047
584.28005	3.518242	5.09E-16	7.30E-15	3013097.832	5337.744774	8167.828158	1663762.867
349.63562	3.5533	4.86E-16	6.98E-15	456875.0842	458944.1453	1667.617973	35741.3481
243.07986	3.064767	4.83E-16	6.98E-15	2884747.17	10290493.12	91654.63237	2207060.682
221.02351	3.117075	4.78E-16	6.93E-15	2518980.405	144242.2824	46076.1721	2288440.94
564.16128	2.613675	4.60E-16	6.69E-15	399880.2478	2431.781862	5789.735104	249741.8991
233.03326	1.904225	4.52E-16	6.60E-15	3567405.772	524646.0488	647620.5214	2300547.933
298.05987	2.716167	4.46E-16	6.52E-15	3599948.392	744289.3993	75474.62669	105437.3407
270.14169	2.75855	3.95E-16	5.80E-15	207168.7714	55961.40731	10215022.84	237140.1792
368.02171	3.544942	3.86E-16	5.69E-15	11271747.79	5847192.065	38760.02867	1611566.881
601.28815	3.117067	3.74E-16	5.54E-15	2690357.749	33283.49681	5301.398203	1224635.151
292.06546	2.837967	3.69E-16	5.47E-15	826398.1635	40449.39058	53149.69224	38959.88441
433.07013	3.0298	3.69E-16	5.47E-15	6110032.963	925672.688	131541.0359	377546.0124
395.0327	3.124667	3.34E-16	4.99E-15	6286573.118	5317076.725	20802.77486	351681.9968
214.10734	2.704933	3.25E-16	4.89E-15	3386227.199	1103491.337	1108289.431	3010240.644
331.07482	2.977933	3.26E-16	4.89E-15	1299891.702	475614.6255	47683.48262	38946.38976
186.03812	2.7207	3.21E-16	4.85E-15	561746.7781	352976.7159	123741.9812	122586.9382
570.30078	3.614333	3.02E-16	4.58E-15	1919594.921	6939.152878	1749.729665	931947.537
410.05168	3.544583	2.86E-16	4.35E-15	7344757.381	3272078.876	19749.92167	1109740.61
272.06821	3.544583	2.83E-16	4.32E-15	1414335.56	854005.4531	13207.29134	214012.8612
405.16014	2.5286	2.73E-16	4.18E-15	28528673.63	3561967.188	799886.8134	19772924.11
236.06349	2.512192	2.64E-16	4.06E-15	10841151.65	5884469.267	616753.3591	601626.8073
338.05603	3.134042	2.58E-16	3.99E-15	3621109.677	395222.4145	235197.8277	3266971.726
393.07575	3.54455	2.57E-16	3.99E-15	1442710.581	1119058.074	14150.37009	198673.9512

Kruskal-Wallis significant metabolites positive ionization mode

603.26889	3.421933	2.52E-16	3.92E-15	4030005.064	12588.74558	6141.616866	2878521.902
234.08424	2.731783	2.48E-16	3.88E-15	9224210.394	4896635.838	462039.247	473915.6262
238.06821	3.544592	2.47E-16	3.87E-15	2409997.477	1859480.649	30177.00403	554376.284
111.08079	3.0648	2.39E-16	3.77E-15	867344.9656	279900.9661	298257.2704	680363.3196
233.53853	3.279183	2.38E-16	3.77E-15	489986.1167	150290.0579	1734.953858	13638.67626
262.04998	2.837117	2.39E-16	3.77E-15	4798443.788	697414.7868	274727.7217	3882472.625
336.08524	3.0735	2.39E-16	3.77E-15	16324116.67	1719309.194	872049.2413	12215775.26
616.25716	3.125833	2.13E-16	3.41E-15	5256280.368	145420.023	3384.393292	2376737.833
196.09694	3.168958	1.99E-16	3.19E-15	6289562.599	324567.9877	304663.8684	5003588.682
487.16317	2.616517	1.90E-16	3.06E-15	611989.092	9611.87577	28381.97473	423785.181
407.05302	3.544983	1.83E-16	2.96E-15	2079551.252	1499836.899	13278.63631	333663.8506
220.04439	2.593842	1.78E-16	2.89E-15	526774.3002	285232.5715	11747.52889	15517.76155
429.0745	2.873008	1.77E-16	2.88E-15	647401.3186	153812.2392	10553.01104	13742.34637
409.04828	3.544725	1.72E-16	2.81E-15	39222305.09	21041545.88	175103.384	6697438.457
434.07355	3.0218	1.63E-16	2.68E-15	1085514.762	181419.7927	11929.79715	108790.6244
199.11571	3.082	1.53E-16	2.53E-15	103580096.6	3041584.65	1353632.69	82891070.52
209.56613	3.553117	1.49E-16	2.47E-15	1443608.903	1240329.319	16843.31122	379840.6099
268.0493	2.836717	1.42E-16	2.36E-15	450675.1098	8718.27123	13047.03398	401990.6237
223.58367	2.829342	1.40E-16	2.33E-15	3359993.397	126756.3094	21660.1911	2525265.082
1063.403	3.527567	1.29E-16	2.16E-15	792827.3669	1473.403556	1595.448608	253194.2022
377.09828	3.544533	1.28E-16	2.16E-15	1984270.667	1184294.164	9090.06888	191461.0026
238.1011	3.090967	1.20E-16	2.02E-15	6025438.736	177830.1176	117312.2504	5178121.664
600.26208	3.623933	1.19E-16	2.02E-15	626258.0541	4074.603417	2918.458105	322720.9312
286.08648	2.00975	1.18E-16	2.01E-15	2101669.159	105733.991	133611.4119	1557838.834
370.05991	2.986083	1.12E-16	1.91E-15	814249.2412	378446.6209	60579.40912	36450.42077
358.05978	2.722508	9.94E-17	1.71E-15	2252326.443	643705.1438	47850.85076	134803.722
689.21409	3.529417	9.81E-17	1.69E-15	680020.4548	12094.21017	1550.101541	458863.0225
417.18741	3.07345	9.48E-17	1.64E-15	15074573.56	2441388.495	126623.8206	9369173.937
274.05972	1.912683	9.24E-17	1.61E-15	892432.9901	241852.0194	182133.1353	601902.6307
359.13922	3.082192	9.05E-17	1.58E-15	14176437.15	775377.2777	473488.9667	11899482.78
455.09945	2.977608	8.51E-17	1.49E-15	3818414.091	1111581.565	18474.4048	14108.33857
428.58534	3.542442	8.44E-17	1.48E-15	1134779.566	416631.9147	1362.272152	39700.1673
200.11654	3.073467	8.31E-17	1.47E-15	7350538.746	303563.7359	243988.6229	9495490.454
542.0318	3.117267	8.27E-17	1.47E-15	1011686.634	196442.1671	1800.335777	7290.808414

Kruskal-Wallis significant metabolites positive ionization mode

217.55495	3.544583	7.15E-17	1.27E-15	16120174	10970030.41	101269.7851	3361213.165
568.28514	3.561958	7.12E-17	1.27E-15	2855079.748	4881.278751	2056.316115	1521120.95
1036.4561	3.518392	6.98E-17	1.26E-15	1467135.87	1615.686532	1285.906517	636000.4738
448.59687	3.542517	6.88E-17	1.24E-15	675133.3912	252655.0197	1360.586351	30197.41687
282.06492	3.07345	6.60E-17	1.20E-15	2694742.723	93840.28297	153611.4062	2103590.166
227.56795	2.880867	6.51E-17	1.18E-15	1944456.034	488647.1138	28613.2315	73549.11288
445.07017	3.143392	6.06E-17	1.11E-15	4193531.04	1025329.654	43366.72721	85169.66823
276.08306	3.544517	6.03E-17	1.11E-15	1300208.64	442276.5346	4274.64439	102240.5819
446.1339	3.535983	5.92E-17	1.09E-15	1907267.053	84083.6101	2680.494192	785531.604
725.21577	3.081883	5.79E-17	1.07E-15	581461.9281	6015.174759	2228.873352	942847.5394
235.06018	2.515233	5.71E-17	1.06E-15	91761309.52	40156522.88	1486369.644	4488474.155
280.04613	2.83685	5.59E-17	1.05E-15	395500.5295	7168.66161	3286.208951	265250.2361
172.0968	2.4236	5.25E-17	9.86E-16	6673755.404	611961.3841	535441.862	4356496.574
1021.4373	3.501383	4.71E-17	8.89E-16	380420.2782	1285.906517	1420.78113	165797.2093
333.57755	3.544483	4.66E-17	8.83E-16	1878864.981	788002.4578	1439.218518	116847.891
369.02529	3.553075	4.60E-17	8.76E-16	1920409.807	789157.4094	1977.671837	229704.4684
428.08367	3.544433	4.55E-17	8.71E-16	2473701.669	924135.9632	2137.317333	83080.6081
218.57141	2.988067	4.51E-17	8.67E-16	1519393.141	681486.0173	189377.3612	50301.91557
193.57874	2.837217	4.38E-17	8.47E-16	1589160.91	24804.17316	111107.986	1409570.352
1020.4341	3.501342	4.39E-17	8.47E-16	808873.2801	1472.302835	1372.081671	320864.8319
187.03906	2.510483	4.25E-17	8.28E-16	1917268.228	1826736.892	417485.6634	444967.9284
697.32758	2.61145	4.23E-17	8.28E-16	1238152.81	1764.015577	2329.878699	608836.1321
412.08972	2.985217	4.05E-17	7.96E-16	1196954.63	365305.1027	10170.73774	21148.15836
562.16553	2.613783	3.78E-17	7.47E-16	1251679.218	4159.134055	7769.15005	775209.8984
360.03902	2.506092	3.73E-17	7.41E-16	821838.6169	365007.7552	9259.844684	12336.42649
269.13839	2.751183	3.42E-17	6.82E-16	2086267.542	416294.699	71298195.12	1591614.532
324.60959	2.9865	3.30E-17	6.62E-16	502236.2015	186339.8222	5403.691276	8195.318326
300.64605	3.108525	3.22E-17	6.48E-16	2700578.776	158405.3818	4267.494084	1504849.8
173.06548	2.837942	3.16E-17	6.39E-16	18319685.74	648194.503	867631.8803	16705340.33
883.34966	2.609125	3.08E-17	6.26E-16	568121.8857	1405.399286	1544.681089	218271.556
583.27733	3.175917	3.05E-17	6.24E-16	489165.33	8249.629285	1624.586471	532158.5164
200.0469	2.722708	3.02E-17	6.20E-16	9864800.362	5731457.883	2283334.127	1624343.687
214.10734	2.462875	2.89E-17	5.96E-16	3936238.372	789411.5944	791165.6485	4066483.685
455.60107	2.977525	2.82E-17	5.86E-16	1795014.023	193668.5688	3462.081388	1931.893057

Supplementary A

Kruskal-Wallis significant metabolites positive ionization mode

121.96639	0.312196	2.79E-17	5.84E-16	2488569.201	4158465.481	4384851.356	2499264.584
600.28474	3.117083	2.80E-17	5.84E-16	9210884.607	357178.7566	2410.731101	3729188.701
606.26193	3.518525	2.79E-17	5.84E-16	1762369.511	2855.449376	2623.576402	891588.4447
312.56255	3.544475	2.69E-17	5.69E-16	5169765.301	2190275.794	1933.86437	278434.1103
630.29687	2.634067	2.69E-17	5.69E-16	3635571.801	2044.998225	2552.147716	1035865.598
227.06182	2.986217	2.51E-17	5.36E-16	4018696.761	1917645.218	705226.1054	295523.5928
489.15597	3.0735	2.49E-17	5.34E-16	1317339.48	33388.82721	22631.47019	912561.0431
459.16829	2.616825	2.39E-17	5.17E-16	2616799.004	34580.86637	99969.65304	1767385.633
700.23331	3.518283	2.35E-17	5.10E-16	781438.5489	2769.631904	2731.802227	444306.7706
624.2473	3.423708	2.33E-17	5.10E-16	2161292.12	10431.31011	1382.069287	1121126.721
169.55485	2.427925	2.28E-17	5.00E-16	3527575.092	199269.5279	105289.9699	2429460.995
233.08091	2.73305	2.24E-17	4.95E-16	74965222.77	28195278.09	1153970.099	2200431.818
397.07089	2.548642	2.12E-17	4.71E-16	913649.4838	570712.9341	63013.52915	99104.76457
286.03735	2.836717	2.05E-17	4.56E-16	469023.3823	2794.793538	4437.321292	378899.9614
219.5524	2.54725	2.01E-17	4.50E-16	1232820.417	685325.6876	70529.90034	152015.7717
283.10325	2.0877	1.99E-17	4.48E-16	3648967.864	708860.9894	530825.0854	2629781.673
160.06163	3.117125	1.91E-17	4.33E-16	1764656.874	145629.0061	120841.3457	1851628.257
582.27403	3.169883	1.91E-17	4.33E-16	1787917.611	23794.44342	2241.367824	1634501.684
178.03376	2.506408	1.82E-17	4.17E-16	3342020.556	1804726.271	79230.5691	144453.9275
332.05877	3.16055	1.73E-17	3.99E-16	6928390.529	1290313.706	44827.5827	1856798.474
259.06025	3.12415	1.64E-17	3.79E-16	158139883.6	88563135.84	760758.4932	15836994.38
233.1173	3.019533	1.62E-17	3.78E-16	874024.7778	1175531.712	44282943.91	263215.9357
405.16016	3.420683	1.50E-17	3.52E-16	42419513.27	1099229.897	109590.0305	28137294.87
411.08644	2.986083	1.40E-17	3.31E-16	6292055.023	2163094.443	53254.31639	167674.8556
262.07914	2.721633	1.38E-17	3.27E-16	4726348.058	1524414.069	170611.6438	308682.9569
260.06359	3.119492	1.37E-17	3.25E-16	22393168.09	14310021.9	78973.45406	1836522.311
602.26534	3.42155	1.26E-17	3.02E-16	12248889.25	5687.698063	4074.295495	9238505.463
273.07575	2.986008	1.23E-17	2.97E-16	262835194.1	76415415.29	4813379.994	3001407.92
281.10769	2.593892	1.22E-17	2.96E-16	14796807.91	537604.5462	874187.3117	9722343.978
309.10246	2.067	1.19E-17	2.89E-16	7946233.135	374979.5625	475119.9178	5983904.103
468.06214	2.98615	1.18E-17	2.89E-16	1387635.821	314187.748	10703.26853	13717.95925
319.11588	3.073383	1.17E-17	2.88E-16	38959928.41	1833987.209	1580501.236	32035152.81
395.10894	2.985983	1.16E-17	2.88E-16	3228594.134	1143695.677	49470.35812	41264.99057
332.08959	3.073533	1.15E-17	2.88E-16	10905147.03	1278511.031	170444.2671	8063581.049

Kruskal-Wallis significant metabolites positive ionization mode

504.99993	3.70255	1.15E-17	2.88E-16	654842.0575	97862.83494	1806.897416	8657.561263
215.55392	2.986642	1.14E-17	2.88E-16	727117.7595	288120.7919	66736.51007	34444.13044
274.07908	3.544617	1.11E-17	2.88E-16	223156768.5	103190914.9	402689.4693	19291140.48
281.10762	2.069933	1.14E-17	2.88E-16	26059752.75	1096507.721	652724.5717	18976539.17
304.09689	2.837967	1.12E-17	2.88E-16	41074453.14	1197787.282	243052.2848	34762642.63
856.35809	2.609883	1.13E-17	2.88E-16	1562340.393	1574.768199	1823.154117	545419.9543
342.07916	3.090083	1.09E-17	2.84E-16	1281151.752	99994.04272	25001.42413	1065286.103
226.09145	3.09125	9.95E-18	2.62E-16	3804144.397	408749.4832	239057.8278	2923831.104
242.04889	2.760417	9.97E-18	2.62E-16	850517.9192	263500.6588	15785.203	36753.098
197.0416	2.986167	9.29E-18	2.47E-16	4549303.591	2511703.92	307149.3449	363399.9741
274.07909	2.985983	9.20E-18	2.46E-16	41844404.67	11209359.79	1773763.059	498536.1385
468.08541	2.986175	9.15E-18	2.46E-16	1490834.895	334241.9366	10702.92731	13703.34531
273.07577	3.547083	8.90E-18	2.41E-16	1357610682	642749987.5	2773084.854	126641777.9
161.55741	2.837133	8.49E-18	2.31E-16	5743894.553	78371.02809	85045.1899	4924984.478
303.04996	2.759675	8.42E-18	2.31E-16	24955685.52	6287160.105	550483.4243	797911.5826
173.56714	2.83735	8.25E-18	2.28E-16	2329598.774	55629.61737	40011.97817	2239165.621
598.05581	3.544417	8.13E-18	2.26E-16	2142001.756	454254.7623	1373.057178	63258.17693
855.35474	2.6106	8.08E-18	2.26E-16	3589887.591	2155.276514	1628.524741	1248287.036
688.21076	3.527025	7.96E-18	2.24E-16	2335928.197	6393.037799	1548.233171	1190964.372
245.52771	2.8891	7.70E-18	2.19E-16	696550.7904	154178.7619	6303.016122	17698.42337
449.13261	3.535983	7.72E-18	2.19E-16	8208112.298	445514.3682	13239.56707	3680176.922
338.08079	3.073567	7.29E-18	2.10E-16	5721517.942	403642.7427	194046.6408	4680429.415
180.5749	3.11715	6.72E-18	1.95E-16	8116338.503	268522.2513	133280.1091	7780415.328
214.03305	2.986767	6.50E-18	1.90E-16	5109099.664	3380051.71	728539.5225	765508.1566
278.08929	3.0734	6.46E-18	1.90E-16	1947458.07	139736.0708	151366.3832	1548774.365
399.08645	2.721475	6.25E-18	1.85E-16	1703491.611	510089.9978	27716.72349	80124.882
152.55212	2.837233	6.20E-18	1.85E-16	7584301.698	24522.58582	10872.25498	7003620.814
168.56528	3.108525	6.18E-18	1.85E-16	16404093.9	552911.0462	172687.9713	14236843.79
698.33097	2.61145	6.16E-18	1.85E-16	448345.3235	2822.694584	1726.930264	221093.5486
196.09688	2.731283	5.89E-18	1.80E-16	8408486.731	856785.0237	724440.1778	8349683.272
200.58637	3.125067	5.72E-18	1.80E-16	5004111.895	587056.2787	215580.6048	4837305.056
283.10309	3.073417	5.77E-18	1.80E-16	7575006.059	96706.57653	181221.8469	7981869.46
320.06927	2.838083	5.91E-18	1.80E-16	25977522.47	316866.3435	235061.0912	19753081.76
375.08831	3.073508	5.80E-18	1.80E-16	3027671.038	50829.82482	158037.1582	2946764.572

Kruskal-Wallis significant metabolites positive ionization mode

619.28982	3.52355	5.92E-18	1.80E-16	1073679.748	7889.545769	4532.912045	487230.7952
159.55997	3.108358	5.34E-18	1.70E-16	19008029.3	706675.258	237278.499	18162272.44
275.08205	3.544983	5.23E-18	1.68E-16	6960710.356	2854735.503	18815.61182	502494.0788
663.29696	2.611583	5.24E-18	1.68E-16	3303215.83	2411.933592	2349.613752	2034324.233
496.17334	3.073425	5.06E-18	1.66E-16	4057853.406	8847.283052	3445.5	4308589.161
664.30051	2.613033	5.08E-18	1.66E-16	1209210.899	1993.022028	1376.723768	705842.2208
452.11298	2.986067	4.99E-18	1.65E-16	4458383.935	1243989.536	27932.98445	54396.83692
324.04035	2.837867	4.94E-18	1.65E-16	950075.4383	3748.109812	2790.666098	817819.5545
619.11239	2.977933	4.79E-18	1.61E-16	4179328.653	383828.1236	3525.831729	5230.108277
184.09694	2.8375	4.68E-18	1.61E-16	382533402.5	1442980.162	827513.7145	305463523.9
242.07636	3.073408	4.71E-18	1.61E-16	21444816.29	2461170.645	284124.0261	21807973.4
279.04265	2.837833	4.69E-18	1.61E-16	4642340.562	8483.98559	8415.948989	3465528.421
296.04543	2.837433	4.74E-18	1.61E-16	978199.6667	33699.33348	4160.698174	742009.6513
436.13544	2.985917	4.72E-18	1.61E-16	600695.5287	196683.1462	9028.923119	7366.27468
578.13787	2.6134	4.71E-18	1.61E-16	521252.492	7933.63025	2822.195751	272466.2013
180.07326	3.108633	4.05E-18	1.44E-16	55587105.5	2125981.982	963921.9087	56389421.93
189.55995	2.721717	4.00E-18	1.44E-16	2783441.027	961413.415	61442.05621	127418.6957
667.21628	3.064767	3.99E-18	1.44E-16	1716405.381	2155.888512	1754.221438	1057606.091
1035.4529	3.518417	3.94E-18	1.44E-16	6984823.274	1753.116632	1387.026317	2357601.304
212.12822	2.646208	3.55E-18	1.31E-16	148856827.1	1338518.175	998776.4711	92675110.61
1034.4496	3.518567	3.54E-18	1.31E-16	12835976.08	2931.207565	1381.240025	4606396.381
262.05003	3.108708	3.46E-18	1.30E-16	10795170.66	763366.5379	230651.2758	9809369.442
198.5471	2.506417	3.39E-18	1.29E-16	5886529.739	3906654.273	105891.511	234393.7954
279.0864	2.7229	3.41E-18	1.29E-16	9167413.95	3676797.674	436638.8702	676534.643
277.08587	3.073442	3.35E-18	1.29E-16	20148824.41	1847400.374	1417876.468	16443285.11
400.08786	2.985767	3.15E-18	1.23E-16	747658.1783	251633.881	7679.501748	11190.75215
330.00588	2.50485	3.10E-18	1.22E-16	674879.3009	281518.7777	2999.570149	9566.232849
446.13407	3.073317	3.09E-18	1.22E-16	3425716.087	76512.46959	7525.398724	2118802.183
291.0863	2.985942	3.03E-18	1.21E-16	161101223.8	34117454.24	538925.0976	1525668.622
393.0639	2.892167	2.94E-18	1.19E-16	2127787.909	362266.3783	23238.38746	58876.80677
258.05047	3.0733	2.90E-18	1.19E-16	1363373.067	198870.0159	16690.56531	1406270.588
323.09151	3.073467	2.91E-18	1.19E-16	5156488.161	328979.8125	121074.5455	4184436.369
294.06169	3.073567	2.84E-18	1.19E-16	3316578.124	100614.4059	35347.56052	2431091.643
429.097	2.986217	2.78E-18	1.17E-16	1197231.177	370900.1467	7153.503081	17919.1484

Kruskal-Wallis significant metabolites positive ionization mode

304.13265	3.082117	2.75E-18	1.17E-16	3690174.766	176665.943	81018.34948	2509554.026
387.03565	2.986025	2.52E-18	1.08E-16	996355.1232	233453.6969	4142.097771	7002.153608
425.0431	3.279225	2.47E-18	1.08E-16	941305.3857	265205.1011	1576.42924	15784.6529
429.05879	2.985967	2.51E-18	1.08E-16	1922811.861	496757.0358	7153.503081	17919.22037
442.06401	2.758525	2.36E-18	1.05E-16	514783.2353	178290.7161	6756.950135	14991.70791
138.09138	3.073342	2.32E-18	1.04E-16	1889583.218	185828.6338	166287.0685	1455029.23
606.1268	2.977625	2.25E-18	1.02E-16	417657.2233	65513.60137	1490.647816	1652.377758
240.14251	3.082117	2.20E-18	1.01E-16	6853293.061	133556.4814	86999.33201	5620282.446
214.53475	2.994917	2.14E-18	9.90E-17	847295.9802	296740.9978	4177.347945	19091.00469
141.04165	3.073483	2.01E-18	9.47E-17	3344662.612	250536.5515	277535.1049	2408259.328
230.03934	2.758333	2.02E-18	9.47E-17	2262789.563	469018.3524	9398.68028	68392.0049
200.11913	3.082217	1.85E-18	8.91E-17	27512352.41	458586.2579	442032.4626	20652511.16
426.05122	2.986417	1.86E-18	8.91E-17	1119406.209	272902.6518	4486.910717	27453.44185
295.0967	3.073633	1.81E-18	8.86E-17	7113147.215	232737.228	218847.2239	4904286.532
428.06228	2.986083	1.73E-18	8.64E-17	5132554.101	1005593.265	13166.25146	60097.25221
646.23781	3.637033	1.74E-18	8.64E-17	1058288.727	1500.116877	1550.538216	457210.9292
449.1325	3.073308	1.67E-18	8.47E-17	11699510.82	218904.5252	13492.57487	7570853.121
239.13909	3.081917	1.63E-18	8.40E-17	59109162.48	779513.625	1093428.569	48705234.37
302.135	3.0735	1.59E-18	8.40E-17	31788301.37	716564.4142	341512.961	22741278.87
318.11258	3.0734	1.61E-18	8.40E-17	285427468.1	3621382.01	1475732.891	226452647
645.23454	3.073308	1.46E-18	7.81E-17	13240243.97	4092.119083	5296.858515	8236667.815
334.08484	3.073467	1.41E-18	7.61E-17	200858206	2901021.686	1257031.238	152117509.4
420.17902	2.617092	1.39E-18	7.61E-17	89666543.75	454623.0083	154129.5507	50540529.98
415.05873	2.721617	1.37E-18	7.60E-17	2255658.59	654879.2284	18819.80389	78749.80856
425.06342	2.98605	1.33E-18	7.50E-17	1509921.496	499312.4542	11822.33703	21276.45661
180.10193	3.07345	1.29E-18	7.39E-17	18258648.42	1401082.113	1049092.041	13155274.15
154.09433	3.073325	1.27E-18	7.36E-17	1977549.729	121909.1549	108523.9533	1371107.828
679.28946	3.5358	1.27E-18	7.36E-17	1587421.338	3958.953458	2426.99329	395831.0302
646.2381	3.073317	1.17E-18	6.94E-17	3956602.99	2142.623212	2558.911112	2616519.115
371.03254	2.506192	1.13E-18	6.81E-17	576996.7856	393699.1205	2504.329443	9997.947435
142.04992	3.073433	1.05E-18	6.45E-17	20478951.71	2045369.263	2854474.58	15083359.77
375.03879	2.986117	1.00E-18	6.33E-17	2181573.7	431652.9355	2457.559986	19650.95711
419.17576	2.61725	1.02E-18	6.33E-17	408534090.2	2318143.104	702470.999	234376026.9
293.05814	3.073483	9.72E-19	6.23E-17	33663201.6	1005825.116	277716.411	24620616.62

Kruskal-Wallis significant metabolites positive ionization mode

322.10551	3.073575	9.56E-19	6.23E-17	4784885.447	235996.7571	32395.09624	3730198.585
310.56148	2.977625	9.27E-19	6.13E-17	10183492.68	1263182.718	3288.374405	9918.305021
448.12929	3.073467	9.04E-19	6.08E-17	64447477.9	1894233.102	43494.35488	40754228.41
200.54851	2.720292	8.82E-19	6.03E-17	1562465.348	396690.7836	6133.616485	60864.56832
153.09103	3.07345	7.40E-19	5.27E-17	31090622.43	1331361.088	1002404.151	22800246.93
197.54326	2.9861	7.59E-19	5.27E-17	789084.7984	247478.8028	2330.156099	17209.16834
205.02783	2.98695	7.34E-19	5.27E-17	1037869.534	806273.5958	57313.78123	168242.0468
620.11578	2.977592	7.51E-19	5.27E-17	1319422.244	182208.7841	2012.919243	2274.905371
645.23444	3.63015	7.00E-19	5.21E-17	3943591.077	2220.15884	1885.179284	1848985.944
206.53328	2.506033	6.34E-19	4.88E-17	913927.4512	473887.5282	6195.257028	27719.36472
605.1234	2.978067	6.44E-19	4.88E-17	1469326.546	193931.3873	1986.938724	2370.451431
198.11256	3.07345	6.05E-19	4.80E-17	8375122378	46811183.4	15731570.47	6258992764
441.06063	2.760492	6.10E-19	4.80E-17	3099674.36	660765.7595	16843.1205	76066.93726
425.04314	2.9954	5.69E-19	4.65E-17	1255915.107	434363.6853	2078.784161	12945.34698
212.02869	2.757833	5.28E-19	4.40E-17	1750180.853	918740.745	235087.2827	281253.4557
226.56016	2.986083	5.05E-19	4.40E-17	20081503.1	6449555.882	73059.74803	520230.4006
330.57314	2.9782	4.80E-19	4.40E-17	3705826.101	583112.1746	2278.253397	6441.020973
369.04621	2.986033	5.03E-19	4.40E-17	665061.867	57772.01022	1674.244372	2396.772099
421.18202	3.523742	5.19E-19	4.40E-17	16861568.31	400548.1809	96665.72956	8539807.612
453.1164	2.986208	5.13E-19	4.40E-17	966728.8695	284092.2661	2971.320359	12526.57733
206.54858	2.9861	4.57E-19	4.33E-17	6857716.516	2193160.626	11206.34588	114086.2881
225.55235	3.278867	4.00E-19	3.88E-17	1881395.454	993443.1239	8192.136569	77770.45876
292.08962	2.985967	3.88E-19	3.85E-17	25610992.02	4395135.625	27783.53732	277364.5657
457.03291	2.758583	3.34E-19	3.39E-17	2313670.145	448791.6004	2815.50644	32559.83219
217.55494	2.98615	3.23E-19	3.38E-17	12282144.27	4219561.299	35636.97782	422738.1412
234.54639	2.994225	3.12E-19	3.38E-17	724431.2052	252781.8121	2886.19085	21349.82514
386.03217	2.986083	3.03E-19	3.38E-17	6313042.283	1346932.909	3555.342773	51605.08829
427.05888	2.98605	3.24E-19	3.38E-17	26814686.79	6902131.509	32603.53186	311815.0409
963.11052	2.977667	2.98E-19	3.38E-17	508734.4161	70457.07817	1381.908447	1510.363544
618.28741	3.524542	2.79E-19	3.33E-17	8603285.944	2432.031963	3364.77982	3849874.924
634.07691	2.977592	2.67E-19	3.28E-17	852850.374	118210.9775	1597.5365	1703.352589
241.54724	2.76685	2.48E-19	3.13E-17	4750518.733	999757.6619	4489.648294	80169.23052
199.04386	2.522283	2.38E-19	3.10E-17	1121462.476	721062.6612	48992.24436	107371.5422
330.04226	2.717708	2.29E-19	3.10E-17	527344.9385	154126.1629	1723.86564	14215.26216

Kruskal-Wallis significant metabolites positive ionization mode

448.1292	3.535883	2.31E-19	3.10E-17	40624561.4	508524.6022	4011.899931	18928826.01
206.04696	2.986083	2.12E-19	3.04E-17	37342107.39	12749834.14	67694.88472	901213.7268
261.07582	2.723017	1.98E-19	3.02E-17	34851764.19	11282349.92	421552.136	1426662.106
374.03218	2.721017	1.98E-19	3.02E-17	673292.859	183746.8496	1746.39222	11697.65406
407.04323	3.004383	2.00E-19	3.02E-17	1311634.664	339267.9658	18842.9394	181262.1156
447.08585	3.12435	2.03E-19	3.02E-17	37820579.56	13119360.06	101689.3713	1707116.027
419.17573	3.526967	1.63E-19	2.83E-17	723420392	2036996.576	294670.0697	339924648.8
211.55488	2.7218	1.54E-19	2.79E-17	2821701.327	1167233.694	82716.09365	239559.7363
420.17907	3.525158	1.22E-19	2.31E-17	155611621.2	749903.195	76483.4873	69694450.92
363.03864	2.716683	9.97E-20	1.98E-17	482429.788	206302.2715	3862.937303	26590.35707
232.54203	2.75795	7.68E-20	1.60E-17	2064195.094	605469.7495	4530.921699	48992.1642
509.07721	3.116283	6.48E-20	1.42E-17	1284355.153	3064.265215	2065.88296	74094.70893
488.03245	2.986217	5.38E-20	1.41E-17	2081726.965	268783.3239	1492.376583	25533.372
504.00472	2.986217	5.65E-20	1.41E-17	1668095.1	231659.5268	1723.838471	19403.39134
616.28099	3.522983	6.05E-20	1.41E-17	143298690.8	5361.002318	3901.739692	61425808.88
617.28441	3.519833	6.08E-20	1.41E-17	46331835.29	9126.192535	2833.572331	19364830.99
947.1383	2.977733	5.22E-20	1.41E-17	633010.4972	92385.89751	1361.54911	1347.956341
569.10243	3.535817	4.38E-20	1.40E-17	3505236.969	121951.4741	1727.500241	34667.35836
413.04318	2.547942	3.65E-20	1.34E-17	898476.1207	493873.26	3771.81318	31030.1518
512.14978	2.986217	3.87E-20	1.34E-17	1959414.481	9235.588741	25152.78109	23156.8876
942.26869	3.543317	3.67E-20	1.34E-17	1204951.904	1529.409586	1380.690778	104643.1314
391.04822	2.98585	2.40E-20	1.11E-17	3266232.559	452900.3799	1449.835753	67296.74703
713.22618	3.539117	2.30E-20	1.11E-17	619361.011	1907.848767	1613.520873	25971.80614
654.23693	3.535883	1.73E-20	1.03E-17	1196427.935	2536.205357	1375.450108	319347.4875
495.14255	3.544483	5.11E-21	3.79E-18	2675326.62	3199.838649	2265.353118	137203.037
640.26924	3.534492	5.45E-21	3.79E-18	2160541.413	2764.373852	2223.513115	598130.7488
639.26643	3.533583	3.33E-21	3.47E-18	12477490.26	7509.254389	4602.714343	3680259.967
638.2629	3.533583	1.62E-21	2.26E-18	38993614.04	12642.80637	1923.756654	12013958.43
523.09266	3.541842	1.03E-21	2.16E-18	2080822.889	1285.906517	1617.131549	123638.4123
494.13912	3.544467	2.30E-22	9.60E-19	10761572.6	2474.84854	1390.223887	564084.9287

Supplementary B- Kruskal-Wallis significant metabolites negative ionization mode

$[M-H]^-$	RT (mins)	P value	fdr corrected P value	Group 1 avg. peak area	Group 2 avg. peak area	Group 3 avg. peak area	Group 4 avg. peak area
198.1489	3.3794	0.000477	0.000988664	352238.4109	269647.5357	555216.5116	499899.3866
448.9473	2.57995	0.000458	0.000950756	993252.5728	1095840.313	1817809.301	1624156.994
257.1029	2.337792	0.000446	0.000927492	748338.3654	952727.5841	423184.2043	583381.5391
472.0785	2.659275	0.000382	0.000794839	2348071.572	1999144.365	773281.7941	1461680.716
441.2854	4.267433	0.000381	0.000793569	234198.1874	123394.2805	379474.2792	92581.7741
250.9694	2.658367	0.000379	0.000790146	864071.1692	891668.1601	516884.8787	437207.3773
306.9471	2.61455	0.000377	0.000785652	581596.5729	1107135.138	294369.1594	315724.8128
238.108	2.841917	0.000368	0.000768757	1403961.327	954507.9992	5231185.451	929114.8728
132.8665	2.530775	0.000366	0.00076435	31524602.09	22061259.46	27855046.23	19208404.81
361.0352	3.1647	0.000357	0.000747564	1130472.258	578603.2751	151562.3439	310954.0023
422.2297	4.91315	0.000355	0.000742964	1280309.22	2283172.811	541878.3683	1037796.202
298.1293	2.537167	0.000344	0.00071985	1395374.266	853400.2618	3074125.969	1148190.468
543.3729	4.197175	0.000343	0.000719256	1131505.053	1229006.479	1557152.962	517221.8624
157.0494	2.428008	0.000338	0.00070874	709880.0625	679332.2948	1008408.947	487096.3581
300.9488	0.475478	0.000318	0.000667373	493438.1768	509337.6055	259624.2665	367461.6384
131.0336	2.0306	0.000305	0.000642074	793471.691	750475.177	1016472.651	697658.0694
563.1189	3.255933	0.000303	0.000637549	1655643.163	609802.3801	93587.18851	695597.1317
295.0909	2.231033	0.000299	0.000629877	708524.0318	709882.9963	1074220.99	1039298.242
222.0766	2.218117	0.000293	0.000619037	929704.9959	974824.9236	1671916.031	1562506.33
384.1607	4.956367	0.00029	0.000612645	482663.1553	477257.1647	220186.58	386593.4142
302.0557	2.473817	0.000289	0.000609846	1005815.009	796722.2818	1142632.447	1776024.124
427.0102	0.745728	0.000285	0.000602106	68328.18893	36162.65086	345793.0293	278884.2284
542.3695	4.197233	0.000284	0.000600359	3926885.01	4263102.149	5261604.225	1967518.735
149.0265	2.183408	0.000269	0.000570939	662220.1879	667605.595	1446323.449	750536.9316
107.0488	2.5216	0.000269	0.000569986	2199440.758	2415230.486	853434.4133	2615557.526
623.015	2.666317	0.000257	0.00054501	273017.9395	361608.259	27614.97029	158147.0625
235.022	2.015767	0.000254	0.000540751	222410.5502	238201.6762	106591.7029	69381.18089
540.3303	4.163467	0.000249	0.000530287	597924.6765	501199.381	1235314.445	361613.4409
324.1563	2.30305	0.000245	0.000520885	428581.3913	220387.1539	743258.9103	518689.815
270.9772	0.889668	0.000234	0.000499282	342882.1175	376297.3779	1006163.215	664022.7234
281.1141	2.454908	0.000234	0.000499282	478763.9625	442101.5911	852586.8847	500307.7841
330.0506	2.35785	0.000234	0.00049922	629700.114	551626.566	893575.2208	481225.3056
216.0871	2.581083	0.000216	0.000461466	444889.338	330071.6124	771099.2747	608253.3801

Supplementary B- Kruskal-Wallis significant metabolites negative ionization mode

447.134	3.868883	0.000215	0.00045928	546492.8921	837131.1081	151450.8588	243883.8013
319.1332	2.380033	0.000211	0.000452125	974190.6035	575503.0092	772868.4844	557186.2473
328.0349	2.453133	0.000205	0.000440359	415370.5998	406438.6754	618019.0516	428076.696
432.8436	2.90695	0.000205	0.000439778	375337.5912	732828.2795	84539.2836	169567.6466
491.3011	3.915467	0.00019	0.000407989	250281.9379	676427.0061	841399.61	316283.2968
301.1403	2.503917	0.000184	0.000395943	878204.5087	847860.0986	1417478.691	976323.0608
490.3099	4.153817	0.000183	0.00039437	456979.6727	281801.3238	547985.8291	315444.268
246.098	2.356	0.000182	0.000393001	4648864.785	4132546.492	7629033.763	2937280.341
459.2961	4.284675	0.00017	0.000366326	492524.9928	266709.2747	87964.7083	143053.8323
230.0818	2.721367	0.000169	0.000364581	468637.7643	399008.402	726761.178	738743.4805
172.0604	2.069125	0.000165	0.000355934	447380.2725	506051.9238	804249.9769	518456.3835
290.9403	0.472783	0.000164	0.000355375	699321.8774	571516.6682	446134.1547	508951.12
408.1606	4.712125	0.000164	0.000355375	1128349.093	821349.061	519969.2793	956391.3264
254.1031	2.4058	0.000164	0.000355151	382735.7229	261711.27	704549.8737	289433.7198
387.2176	4.417383	0.000159	0.000345785	3756828.655	4496034.266	698202.9482	5613951.432
332.1008	3.6261	0.000156	0.000339059	1533501.64	395359.6361	535557.0129	1508606.953
255.012	0.886475	0.000153	0.000331624	253288.0172	352627.1041	1308513.605	781698.6362
595.2145	2.76315	0.000151	0.000327561	192960.5477	463961.0228	11186.77114	70549.49403
232.9975	1.163143	0.000142	0.000309515	2898940.499	1845731.218	3611131.736	2052430.257
241.8821	0.499841	0.000142	0.000308572	737367.8856	770756.0307	427263.0187	530058.8357
227.9598	0.795318	0.000134	0.000293391	1035411.534	1350246.742	1222935.205	2366344.691
346.286	5.008583	0.000133	0.000289905	357250.1604	371986.799	114144.4117	408953.7262
399.1781	3.067917	0.000133	0.000289905	351293.7481	1559873.526	125257.3374	185229.147
206.9573	0.498569	0.000129	0.000281769	482015.3541	410197.7469	272828.3292	331745.3287
295.9622	2.520992	0.000126	0.00027698	288440.9228	358405.5645	152873.8964	354362.5418
244.0823	2.450417	0.000125	0.000274968	3668718.373	2835296.598	6295156.49	3267462.809
421.2263	4.921483	0.000124	0.000271421	7435087.231	13021173.79	3347358.549	6208215.301
190.0864	2.869275	0.000123	0.00027035	422681.0729	456015.0807	768949.4675	1092768.695
295.082	2.48385	0.000123	0.00027035	703982.8706	572229.2963	429116.2715	297753.8275
161.0808	2.181683	0.00012	0.000264725	368015.8688	254941.5217	907684.7527	416497.702
339.1811	2.857742	0.000115	0.000252602	478753.3961	511944.9089	210511.9105	687114.2157
291.0986	2.188	0.000111	0.000244342	969777.4085	1315860.577	1657676.393	2894905.087
653.2615	3.186033	0.000105	0.000231142	263578.6491	546642.3465	646482.5439	235913.6294
226.0691	2.042717	0.000104	0.00023106	482098.4734	456890.3482	415606.1645	891271.2721

Supplementary B- Kruskal-Wallis significant metabolites negative ionization mode

286.1083	3.218167	0.0001	0.000221809	69517.54714	27826.251	588211.7618	45646.68692
111.0073	0.744025	1.00E-04	0.000221572	1089453.396	534517.2294	3269543.552	3357656.754
193.0229	0.745436	9.66E-05	0.000214339	341104.5255	224453.4745	977203.4026	674938.4486
395.2071	3.136817	9.60E-05	0.000213236	4246623.985	4530375.788	813232.579	3729401.872
331.0521	3.416983	9.53E-05	0.000211859	2654676.902	4134558.606	304870.4592	2290449.109
483.2109	4.385492	9.02E-05	0.000200777	170653.6558	62300.35802	14266.28036	189385.4678
184.1332	3.2138	9.00E-05	0.00020044	277836.6205	376631.1731	566040.129	680360.4975
250.0718	2.465775	8.96E-05	0.000199764	882589.7446	725959.9993	1799102.153	1091048.89
164.9261	0.449858	8.92E-05	0.0001993	3260369.383	2385897.446	2014710.505	2433000.789
236.056	2.21715	8.93E-05	0.0001993	346637.3796	230124.55	1049855.269	259424.7183
242.1223	2.385933	7.95E-05	0.000177734	907731.3619	1303630.466	2066512.468	2442321.27
303.2175	2.99305	7.85E-05	0.00017567	2695408.374	4119455.107	6127114.17	3546300.236
303.0506	2.976867	7.55E-05	0.000169179	1816328.946	1439421.382	292498.8193	859235.6439
173.0081	0.745237	7.51E-05	0.000168365	516301.597	383042.3392	1175740.706	1225399.044
232.7634	2.522708	7.38E-05	0.000165636	4744270.11	1088545.071	1312996.487	1348764.974
159.0844	2.037983	7.36E-05	0.000165441	498654.2403	524154.1367	679212.5616	1138874.637
184.0969	2.563233	7.21E-05	0.0001621	1131682.985	887086.5116	1751618.986	1752253.525
377.0988	2.545467	7.20E-05	0.000162097	137092.6003	112702.0575	517005.4577	176563.1228
496.2678	4.171867	7.15E-05	0.000161139	650815.1397	406999.8253	119196.2891	600483.6955
319.099	3.235133	6.92E-05	0.000156183	129697.4927	151944.4169	124961.5819	531744.9066
416.2133	3.899217	6.45E-05	0.000145642	280544.1663	360645.6148	14906.66121	186650.9354
280.9827	5.3316	6.38E-05	0.000144315	4256828.096	4296129.112	3719540.011	3673219.52
380.0854	3.489583	6.18E-05	0.000140006	139852.8152	112753.5169	8598.537854	117662.2204
174.955	2.851933	6.17E-05	0.000139813	65601517.09	101710748.8	96011257.18	87173422.82
266.1033	2.7467	6.15E-05	0.000139552	589501.0283	233562.4764	1063368.603	242018.1645
442.1617	2.920908	6.12E-05	0.000138896	103080.069	35343.00241	358547.2901	56448.0075
534.2081	4.384217	6.01E-05	0.000136691	180846.6805	70076.98633	7971.643579	223716.6341
320.0774	2.650767	6.00E-05	0.000136616	109013.6092	47141.15284	905582.1082	170441.0845
295.1297	2.615717	5.93E-05	0.000135018	680351.83	619116.9698	1005597.162	391410.6038
241.119	2.37815	5.73E-05	0.000130687	10769601.22	15527334.99	21632734.7	28622525.14
261.9416	0.739745	5.42E-05	0.000123722	1598550.971	1265323.318	2885996.497	3463981.664
304.2208	2.99525	5.08E-05	0.00011618	217302.0103	284647.3231	733244.3066	435451.765
290.0881	2.151558	4.89E-05	0.000111819	169290.8412	105157.5496	761521.5187	241011.9921
543.1557	4.386617	4.66E-05	0.000106707	71059.4882	33783.8642	3342.16223	100003.5173

Supplementary B- Kruskal-Wallis significant metabolites negative ionization mode

308.0774	2.384267	4.60E-05	0.000105401	321317.9064	329263.7582	874884.8296	540941.8071
242.1394	3.379058	4.12E-05	9.46E-05	732724.2083	725445.9384	1661403.818	1451482.03
555.0275	2.659433	4.08E-05	9.36E-05	862617.3193	910589.989	78827.36975	500373.4136
345.2069	3.754717	4.02E-05	9.24E-05	1545557.757	2446025.452	223825.5312	1115575.772
373.1251	2.436667	3.95E-05	9.09E-05	567144.2859	547285.8209	1070464.027	597799.4913
405.228	3.559533	3.80E-05	8.75E-05	256776.4297	451755.2188	28828.97734	306056.5972
461.0488	3.419783	3.75E-05	8.64E-05	308458.4524	326507.2671	18857.28456	300630.8154
546.1639	4.385658	3.69E-05	8.52E-05	66651.64275	36539.12962	3471.144381	96165.67105
456.1774	3.040533	3.63E-05	8.38E-05	69094.79972	46083.09028	748122.4792	112576.2269
448.9473	3.484433	3.60E-05	8.32E-05	2726156.398	2075907.954	3532904.082	4634870.769
487.2687	3.378	3.56E-05	8.25E-05	982111.9673	2042294.429	156532.8642	562918.7719
489.3066	4.154733	3.55E-05	8.24E-05	1825458.252	1195865.176	2707709.411	1227651.821
371.1821	2.863342	3.50E-05	8.12E-05	222921.8998	229893.926	491518.8433	324430.9143
409.2229	3.064783	3.43E-05	7.97E-05	676586.9685	2207084.942	99566.66439	682892.3374
420.9936	0.748633	3.37E-05	7.84E-05	142886.2669	35326.81049	849210.8282	699642.2294
288.04	2.038517	3.34E-05	7.78E-05	436881.5772	563446.4591	467370.3883	1284986.385
188.0343	2.597117	3.24E-05	7.56E-05	809318.7664	391059.9919	1289573.942	779235.8837
457.9402	0.745143	3.11E-05	7.25E-05	90854.63831	39100.84932	463373.5841	453934.7409
330.2365	3.065	3.06E-05	7.15E-05	119921.877	540863.6742	687554.3656	1445189.458
225.1127	2.465133	3.03E-05	7.08E-05	1282252.139	1156386.846	2554882.265	1214280.269
442.9754	0.745143	3.03E-05	7.08E-05	42780.8691	12249.9329	407345.1591	268235.914
230.7658	2.519	2.90E-05	6.80E-05	3359046.175	881491.4298	956661.9981	920172.5165
310.9691	0.734218	2.87E-05	6.72E-05	294082.7212	110694.7868	743402.4384	598017.8378
388.221	4.415833	2.85E-05	6.69E-05	1076183.477	1000449.205	136069.7085	1675585.774
224.0923	2.57175	2.83E-05	6.66E-05	2000742.005	332033.1037	2497770.427	3092256.69
342.9594	0.73918	2.83E-05	6.66E-05	382528.6439	176701.7848	882202.6949	890216.1005
158.0811	2.042667	2.72E-05	6.41E-05	7113660.178	8797363.802	5777511.898	18998909.11
371.0771	2.392067	2.71E-05	6.40E-05	422214.5204	581797.5057	873433.7355	882602.765
527.2857	2.825083	2.67E-05	6.31E-05	86649.32989	128927.3836	2710.895221	34608.92063
275.0368	3.33545	2.61E-05	6.16E-05	77921.29893	64836.76077	680828.9042	137042.0252
342.044	0.754847	2.55E-05	6.04E-05	54747.34365	33093.65401	229366.0003	400028.5929
175.0601	2.281783	2.51E-05	5.95E-05	2487611.481	1554660.625	3073263.556	1127660.258
265.1419	3.315275	2.48E-05	5.89E-05	1255291.702	621081.0807	739490.2884	1290751.119
363.245	3.812317	2.39E-05	5.68E-05	669501.7866	1192261.071	134153.5333	405864.8454

Supplementary B- Kruskal-Wallis significant metabolites negative ionization mode

257.0802	2.598133	2.29E-05	5.45E-05	2201915.889	1412102.64	1193976.721	2045567.339
450.2624	3.783525	2.26E-05	5.36E-05	514953.9367	672866.3623	177864.0026	520306.628
339.1752	2.4369	2.12E-05	5.06E-05	376506.1029	311098.2532	787733.2217	411003.3466
820.4598	4.379217	1.93E-05	4.60E-05	107137.4256	33066.14266	1339.336062	97095.45263
252.1238	3.2188	1.89E-05	4.50E-05	171288.522	81277.78015	1296436.9	101599.4601
358.0092	0.764543	1.81E-05	4.32E-05	47946.14202	25220.34688	217410.8593	373454.6046
200.1283	2.80815	1.74E-05	4.17E-05	2130184.519	1876580.093	4007041.51	5047742.499
632.3178	3.424367	1.58E-05	3.79E-05	229773.81	312664.7521	615663.5428	145429.0924
371.1094	2.220817	1.46E-05	3.51E-05	232856.7229	151208.5026	571587.722	234565.0688
474.2785	3.707417	1.46E-05	3.50E-05	479185.153	489825.8403	1119143.438	432449.5618
527.2634	4.3871	1.45E-05	3.48E-05	106470.2251	47584.28856	3036.88289	144210.9667
358.1659	3.135467	1.43E-05	3.44E-05	106875.0601	51296.72364	1452571.472	168239.7778
261.0879	2.321267	1.35E-05	3.25E-05	3353359.605	2522879.506	6521394.696	3579464.832
202.0712	2.342617	1.32E-05	3.19E-05	1004328.492	806047.9737	1388214.66	1008151.548
374.8969	0.75367	1.30E-05	3.15E-05	829553.3311	807081.7023	1418967.809	1885684.367
170.96	0.746098	1.28E-05	3.09E-05	535803.6476	342797.2526	1122491.66	1024990.299
174.955	3.391067	1.26E-05	3.05E-05	45558338.15	57699804.48	74493493.4	45826471.81
338.1721	2.440283	1.25E-05	3.02E-05	2748218.306	1907258.147	5596538.689	3178411.298
387.0453	3.10535	1.25E-05	3.02E-05	1659431.209	675125.5187	252668.5421	2673047.213
375.0719	2.965233	1.24E-05	3.02E-05	789069.1304	256404.4871	235144.2636	47282.54831
134.8636	2.986067	1.21E-05	2.94E-05	4050806.356	6470250.53	8519429.622	4135167.366
289.9366	0.745013	1.19E-05	2.90E-05	2743069.425	1341307.079	6245525.251	6366105.631
240.0849	2.483317	1.19E-05	2.89E-05	713656.8617	353572.016	1148354.6	1890105.279
262.0913	2.3235	1.17E-05	2.86E-05	361890.9146	264500.7088	685254.2447	369249.388
200.0919	2.44805	1.15E-05	2.81E-05	1758581.578	2300373.937	3603092.934	2797661.318
423.1335	3.468208	1.14E-05	2.79E-05	339921.5914	551143.099	447682.3947	272919.9431
123.0438	2.017742	1.11E-05	2.71E-05	1991850.201	1793630.19	834355.5144	669805.1651
164.9463	0.448602	1.11E-05	2.71E-05	2670143.827	2381263.946	1619445.27	1741719.502
286.0931	2.5793	1.06E-05	2.61E-05	749281.577	178123.2649	1160558.005	659662.5656
111.0073	1.953608	1.06E-05	2.60E-05	298652.3645	198616.1238	988654.8484	509123.6224
467.2239	4.384167	1.06E-05	2.59E-05	407673.2603	173496.1487	31651.62271	594589.6926
228.1236	2.668033	1.05E-05	2.57E-05	2106605.779	471120.5244	997267.8266	3596845.231
214.0714	2.373583	1.03E-05	2.54E-05	548018.805	637664.1703	1062108.082	661484.4233
342.0505	2.664683	1.01E-05	2.48E-05	1339826.422	792900.0597	1169207.794	1894378.931

Supplementary B- Kruskal-Wallis significant metabolites negative ionization mode

230.1029	2.464767	9.56E-06	2.36E-05	975059.5403	574668.2443	1286501.847	1240720.57
312.9266	0.736587	9.45E-06	2.33E-05	1043370.585	860863.5324	1927455.87	2102203.287
214.1441	2.973833	9.43E-06	2.33E-05	272442.5983	209632.5332	599760.6275	873505.4884
303.1254	2.951092	9.22E-06	2.28E-05	2744906.126	1115592.121	376173.9776	1058145.259
354.2033	2.632092	9.14E-06	2.26E-05	432199.5456	472623.9243	1039358.898	766037.2969
819.4563	4.379517	9.00E-06	2.23E-05	165019.3958	72766.33769	1312.256103	165667.9051
471.0751	2.659383	8.69E-06	2.16E-05	10548563.2	9224913.229	1202849.99	6504689.473
329.8992	0.749371	8.40E-06	2.09E-05	407650.6689	257857.6934	646348.6294	847403.1027
358.9245	0.745112	8.36E-06	2.08E-05	594441.7062	387450.6908	1190858.066	1252037.015
369.0977	2.632075	8.22E-06	2.05E-05	657049.6197	662134.4302	172414.9782	462587.438
539.0626	2.659667	8.20E-06	2.05E-05	2220851.098	2147485.393	182301.6647	1436633.777
144.0442	2.21615	8.05E-06	2.01E-05	536138.5076	416043.3009	1060711.995	606191.0496
373.1866	2.633183	7.80E-06	1.95E-05	160202.747	110931.5448	923508.0777	233371.3777
381.1735	5.005283	7.79E-06	1.95E-05	296237.3337	282619.2684	41157.17251	373626.105
304.1037	2.350917	7.57E-06	1.90E-05	684872.087	285568.7775	2943139.911	765379.7016
562.3146	4.15225	7.54E-06	1.89E-05	348822.866	450049.2558	662888.3014	183105.6718
540.066	2.659333	7.48E-06	1.88E-05	555843.6534	559827.7117	61513.61383	419645.1481
284.1502	2.739467	7.35E-06	1.85E-05	47020.36632	25860.55702	408086.7511	96011.12883
274.9722	0.746457	7.11E-06	1.79E-05	1721879.388	744934.0254	4691756.762	4361531.869
449.2154	2.75655	7.07E-06	1.78E-05	331381.2263	492446.5642	84616.21316	215176.7818
194.0813	2.207483	7.01E-06	1.77E-05	1108287.441	587682.8577	353525.6005	862789.7712
338.0881	2.452917	6.56E-06	1.66E-05	655633.1202	529096.4406	1557067.074	620813.8356
405.0285	0.745143	6.53E-06	1.65E-05	75686.70918	11739.61166	807755.3448	670599.5677
128.034	0.764758	6.40E-06	1.62E-05	8353904.891	11575000	14577542.06	30195724.67
255.1235	2.493858	6.13E-06	1.55E-05	813725.42	1141845.091	344711.4277	677681.8668
536.2942	4.267167	5.96E-06	1.51E-05	724308.1278	923834.3979	77452.68758	229566.3989
435.958	0.748513	5.74E-06	1.46E-05	91353.73179	11191.74108	788580.4797	693071.9408
234.761	2.513525	5.70E-06	1.45E-05	3163421.016	635086.305	752610.8694	925529.6223
248.8997	0.460119	5.56E-06	1.42E-05	3702766.113	4921938.226	1926843.48	3206989.401
116.0703	2.098333	5.51E-06	1.41E-05	806524.6466	592799.2802	2074453.02	1227642.115
332.0724	3.105233	5.48E-06	1.40E-05	5767802.484	1592478.685	2859152.408	10436154.35
191.0189	0.7362	5.43E-06	1.39E-05	26957572.74	9941963.502	86110265.52	80918424.26
379.2488	3.351967	5.42E-06	1.39E-05	3785290.804	6324541.318	657634.6229	2352519.077
184.9537	0.751898	5.19E-06	1.33E-05	230206.4902	136665.36	699938.0223	657284.3201

262.0719	2.631717	5.09E-06	1.30E-05	842010.8629	983913.6308	2177567.016	1316836.411
443.2048	3.334442	5.07E-06	1.30E-05	701735.3868	817141.0146	61892.26245	860114.0127
411.2386	2.727983	4.97E-06	1.28E-05	684020.1238	1149837.136	235907.953	606309.4594
218.1028	2.053917	4.86E-06	1.25E-05	3349084.489	2725477.372	9267281.521	4288926.363
289.0506	3.035383	4.64E-06	1.20E-05	6669564.265	8657081.359	655318.3606	2128447.007
518.3693	3.326408	4.50E-06	1.16E-05	516010.5257	869281.9051	1470083.522	296031.333
396.9575	3.754692	4.29E-06	1.11E-05	753611.9169	561645.3307	156847.418	203770.944
151.0388	2.521675	4.23E-06	1.09E-05	5123748.279	5785415.689	1681732.48	5618514.026
268.0801	2.791033	4.13E-06	1.07E-05	355988.3287	891034.2076	1004469.977	944804.573
236.0559	2.44155	4.06E-06	1.05E-05	559565.0383	514129.3662	958681.4642	567838.3422
296.9539	0.723066	3.97E-06	1.03E-05	775397.0642	473893.5186	1877236.506	1427018.725
343.1145	2.474183	3.86E-06	1.00E-05	1000481.336	895183.7575	2588999.363	1471732.983
345.0114	1.903375	3.83E-06	9.96E-06	1059733.824	723312.3477	721060.0271	752367.8825
403.1719	2.497867	3.68E-06	9.59E-06	82491.32078	44032.8993	643386.2715	141121.1286
309.1067	2.386908	3.65E-06	9.52E-06	711292.3355	1003382.39	1669042.821	1810655.391
192.0222	0.744553	3.63E-06	9.49E-06	1111047.786	354338.6185	4174298.378	4011523.016
323.1861	3.16955	3.49E-06	9.11E-06	492178.5724	674983.6035	137555.0725	620165.2655
535.2908	4.267283	3.37E-06	8.83E-06	2559765.763	3409565.292	356953.3985	929288.6381
230.1029	2.736667	3.37E-06	8.82E-06	452881.0973	392013.9627	780511.6561	707461.7678
117.0179	0.940846	3.33E-06	8.73E-06	6622775.495	7721409.719	31482781.09	14578213.32
330.0506	2.791467	3.20E-06	8.39E-06	159236.5847	483017.8646	514672.889	439581.9991
177.0546	3.000842	3.12E-06	8.20E-06	359718.7596	997860.3834	49839.51297	183729.0631
245.9465	0.737243	3.07E-06	8.09E-06	507136.1229	298483.4062	1351474.201	1108931.787
303.062	2.2252	2.96E-06	7.80E-06	539244.2141	314432.8921	1509617.494	555480.5593
228.9935	2.825467	2.93E-06	7.73E-06	1181686.164	1402424.492	2172261.55	1396705.205
205.0346	1.936667	2.88E-06	7.60E-06	808492.9927	702226.5712	3488278.551	1182083.162
326.0855	2.34965	2.71E-06	7.17E-06	333437.5811	223292.466	609163.4466	328609.0783
525.27	2.772967	2.62E-06	6.94E-06	132269.2685	289954.3298	5347.648236	37048.86551
169.0495	2.177467	2.56E-06	6.79E-06	3920350.536	9551733.881	14592003.71	14367517.67
511.1856	2.755767	2.46E-06	6.53E-06	172806.4359	319945.3923	11974.73898	47271.53959
156.0654	2.044133	2.34E-06	6.21E-06	669712.513	489953.1051	1225821.237	536968.6225
182.0449	2.31805	2.32E-06	6.17E-06	415382.8357	97409.30392	140179.3292	392231.0846
202.1076	2.503975	2.22E-06	5.91E-06	541861.0569	852783.7187	1524859.732	1189476.224
233.9464	0.968332	2.17E-06	5.79E-06	769392.1776	849515.1808	1400365.841	1134442.474

Supplementary B- Kruskal-Wallis significant metabolites negative ionization mode

563.3222	3.141083	2.14E-06	5.72E-06	143739.0527	78048.16724	1960.255456	82276.75492
375.072	2.720233	2.13E-06	5.71E-06	855974.8046	293407.9311	119798.3231	36627.44399
261.0291	2.476133	2.07E-06	5.54E-06	676550.1994	984741.8778	1247484.57	563822.6767
388.0494	3.291525	2.04E-06	5.46E-06	421328.7689	70430.49347	10511.27884	122548.0808
365.0662	3.0796	2.00E-06	5.38E-06	4691039.032	4119719.74	817665.3032	4198344.539
427.1791	3.488325	1.99E-06	5.35E-06	174902.1732	198001.5135	15367.43917	330926.8157
145.0129	0.506118	1.97E-06	5.29E-06	954622.9459	672276.6317	1959617.659	756047.2723
301.0503	3.055958	1.97E-06	5.29E-06	4465746.216	2339224.912	462581.6623	2210294.842
268.9583	0.474803	1.93E-06	5.19E-06	616725.5323	698830.3993	166207.3852	353510.476
218.0453	2.325992	1.77E-06	4.77E-06	2824928.257	1300961.712	1192628.633	1455177.457
193.0497	2.430417	1.73E-06	4.68E-06	2653898.855	1787748.852	650844.6216	1332341.066
228.0872	2.56395	1.73E-06	4.67E-06	2526788.799	2434595.57	6776844.338	3108124.454
332.1139	2.536058	1.66E-06	4.50E-06	576946.1334	321074.9672	1481585.763	573987.3835
301.104	2.308583	1.65E-06	4.48E-06	672502.7207	440998.4504	1300438.952	585317.9592
289.1204	2.9667	1.62E-06	4.41E-06	27327399.73	74506690.5	961992.4722	15109538.19
571.0006	2.6596	1.60E-06	4.35E-06	477461.8781	461696.7504	25708.53043	234255.2756
234.9836	0.741243	1.56E-06	4.26E-06	771676.0875	490681.3657	2357671.819	2196570.224
441.2491	3.34365	1.56E-06	4.25E-06	631079.7072	597017.9485	142496.5879	411366.3249
226.9175	0.501415	1.55E-06	4.23E-06	2388188.639	2720404.49	1355001.933	1897203.531
341.1909	2.491833	1.54E-06	4.22E-06	291606.5912	315505.2076	910288.6964	510764.6591
340.1875	2.494867	1.47E-06	4.01E-06	1995065.013	1940357.126	6360259	3214882.86
295.0134	2.597025	1.35E-06	3.69E-06	418239.5691	533402.0691	549309.4989	650622.7654
274.0437	3.18535	1.30E-06	3.57E-06	329378.7924	48057.17271	17343.00493	10882.46379
206.0815	2.57975	1.29E-06	3.54E-06	2037031.672	3462125.177	5300283.962	5970388.981
132.8665	0.35405	1.23E-06	3.39E-06	10818111.97	14555019.6	13701516.2	9059172.108
391.2489	3.7575	1.20E-06	3.32E-06	460330.3644	1986792.104	40984.85836	304351.4501
131.07	2.478783	1.19E-06	3.27E-06	7544359.821	9955383.729	17404822.34	7289671.371
367.082	2.5812	1.17E-06	3.24E-06	1402022.784	1924207.407	358790.8125	1255814.031
182.9876	2.82545	1.14E-06	3.15E-06	15658892.52	17993523.93	33317293.45	21311841.54
374.1719	2.72895	1.14E-06	3.14E-06	358610.2621	394359.3162	674197.5699	544354.9087
538.151	4.381658	1.10E-06	3.05E-06	443060.9597	134571.8905	8422.999604	519224.8932
274.0568	2.00045	1.08E-06	3.00E-06	901997.7902	811610.421	2059887.191	1053703.274
172.0968	2.471792	1.08E-06	3.00E-06	35864158.49	8003394.776	18513962.09	48341362.93
215.1111	2.55265	1.07E-06	2.98E-06	1429474.574	959791.9929	783226.7403	2342349.982

Supplementary B- Kruskal-Wallis significant metabolites negative ionization mode

359.1459	2.447167	1.03E-06	2.87E-06	446489.1988	361697.775	1152837.314	495958.4927
607.1304	2.614433	1.01E-06	2.81E-06	155543.1612	126430.5541	667676.074	202880.4413
241.1078	2.62455	9.69E-07	2.71E-06	528409.0753	514821.987	972491.4673	599501.0832
312.1452	2.75575	9.57E-07	2.68E-06	1410781.948	133024.379	1213193.684	954556.408
132.0733	2.47725	9.14E-07	2.56E-06	247045.5726	325265.0818	633818.0013	236255.1571
216.9436	0.9505	9.04E-07	2.53E-06	1553428.336	1831274.236	4621323.957	3092322.508
383.2346	2.764433	8.49E-07	2.38E-06	550872.0609	1118963.841	129714.5397	288168.506
290.0717	3.450117	8.12E-07	2.28E-06	1000510.945	401865.819	66603.63225	80066.53864
315.0661	3.082258	7.93E-07	2.23E-06	4423273.655	3105861.193	485773.4287	2501145.654
259.1014	2.659333	7.90E-07	2.23E-06	635371.5624	684342.945	1206124.934	776460.5445
287.0408	2.18945	7.86E-07	2.22E-06	551585.0472	386042.2487	1076042.821	537028.5966
431.2047	3.542467	7.85E-07	2.22E-06	952892.787	3398145.261	319192.016	811510.7417
258.0072	2.037533	7.33E-07	2.07E-06	166260.4225	243689.1317	127473.3622	521915.2679
134.8931	3.064417	6.43E-07	1.82E-06	5048077.346	9122241.838	8358437.85	3849110.371
566.1458	4.384267	6.27E-07	1.78E-06	209385.0401	69685.68398	2013.948987	250513.3481
286.0907	2.0523	5.45E-07	1.55E-06	303043.6457	255867.3555	884118.9339	406912.476
414.2283	3.670667	5.43E-07	1.54E-06	281001.1475	118633.5597	7948.637595	511413.3246
283.1662	2.788267	5.41E-07	1.54E-06	168912.424	130191.7995	581715.9159	191125.8679
363.0874	2.975483	5.37E-07	1.53E-06	6343848.963	3797376.73	506190.0166	1828249.517
357.0151	2.686517	5.30E-07	1.51E-06	629943.1138	363894.1495	373725.4007	647037.5266
378.2364	3.284042	5.21E-07	1.49E-06	1410910.829	2794490.476	82258.27249	706468.6218
297.1453	2.782075	5.03E-07	1.44E-06	822301.4382	887278.987	1710666.951	1123155.526
609.3276	3.140867	4.88E-07	1.40E-06	112608.1275	82403.62021	1364.416955	68841.23519
456.192	4.384267	4.73E-07	1.36E-06	240139.0371	189401.3849	20047.90808	328717.9509
379.0821	3.487567	4.72E-07	1.36E-06	923771.934	933113.7152	40833.62793	756832.479
612.3514	3.7475	4.18E-07	1.20E-06	62967.06156	147627.4064	648877.6756	167485.5596
136.8617	0.35168	4.15E-07	1.20E-06	1888638.853	2567466.316	2827588.799	1662229.523
428.2074	3.283325	3.84E-07	1.11E-06	170063.2052	154022.2054	6297.242954	217037.1347
569.9922	2.659383	3.82E-07	1.10E-06	329846.3661	336721.3188	20074.94015	172730.117
329.0453	3.082542	3.60E-07	1.04E-06	3614281.342	2750704.02	452128.8896	3686910.408
445.2217	3.256475	3.54E-07	1.03E-06	412749.5663	553837.9752	76873.75684	515360.5759
216.9088	0.465757	3.50E-07	1.02E-06	1973973.721	1687060.347	968016.8608	887866.1136
397.2229	2.579617	3.41E-07	9.91E-07	912252.1307	3635405.249	392461.219	697680.7128
147.065	1.170699	3.33E-07	9.69E-07	2219370.339	1223285.694	333161.6515	882415.7723

Supplementary B- Kruskal-Wallis significant metabolites negative ionization mode

405.0614	2.472467	3.03E-07	8.83E-07	123366.5264	236124.0105	359373.4939	529534.5259
350.075	2.659425	3.00E-07	8.74E-07	6180549.849	6390003.013	978008.8142	4235029.673
194.045	2.3372	2.85E-07	8.33E-07	1643644.752	1430260.499	768741.9952	2164798.756
411.2386	3.552667	2.80E-07	8.19E-07	502662.3196	952994.6868	82418.58174	348068.5469
180.0656	2.146958	2.77E-07	8.10E-07	305172.7742	262587.7765	1072540.78	611079.4207
257.0277	0.971113	2.73E-07	8.01E-07	447113.4471	462880.0928	1496725.866	992222.927
185.9253	0.517118	2.70E-07	7.92E-07	290938.6717	132123.6344	1816488.223	299922.5711
216.0871	2.123467	2.61E-07	7.67E-07	384794.5594	399321.8939	987984.5642	707762.8775
493.9939	2.91275	2.39E-07	7.04E-07	374359.2847	359066.8447	25041.2582	280035.1452
387.0639	3.073767	2.31E-07	6.81E-07	2710105.986	1362434.924	502627.4256	1615003.347
285.1149	3.495867	2.25E-07	6.65E-07	1056974.473	68604.6598	153818.0331	1145609.748
199.0579	2.48095	2.16E-07	6.39E-07	296834.6529	458007.2739	852098.2438	346175.7309
389.061	3.073058	2.16E-07	6.38E-07	591942.7313	245005.7221	22062.46596	129174.4356
190.0534	2.25075	2.03E-07	6.00E-07	555105.4393	569735.452	2019884.908	2084633.287
349.0716	2.66685	2.00E-07	5.93E-07	33495729.52	33292514.89	4150569.294	22049014.96
469.0595	2.676517	1.98E-07	5.88E-07	3012466.137	2708438.033	207109.9981	971965.8896
220.146	3.465517	1.97E-07	5.87E-07	477706.8953	505749.1321	795477.2557	358996.9611
134.8932	3.545467	1.83E-07	5.44E-07	8483488.59	5442989.293	3314810.352	6632586.568
355.1509	2.65185	1.82E-07	5.44E-07	702111.4559	651666.4067	1507146.653	829211.0195
280.08	2.6598	1.77E-07	5.27E-07	2156343.422	1371477.018	2602656.495	3657930.942
225.7953	2.377633	1.73E-07	5.18E-07	835747.843	208059.2986	194523.6325	752759.3552
220.0609	2.386983	1.32E-07	3.95E-07	422104.918	405367.2301	914877.3095	459450.1157
206.9719	2.598283	1.26E-07	3.79E-07	3066877.868	5969353.907	1415901.599	2105948.109
329.1353	2.536367	1.25E-07	3.74E-07	505711.9617	433838.9929	1895896.141	667021.9764
305.022	3.644017	1.12E-07	3.38E-07	226216.0591	200994.7172	4660.685557	36273.16597
270.0563	2.502917	1.11E-07	3.34E-07	421780.7203	60225.75297	239900.8531	351433.3794
284.1218	2.540392	1.08E-07	3.25E-07	2187072.893	2067063.693	519525.858	1430921.42
261.9416	0.97794	1.05E-07	3.18E-07	1166741.907	1246387.57	2792901.995	1907801.595
251.1261	3.165267	9.70E-08	2.93E-07	240266.467	541188.3999	554890.8745	259361.3276
296.9812	2.819117	9.57E-08	2.90E-07	721817.7966	995586.1372	1459580.706	714003.6429
397.0925	3.076225	9.03E-08	2.74E-07	2476316.146	1350432.306	137920.7533	1132031.327
377.2331	3.27475	8.70E-08	2.64E-07	8253191.089	14388135.24	694335.0206	3448161.201
244.0823	2.659383	8.63E-08	2.62E-07	1940295.175	2083046.451	5293967.317	2498243.826
379.2487	3.569917	7.62E-08	2.32E-07	2032538.503	3010006.917	420076.2852	1427482.428

Supplementary B- Kruskal-Wallis significant metabolites negative ionization mode

544.364	3.743317	7.10E-08	2.16E-07	481735.4547	872851.4224	3946551.379	937410.1596
185.0054	0.97491	6.91E-08	2.11E-07	448580.7548	571150.4704	1109544.067	771011.0652
382.2314	2.765117	6.83E-08	2.09E-07	7057842.35	14354131.21	755644.0073	2422303.569
375.2175	3.341617	6.47E-08	1.98E-07	15371111.39	13674519.53	860320.865	16126659.99
545.3675	3.746333	5.95E-08	1.82E-07	133981.7226	252273.1369	1262587.599	291694.2586
213.0145	3.138333	5.73E-08	1.76E-07	22800514.47	18576001.29	1414269.161	25033137.39
376.2209	3.335767	5.48E-08	1.68E-07	3205235.591	2784480.8	83559.32078	3490658.991
266.0644	2.451817	5.39E-08	1.66E-07	560449.2394	561934.4305	1179284.441	662778.1668
393.2281	3.0771	5.14E-08	1.58E-07	1338575.863	3725168.411	386546.7908	994700.743
452.1055	3.145433	4.88E-08	1.51E-07	182454.8594	447655.386	4317.145966	80272.64093
247.9259	0.51826	4.66E-08	1.44E-07	244362.5223	186352.8709	1074814.752	311452.0683
392.2158	3.23855	4.47E-08	1.38E-07	738962.4598	1372856.908	106738.1564	512119.8251
393.1914	2.895275	4.14E-08	1.28E-07	816203.257	1537546.352	211048.9528	732171.0778
271.0993	3.160667	3.90E-08	1.21E-07	1663476.341	774796.6687	200902	619382.1234
474.2622	3.687517	3.36E-08	1.05E-07	656501.6192	568420.2839	1763913.526	518331.8294
351.1811	3.097683	3.31E-08	1.03E-07	1191949.754	2285606.491	184981.2954	867365.5404
464.1246	3.056542	3.30E-08	1.03E-07	2090585.642	275501.5203	218970.6474	2383620.007
206.9881	0.967513	3.20E-08	1.00E-07	1197568.142	1310617.862	2692952.544	1997728.164
147.065	0.96846	2.88E-08	9.01E-08	1329732.803	830926.6646	145431.8663	521015.4356
319.0606	2.808208	2.56E-08	8.01E-08	727554.3121	471555.2277	119557.6344	579566.2888
465.0798	2.859817	2.50E-08	7.85E-08	1074117.656	675797.1412	40047.69362	930678.5507
315.1561	2.65145	2.45E-08	7.69E-08	598149.6654	578442.787	1379321.831	702766.6255
253.1079	2.50095	2.36E-08	7.42E-08	580240.0587	682819.3784	2746805.167	355778.5148
466.2206	4.380883	2.34E-08	7.37E-08	1367505.238	548369.4273	14069.23695	1700002.737
253.0715	2.3413	2.33E-08	7.34E-08	950126.4789	818519.378	502275.5643	181972.2453
346.9018	0.97827	2.23E-08	7.03E-08	893497.5797	1079858.604	1959567.031	1550109.047
257.0846	3.092192	1.94E-08	6.13E-08	1220533.793	678801.7234	213378.3148	158536.0167
330.9295	0.980937	1.89E-08	5.99E-08	883652.7391	1090172.799	1998413.913	1604099.4
432.2388	3.482408	1.78E-08	5.65E-08	578652.6854	859374.0226	30518.04942	587704.4916
274.0877	2.581183	1.65E-08	5.24E-08	2340341.999	414299.8416	917950.5274	1765390.838
221.0449	3.001183	1.62E-08	5.15E-08	2889205.933	8941692.759	108735.6973	1044330.318
251.0558	2.57965	1.58E-08	5.03E-08	3924287.856	3470428.107	690591.0934	1177909.679
464.1499	3.212033	1.52E-08	4.85E-08	1194630.029	1752330.03	53430.70563	920514.3958
207.0655	2.521067	1.48E-08	4.72E-08	5591306.465	4895953.782	4573184.95	1072560.983

Supplementary B- Kruskal-Wallis significant metabolites negative ionization mode

419.0747	2.85515	1.47E-08	4.72E-08	604310.4657	531523.4502	77243.65634	411494.9171
208.0688	2.518692	1.43E-08	4.60E-08	487226.7872	500382.6689	282901.663	132494.4058
234.992	2.52015	1.32E-08	4.25E-08	572111.5393	714691.6767	144312.2912	439306.2761
258.0981	2.6593	1.30E-08	4.17E-08	5739965.921	6996293.57	13705216.74	8932097.118
493.1752	3.143142	1.14E-08	3.67E-08	280436.2551	2625228.646	13792.453	214316.7615
540.3538	3.90565	1.14E-08	3.67E-08	77000.99224	79223.34188	932474.9451	46765.54312
381.2281	2.774917	1.13E-08	3.64E-08	33229179.3	58715164.2	1076205.642	7781479.811
167.0338	2.615917	1.12E-08	3.62E-08	1366877.995	998411.8637	350460.0566	199076.3054
537.9836	3.420217	1.11E-08	3.60E-08	263340.4836	245406.0939	5507.404003	233266.8816
396.2469	3.121608	1.08E-08	3.49E-08	1733157.055	4861511.32	163924.7106	1501467.549
352.0906	3.077583	1.07E-08	3.47E-08	4721073.811	2622546.799	219385.8964	2405385.457
379.0457	2.712867	9.43E-09	3.07E-08	1009575.715	837244.101	363138.3816	616307.9214
221.7999	2.298883	9.35E-09	3.04E-08	2371402.432	605578.5198	680536.6237	1964992.925
407.2073	3.0231	8.85E-09	2.88E-08	289609.0265	1180984.998	36095.42015	335141.7592
364.0542	3.043	8.57E-09	2.80E-08	7108959.574	2904781.759	438805.4102	2657089.669
429.2254	4.033875	8.12E-09	2.65E-08	478493.9455	1685975.182	9483.754914	244713.7495
103.0023	0.506902	7.74E-09	2.53E-08	407821.0204	338045.523	4653222.852	523433.6319
329.0454	3.423483	7.56E-09	2.48E-08	32783868.51	28498825.67	1446536.409	27841597.28
348.0675	2.694625	6.88E-09	2.26E-08	328064.9956	220593.4539	455934.8489	554287.3938
565.0735	2.859942	6.50E-09	2.14E-08	744657.5674	501241.9064	11613.46491	638476.3701
233.045	2.878117	6.10E-09	2.01E-08	1145843.746	783844.1834	993670.0838	115710.6471
248.0642	2.737942	5.83E-09	1.92E-08	3733844.688	1521420.959	840836.0385	784273.9014
448.1555	3.2106	5.63E-09	1.86E-08	179750.0559	539734.4707	5735.406095	191845.6611
139.0388	2.3364	5.54E-09	1.83E-08	426108.7727	455809.5606	2576979.695	513076.9006
351.0873	2.5361	5.07E-09	1.68E-08	25196426.66	27678711.23	1582546.827	16262688.95
200.9709	0.959902	5.01E-09	1.67E-08	3595165.677	4816222.814	15397958.02	8736707.632
383.0325	3.396783	4.84E-09	1.61E-08	566943.5276	259107.4192	24539.72842	505566.1999
399.2366	4.380667	4.77E-09	1.59E-08	7931638.13	2310795.135	110169.8809	9265054.786
378.2365	3.670667	4.70E-09	1.57E-08	1802363.162	3441677.557	132963.9533	841514.5363
172.9532	0.969733	4.69E-09	1.57E-08	557334.124	593305.6128	1492057.549	1114252.451
395.2435	3.123117	4.36E-09	1.46E-08	9179732.951	24192368.39	1269749.282	8295754.295
269.212	4.790875	4.34E-09	1.45E-08	304535.9648	181481.1339	5970.988329	88083.49066
377.2331	3.638767	4.34E-09	1.45E-08	8876694.858	15206394.58	541688.6913	3871036.888
330.0488	3.422583	3.71E-09	1.25E-08	6642320.775	5558903.057	170160.575	5741028.324

Supplementary B- Kruskal-Wallis significant metabolites negative ionization mode

209.0448	2.5531	3.66E-09	1.23E-08	13445517.18	11299454.74	1362825.157	3119810.438
380.2521	3.356633	3.52E-09	1.19E-08	382427.7245	988160.8579	2601.167078	120073.8877
268.9591	0.974537	2.90E-09	9.81E-09	1323224.771	1614678.381	4071906.416	2685538.83
363.0509	3.042675	2.89E-09	9.76E-09	37034595.63	16474331.45	1203030.504	15815413.91
346.0622	2.702283	2.81E-09	9.52E-09	914575.8532	361942.0314	313206.7118	115736.5724
315.1633	4.695225	2.45E-09	8.32E-09	202456.2489	126884.036	4211.686439	273642.3627
414.2575	3.112608	2.32E-09	7.89E-09	755252.972	1307188.201	21200.57233	248067.2058
565.1196	2.44085	2.28E-09	7.75E-09	227552.3486	177051.8926	1482088.196	210159.0521
795.3747	3.136425	2.20E-09	7.51E-09	228010.4471	4748.744244	1760.764316	80573.09503
337.1656	2.755733	2.20E-09	7.51E-09	1223173.636	3505387.563	39174.21349	964955.4285
459.0618	3.044867	2.13E-09	7.27E-09	244457.7819	20327.16402	37280.71799	801431.8997
387.0257	3.128942	2.08E-09	7.11E-09	5577068.08	1400342.435	538168.8158	6685795.665
327.059	3.1349	2.05E-09	7.04E-09	2658190.732	212841.3857	875731.6122	2820572.302
352.0906	2.527333	2.00E-09	6.88E-09	4931156.242	5094022.398	139310.7146	2943769.238
351.0872	3.079833	1.97E-09	6.76E-09	25420322.24	14075061.93	1381007.812	14179853.46
210.0481	2.549933	1.89E-09	6.51E-09	938494.4654	848806.0639	81328.69357	210485.4096
208.0324	2.641883	1.86E-09	6.42E-09	605365.2889	524782.474	32380.23622	277012.4806
132.8664	3.273742	1.54E-09	5.33E-09	10108314.99	18713068.1	14619653.48	6161191.992
178.9767	0.37839	1.50E-09	5.19E-09	2299591.936	2924067.908	4184445.252	2202745.732
403.0518	2.983467	1.28E-09	4.44E-09	1526467.378	1034738.666	178041.7601	398436.478
136.8617	2.999533	1.28E-09	4.43E-09	2308347.505	3559940.992	5044687.632	2154050.307
546.1765	3.105683	1.25E-09	4.36E-09	292841.3181	3496.28806	6220.759277	377514.0112
417.1877	2.642033	1.23E-09	4.28E-09	1699023.931	91817.7407	697928.3295	1138333.725
170.0811	2.170333	1.11E-09	3.87E-09	402764.072	197453.8448	70426.5712	292023.4109
398.2333	4.380033	1.11E-09	3.87E-09	30397186.16	9995576.723	319757.64	34851906.67
434.2237	3.211317	1.10E-09	3.84E-09	131117.5771	463195.3928	3207.088171	80028.34848
582.1377	3.053433	1.05E-09	3.67E-09	523542.7939	51771.66944	12652.99417	516656.7569
229.0113	2.641883	1.00E-09	3.51E-09	731967.6103	746103.1423	73560.89734	428301.3645
207.0292	2.642333	9.34E-10	3.29E-09	8294953.193	7239485.526	785195.7088	4791567.463
506.2835	3.6221	9.33E-10	3.29E-09	185842.43	750734.6017	4829.512021	172996.5641
361.2383	4.038867	7.93E-10	2.80E-09	10550631.84	31304425.23	148793.5387	6397264.307
270.0487	3.045317	7.77E-10	2.74E-09	100214.8441	450841.3258	515571.058	161664.9154
466.083	2.842183	7.70E-10	2.73E-09	317067.5853	200788.0848	7999.0753	184999.7133
222.0483	3.000517	7.28E-10	2.58E-09	186135.5798	725914.0524	1932.594773	64460.1244

Supplementary B- Kruskal-Wallis significant metabolites negative ionization mode

579.2194	3.212467	6.80E-10	2.41E-09	680558.0818	1730254.961	4746.60095	293757.567
580.2227	3.211033	6.13E-10	2.18E-09	136997.8771	383393.9573	1795.742682	74593.28503
236.7585	2.303417	5.60E-10	2.00E-09	1593833.114	211938.4546	190044.2842	815958.2525
367.2396	3.211883	5.22E-10	1.86E-09	1144466.676	2252719.682	51176.98491	817635.1321
433.1462	2.809217	5.06E-10	1.81E-09	792191.8701	142520.4427	30457.54613	663150.5312
579.2978	3.563942	5.07E-10	1.81E-09	281536.5116	270195.0002	15784.13732	202193.1916
413.2191	3.10645	4.32E-10	1.55E-09	1109501.235	1634533.793	31671.84728	914629.2706
366.2363	3.2135	3.93E-10	1.41E-09	12108176.86	25641098.57	68724.27314	3991114.235
216.1234	2.474342	3.73E-10	1.34E-09	769788.3237	198289.6821	296303.1997	1156470.093
313.0254	2.538075	3.36E-10	1.21E-09	351752.25	237839.8566	78950.19718	454780.3467
362.2417	4.0432	3.04E-10	1.10E-09	2971284.552	11830830.61	20995.16432	1550676.643
191.034	3.060867	2.94E-10	1.06E-09	187298.1517	464133.5997	3531483.427	36186.49752
269.0453	3.040042	2.41E-10	8.71E-10	1903378.955	4015242.154	5434243.776	1883322.915
378.2	3.08985	2.41E-10	8.71E-10	3459645.587	8659346.832	30989.9295	1087021.001
462.1345	3.142683	2.15E-10	7.81E-10	999756.6697	3657155.87	9504.762799	344566.5925
583.3078	3.138125	2.12E-10	7.72E-10	243998.3592	5285.446257	2212.191137	186065.7136
396.9748	2.794683	2.07E-10	7.54E-10	1205901.598	1877639.211	3447630.217	1075792.517
178.0499	2.695442	1.74E-10	6.35E-10	3084082.625	4416632.148	417780.8548	8948081.868
348.0592	3.55865	1.70E-10	6.20E-10	3727095.802	3247981.071	48449.69556	2991016.027
132.8664	2.984633	1.59E-10	5.84E-10	18369179.88	30074417.33	29297631.21	14482407.05
228.9597	2.5964	1.59E-10	5.84E-10	815753.6874	771301.7903	117565.9989	610272.8194
496.0096	2.520467	1.59E-10	5.84E-10	522365.2993	651181.7886	7519.053559	337964.3392
303.1632	4.633583	1.55E-10	5.70E-10	105014.8437	109677.8579	1712.864509	158295.8431
352.0906	2.855625	1.42E-10	5.25E-10	4319321.092	2797346.104	221090.5865	2213342.005
283.1186	2.546942	1.37E-10	5.06E-10	8986412.508	16447880.54	1468198.503	5387375.248
223.7978	2.286208	1.32E-10	4.88E-10	2475935.816	519844.4136	510108.1521	2219716.899
505.2805	3.622233	1.31E-10	4.86E-10	943241.0487	3309547.114	43204.98695	727805.9062
362.1665	3.465542	1.23E-10	4.57E-10	578908.4187	807105.5688	1008319.407	621453.411
351.0873	2.858467	1.22E-10	4.52E-10	22294293.93	14107717.11	777789.5984	13391117.98
305.1005	2.538383	1.21E-10	4.51E-10	966942.6502	1182230.424	154280.6031	443014.2763
371.2049	2.995617	1.15E-10	4.31E-10	77438.42814	109360.9497	528267.9305	445990.346
433.2204	3.211958	1.16E-10	4.31E-10	909398.9741	2455343.963	38495.5998	679068.4401
363.245	4.032142	1.10E-10	4.13E-10	371750.5209	1153756.961	12354.1093	271805.7041
361.2383	4.222467	1.00E-10	3.76E-10	6235412.851	13248318.58	510294.8559	3626592.052

Supplementary B- Kruskal-Wallis significant metabolites negative ionization mode

495.1909	3.211533	9.90E-11	3.72E-10	357403.5515	1119920.303	6234.026696	188893.1247
429.1502	3.465817	8.73E-11	3.29E-10	329586.9751	511164.6429	840909.0168	420299.2495
164.0705	2.452925	8.59E-11	3.24E-10	1322492.928	1497310.085	3500251.831	1961628.414
338.0055	2.965892	8.55E-11	3.23E-10	760830.2686	217381.9252	5349.01512	121486.3276
251.0727	2.820267	8.25E-11	3.12E-10	1695042.178	89813.59859	487939.2797	1709301.007
365.233	3.214017	7.79E-11	2.95E-10	59352035.25	115820730.7	384453.6199	23193586.88
289.0716	3.358717	7.62E-11	2.89E-10	37724991.19	33799695.25	1059341.783	1773418.069
533.2728	3.384383	6.72E-11	2.56E-10	313991.0438	491889.9975	5208.209988	137011.4261
347.2226	3.916567	5.95E-11	2.27E-10	36017120.04	72396092.61	706870.0974	16841845.94
465.0965	2.816425	4.79E-11	1.83E-10	1095634.217	882129.7933	25157.71963	782954.7671
419.0747	2.526183	4.78E-11	1.83E-10	999025.7428	1101192.932	33777.43507	579520.0805
323.1499	2.914067	4.26E-11	1.63E-10	3668033.772	6007248.744	354186.7925	2219890.707
377.1967	3.09465	4.21E-11	1.61E-10	20555717.33	41479867.75	425581.0927	8191585.816
125.023	2.234925	3.97E-11	1.52E-10	13356920.26	8358703.113	3252796.456	1825615.46
272.231	4.549233	3.90E-11	1.50E-10	460426.6666	515571.9365	39182.95098	371082.8168
577.2037	3.142175	3.85E-11	1.49E-10	520574.7771	1694080.574	10549.24049	249209.4797
369.1916	2.841833	3.21E-11	1.24E-10	800899.8225	1196575.365	200155.2172	508729.6952
365.2239	3.139467	3.18E-11	1.23E-10	3073483.225	5937985.566	67689.61291	1283575.944
194.0813	3.667075	2.75E-11	1.07E-10	2945874.284	1536107.073	1693945.109	2253888.394
515.1282	2.5913	2.73E-11	1.06E-10	444346.1724	10722.25908	33400.73925	218147.9235
465.1724	2.597583	2.70E-11	1.05E-10	798804.4615	113227.1715	54958.36366	526551.3851
403.285	4.476733	2.66E-11	1.04E-10	481875.8875	693924.756	1503.057595	124163.6803
379.2124	3.158158	2.55E-11	9.97E-11	20149495.01	41398108.28	645352.6871	9171104.17
242.0611	2.50315	2.50E-11	9.79E-11	709989.1204	279859.3231	211236.362	565028.4574
361.1632	3.4676	2.29E-11	8.97E-11	3574421.076	5055786.913	6265454.884	3949684.469
413.2542	3.107842	2.23E-11	8.76E-11	5592197.276	6151738.833	162023.4867	2364976.944
305.0665	3.193117	2.21E-11	8.70E-11	1814824.541	1436489.99	243345.4601	345750.271
317.114	2.517017	2.11E-11	8.32E-11	1691295.821	1300912.533	558031.3022	322355.7154
380.2157	3.15715	1.93E-11	7.62E-11	3919882.798	8096732.378	62277.83981	1363897.563
363.2176	3.1328	1.86E-11	7.34E-11	123219289.3	278960917	612286.4905	32351506.69
364.2209	3.137375	1.59E-11	6.29E-11	25857933	62733012.32	74961.66751	7477690.091
192.0657	2.545367	1.52E-11	6.01E-11	1130495.552	1586727.184	3947692.574	3025147.449
501.1337	2.8167	1.42E-11	5.63E-11	256821.3595	8451.014573	10656.95182	290285.2638
256.05	2.410575	1.22E-11	4.87E-11	864064.2309	249623.9613	228301.0251	620686.8506

Supplementary B- Kruskal-Wallis significant metabolites negative ionization mode

467.0977	2.48845	1.19E-11	4.76E-11	1410352.979	1017376.762	426683.6768	35463.86209
367.2396	3.5098	1.14E-11	4.55E-11	781872.3566	1374436.993	13735.9226	385766.2169
161.0443	1.88565	1.06E-11	4.25E-11	631328.4177	585495.2548	1234115.879	408379.8254
595.1968	3.045483	1.05E-11	4.22E-11	175970.824	6954.19749	2049.638826	477238.5286
584.3201	3.209717	1.02E-11	4.10E-11	275922.5207	3716.74289	1376.105156	102086.7446
382.942	3.381908	8.93E-12	3.60E-11	585810.0561	302797.6516	1767.556673	84966.08826
271.2277	4.55525	8.84E-12	3.57E-11	4119549.078	4264783.901	772295.2655	3400773.583
398.2626	3.89145	6.87E-12	2.78E-11	434287.0469	837191.6539	12234.09148	297525.4228
431.0415	2.864425	6.79E-12	2.75E-11	1380080.872	777392.0057	534253.5728	483143.745
431.098	3.150917	6.28E-12	2.55E-11	1761405.458	1582557.427	42252.544	57814.85063
395.2435	3.473775	6.16E-12	2.50E-11	2146401.903	4455359.265	202411.807	1438551.938
348.2259	3.913917	5.74E-12	2.33E-11	7580871.353	15774613.24	26581.4974	3390157.781
397.2592	3.89495	4.96E-12	2.02E-11	2153616.288	4011420.113	140605.821	1569386.824
285.0766	3.6574	4.65E-12	1.90E-11	1598985.648	321997.9309	73787.62932	610444.2844
313.0702	2.597242	4.42E-12	1.81E-11	778186.6684	137217.6053	271431.8479	554727.4379
389.1968	3.4207	4.31E-12	1.77E-11	487433.9486	755159.2003	31090.36645	274380.7253
582.3044	3.137017	4.20E-12	1.72E-11	964468.8736	14737.87479	1800.933379	389897.6829
391.2125	3.243092	4.18E-12	1.72E-11	3776582.18	6826859.807	115963.7932	2359388.271
539.9993	3.3777	2.69E-12	1.11E-11	420239.6696	277309.8192	1708.210501	425808.2573
413.2542	3.490225	2.62E-12	1.08E-11	1316051.968	1763679.848	12889.12209	656768.6684
447.0667	3.108075	2.37E-12	9.79E-12	1406247.857	711432.5033	88259.39301	162491.2573
433.2591	3.257442	2.35E-12	9.76E-12	607695.2155	1772012.21	1551.343308	208415.2825
510.0936	2.8081	2.34E-12	9.73E-12	262048.0371	18372.16413	5641.155255	150792.6056
394.2312	3.458925	2.11E-12	8.80E-12	746292.1372	1509934.158	7529.808433	352828.3128
158.0811	2.448567	2.08E-12	8.66E-12	2132019.95	3433967.301	8720686.067	6846783.349
373.2018	3.495217	2.07E-12	8.65E-12	4569770.45	3654125.49	123872.4396	4222642.591
358.1506	2.535875	2.02E-12	8.45E-12	363364.7865	5420.558799	38281.72317	632635.2512
461.1777	3.091667	1.80E-12	7.54E-12	2815901.492	355176.4127	67254.23955	2639598.691
179.0703	2.889042	1.74E-12	7.33E-12	20215.36277	21110.82697	2877813.343	22748.74415
392.2158	3.485317	1.54E-12	6.47E-12	470556.2707	1236741.385	7906.905223	152881.607
420.2835	3.3877	1.46E-12	6.17E-12	1653504.194	3702952.859	1481.30536	542816.5422
324.1532	2.90835	1.38E-12	5.83E-12	595428.7456	904552.8482	19123.52649	471285.5148
571.2331	2.81645	1.24E-12	5.25E-12	467868.3088	3128.765604	4325.037208	438979.4707
603.1837	2.8086	1.15E-12	4.86E-12	335543.675	2400.075149	2928.709081	215135.8622

Supplementary B- Kruskal-Wallis significant metabolites negative ionization mode

552.2893	3.64145	1.04E-12	4.42E-12	990974.0073	2100209.742	13783.73518	610377.1945
344.0661	2.537683	9.94E-13	4.24E-12	576478.3296	75167.49932	151592.4204	1244530.306
419.2801	3.39265	9.65E-13	4.12E-12	7738443.297	12242373.92	5430.166576	2340318.951
374.2051	3.494517	8.79E-13	3.76E-12	932171.0444	757983.8201	5379.973144	877107.5206
379.2124	2.877283	8.48E-13	3.63E-12	4696370.917	13204164.94	182744.3249	2898591.198
613.2339	3.136867	8.20E-13	3.52E-12	920665.4794	4384.894695	2617.471514	274540.9266
365.233	3.513417	6.82E-13	2.93E-12	50278236.78	76951674.72	192610.0589	16466805.35
312.0569	2.998242	6.62E-13	2.85E-12	695700.4839	298437.8734	143854.8428	217828.8103
391.2125	3.447883	6.54E-13	2.83E-12	3495503.029	5415098.246	114746.2901	1498681.265
219.102	3.334933	6.25E-13	2.70E-12	74537.05624	77644.7664	201923983.8	66017.76954
363.2176	3.543667	6.06E-13	2.63E-12	38122011.48	113307648.3	358948.0308	13988111.3
418.2678	3.613217	5.57E-13	2.42E-12	515358.7076	1153856.405	2045.80947	108887.4848
251.0558	2.075325	5.53E-13	2.41E-12	2114054.254	1017685.238	119431.1723	61854.04514
431.2047	3.144625	5.46E-13	2.38E-12	1377530.065	4467008.476	186519.8255	1245192.633
480.1449	2.770283	4.67E-13	2.04E-12	510430.6797	660033.1275	12956.98561	83563.46291
447.0929	2.903792	4.51E-13	1.98E-12	442442.2768	447513.9667	26286.00793	37421.08982
429.1891	3.174517	4.38E-13	1.92E-12	329429.696	1848290.171	23461.96993	569684.1814
684.2595	3.578517	4.27E-13	1.88E-12	974144.206	7067.904698	8644.257832	565537.3166
403.0641	2.986667	3.78E-13	1.67E-12	2367421.211	2426297.659	209158.9709	426275.1928
337.0021	2.966025	3.69E-13	1.63E-12	7770921.144	1592824.713	18442.72876	586991.435
366.2363	3.51185	3.56E-13	1.58E-12	10593200.54	16996546.55	15895.96718	3573009.521
529.1649	3.133133	3.53E-13	1.56E-12	748624.1692	46909.27243	13676.29477	988656.9669
419.0052	3.469483	3.50E-13	1.55E-12	1201566.723	552590.9778	3275.352496	90499.56516
353.0224	3.460983	3.43E-13	1.52E-12	965237.2279	311538.3673	3699.197147	61471.28946
364.2209	3.54145	3.37E-13	1.50E-12	7301202.414	24942107.8	26833.1941	3122551.372
446.1141	3.057183	2.65E-13	1.19E-12	3964492.75	2003641.006	21527.76659	2228398.742
189.0547	2.817742	2.31E-13	1.04E-12	1539181.289	991735.5554	95658.93962	286953.8505
249.0765	2.668383	2.31E-13	1.04E-12	4732951.327	1792006.813	470723.5881	394304.732
393.2282	3.468717	2.01E-13	9.03E-13	4181768.576	7988164.192	104042.2476	2163799.531
431.1584	3.062067	1.88E-13	8.45E-13	2495223.317	3604794.591	189663.6687	2607936.203
792.2829	2.808758	1.82E-13	8.22E-13	282139.5858	1206.43806	1554.116094	234419.3184
293.0666	2.3641	1.77E-13	7.99E-13	1123047.974	699579.0946	339472.8816	257928.6272
317.0664	2.576608	1.76E-13	7.96E-13	3148738.635	1953069.189	708607.2486	400310.3808
371.038	3.1058	1.74E-13	7.92E-13	920409.2345	632758.2717	11728.73308	156128.7745

Supplementary B- Kruskal-Wallis significant metabolites negative ionization mode

314.0335	2.538075	1.71E-13	7.79E-13	202907.4005	51886.56891	12847.70162	439804.1465
401.1941	3.15085	1.64E-13	7.48E-13	1562393.068	2344117.949	30699.78413	1148223.474
655.1987	2.808917	1.64E-13	7.48E-13	198903.3462	1573.979074	1928.680699	171354.5525
362.2053	3.177525	1.59E-13	7.27E-13	1373735.819	2891707.429	26807.17337	1182137.558
256.9549	2.597317	1.46E-13	6.70E-13	765164.2723	340703.4693	181286.2403	637360.0497
353.0136	3.469967	1.44E-13	6.64E-13	1962968.17	497833.987	3999.506506	75534.14098
551.2859	3.6729	1.43E-13	6.58E-13	3302586.604	7447308.998	66532.18772	2221673.565
233.0814	2.640317	1.29E-13	5.97E-13	7019103.78	3405805.178	191766.3585	552983.045
267.0597	2.819217	1.28E-13	5.94E-13	1084815.25	156536.4302	377721.7949	1185381.915
446.1394	3.151083	1.20E-13	5.57E-13	1685570.901	1329166.219	14019.0509	475139.9476
295.1549	2.911867	1.05E-13	4.88E-13	1050333.385	1470296.729	64104.35659	683913.4731
417.2645	3.606567	9.66E-14	4.50E-13	2622645.235	5502725.324	3367.270339	564458.9445
307.9623	3.054617	9.22E-14	4.30E-13	13310.91959	340299.2421	1116869.008	61430.91104
592.2248	2.808908	8.46E-14	3.96E-13	423110.2008	3787.004074	2559.420983	327670.499
853.2499	2.808525	7.78E-14	3.65E-13	237661.5083	1305.645663	1617.129996	219653.2937
453.0371	3.0132	7.66E-14	3.60E-13	760840.4151	217446.522	11439.08542	170490.4113
660.0205	3.102958	7.53E-14	3.54E-13	301408.3949	468493.9736	1269.996687	19217.69558
588.2142	2.809525	6.37E-14	3.00E-13	343770.4018	2026.389522	2697.426224	251951.4188
500.1221	3.052733	5.82E-14	2.75E-13	676101.9916	7256.68186	12829.81977	760627.1559
467.0964	2.816183	5.80E-14	2.75E-13	584588.5895	52725.65213	26785.13448	462523.8308
212.0921	2.449242	5.55E-14	2.63E-13	1577573.867	367699.1523	634645.1706	2123606.956
229.9883	1.904408	5.51E-14	2.62E-13	865317.4261	58461.86515	84123.24057	641790.332
382.1269	3.044583	4.58E-14	2.19E-13	1774101.693	30168.81737	53175.06329	3331546.321
361.202	3.184767	4.45E-14	2.13E-13	6821084.821	14965201.37	63082.97047	5580383.89
415.2347	3.124783	4.06E-14	1.95E-13	16985791.4	586089.3842	24431.61834	11600006.2
455.1409	2.816417	3.67E-14	1.76E-13	1008731.959	47856.82637	57223.77542	1023377.812
471.0315	3.106867	3.60E-14	1.73E-13	625211.0551	569387.2701	7920.50366	401253.9475
990.3339	2.809933	3.19E-14	1.54E-13	340191.857	1206.43806	1282.263001	261873.0786
279.0757	2.70225	2.94E-14	1.42E-13	853020.5593	385659.8606	102448.874	90243.86219
383.0311	3.06215	2.83E-14	1.37E-13	817970.4897	77493.39538	99801.87601	1153200.685
315.0046	2.40905	2.82E-14	1.37E-13	675194.759	91877.63177	66173.75562	505295.8197
293.0219	3.095617	2.81E-14	1.37E-13	1079989.8	632950.2832	126751.3398	33973.3201
518.1146	2.81675	2.79E-14	1.36E-13	236772.7762	3001.939238	5685.911464	242449.5683
168.0372	2.16495	2.75E-14	1.34E-13	660185.2351	403544.7147	115461.2606	100361.3067

Supplementary B- Kruskal-Wallis significant metabolites negative ionization mode

337.0929	2.853533	2.62E-14	1.28E-13	1422319.151	454757.2048	143772.4567	107015.2537
352.0212	3.47235	2.49E-14	1.22E-13	7879260.762	1959198.726	4042.289665	321289.7173
417.1612	2.5982	2.29E-14	1.13E-13	2081686.223	63385.78055	536252.4307	1421148.283
230.0578	2.959917	2.25E-14	1.11E-13	968061.5166	241359.0752	64189.29615	22444.23668
677.2617	3.529733	2.25E-14	1.11E-13	706261.8326	10447.77496	17253.74639	358707.5083
532.0757	2.81645	2.21E-14	1.10E-13	358845.3537	8742.731075	5014.352471	339907.3136
152.9242	2.392883	2.04E-14	1.01E-13	1175767.071	165825.1192	130855.3844	892194.3374
211.0605	2.181325	2.02E-14	1.00E-13	957241.5768	645398.7265	120625.9541	154328.0261
257.0452	3.552525	1.96E-14	9.78E-14	27807219.1	13419048.24	500710.3103	2968562.859
321.0612	2.746367	1.94E-14	9.67E-14	2747845.447	1459579.061	346117.9678	113899.1007
271.016	3.547225	1.90E-14	9.54E-14	1308218.271	921790.0018	52796.25799	368330.1116
263.0923	3.03685	1.75E-14	8.78E-14	86111.93869	76952.20111	58349800.66	29596.22806
351.0178	3.473383	1.69E-14	8.52E-14	54862548.43	16068591.57	83710.03374	3371395.232
611.1645	3.044867	1.45E-14	7.33E-14	451178.517	4849.242178	1792.325836	1056773.182
601.1838	2.814325	1.36E-14	6.86E-14	238396.8977	1879.418883	2304.194355	233956.1579
419.9783	3.117583	1.35E-14	6.86E-14	961462.7627	855653.5018	7060.047998	122006.5855
791.2795	2.808758	1.32E-14	6.73E-14	772379.4482	1427.614241	1830.700008	565495.0957
447.9731	3.1166	1.30E-14	6.63E-14	1044037.651	1175923.648	17878.12963	154674.5253
402.9754	3.108733	1.25E-14	6.35E-14	392275.132	1822061.87	7191.990092	47083.88287
512.2625	3.455333	9.25E-15	4.73E-14	447018.0512	7785.934311	1799.721609	328966.8611
166.0861	3.51945	8.71E-15	4.46E-14	1368394.356	45809.31355	47562.38317	753930.7539
577.1323	2.7024	7.21E-15	3.70E-14	2180302.875	254633.5904	350987.2907	41650.6161
388.9677	2.973742	6.16E-15	3.17E-14	2153162.845	1136113.965	15751.47397	165403.331
178.9767	2.598292	5.37E-15	2.77E-14	12193332.65	1633816.518	2088278.409	7296746.259
325.0327	3.108933	5.17E-15	2.67E-14	1316855.988	2990609.317	20847.59976	119916.7649
475.0813	3.137792	4.98E-15	2.58E-14	2346261.288	355407.1804	47825.82799	105202.9696
368.9916	3.532933	4.81E-15	2.50E-14	1710808.858	796315.7684	7307.516759	112929.503
428.9891	2.992133	4.48E-15	2.33E-14	1635547.255	786988.7535	122292.5178	254897.4367
605.1845	2.816817	4.20E-15	2.19E-14	356183.529	2305.534642	3385.230919	392716.2191
238.0692	2.4087	3.75E-15	1.97E-14	919458.2813	125265.5991	37267.61948	692587.4326
399.9887	3.546558	3.53E-15	1.85E-14	1384769.606	554525.1872	13255.4311	477662.76
242.1757	3.053133	3.44E-15	1.81E-14	397185.7272	1565399.963	1816955.295	667852.1675
167.0339	2.164767	3.37E-15	1.78E-14	11685449.98	6722196.198	1891381.774	1232345.662
600.2236	2.81665	3.19E-15	1.68E-14	549167.5729	2750.955521	2840.986184	374327.3016

Supplementary B- Kruskal-Wallis significant metabolites negative ionization mode

403.1449	3.413692	2.96E-15	1.57E-14	868915.718	97456.32452	13502.91335	676508.0559
450.1273	2.81245	2.89E-15	1.54E-14	1333845.629	204584.3582	8459.70513	710854.8351
1141.181	3.5297	2.69E-15	1.43E-14	663868.0435	242683.6463	1206.43806	22011.65416
454.1168	3.064283	2.42E-15	1.29E-14	1464721.231	135468.8645	20280.86822	1581479.345
482.0908	3.055292	2.04E-15	1.09E-14	1488221.55	42822.12876	41293.27989	1518418.197
358.0623	2.98385	1.98E-15	1.06E-14	3953880.781	1470793.731	206462.6368	200980.4156
606.176	2.81675	1.81E-15	9.73E-15	507535.3928	3717.641154	2831.124447	500284.7602
416.2381	3.1229	1.77E-15	9.52E-15	5875895.507	116863.1995	4960.885801	5090979.386
373.0236	2.956433	1.69E-15	9.13E-15	2569888.808	1380627.641	10864.57466	998303.2903
256.0376	3.101408	1.57E-15	8.48E-15	2644945.782	848221.3275	7078.960056	300341.8244
455.976	2.985483	1.55E-15	8.44E-15	962170.3308	774856.1334	22619.07622	311202.6345
305.0665	2.5194	1.49E-15	8.12E-15	2424925.798	2920958.528	262328.1542	606200.8425
330.0441	3.137517	1.36E-15	7.43E-15	4124608.465	1367332.766	24638.66554	1270693.495
389.9601	3.543033	1.32E-15	7.20E-15	645892.7788	233414.366	5293.363179	174775.9177
168.0655	2.598317	1.23E-15	6.74E-15	908009.2431	57874.03551	76889.9699	1165624.007
313.0434	2.825167	1.15E-15	6.31E-15	1251075.591	93092.99953	54361.27746	1121344.366
184.0854	2.809525	1.05E-15	5.80E-15	308554.4534	24279.43602	28131.3692	275268.5092
586.2079	2.809067	9.68E-16	5.34E-15	6975743.327	4778.811228	3916.436003	5330678.928
570.2303	2.814	8.66E-16	4.80E-15	1307122.304	3168.087728	4695.265078	1126081.964
356.9706	3.1004	8.30E-16	4.61E-15	10272970.95	4296593.825	58233.08141	367020.3361
509.1715	3.51225	8.01E-16	4.46E-15	1646227.118	50919.18498	65284.15411	1041378.716
760.1963	3.591017	7.64E-16	4.26E-15	424396.5782	1521.933749	1621.795623	230536.2745
300.0399	2.410675	7.61E-16	4.26E-15	725530.6055	39629.49741	59280.94902	513228.0232
259.0517	3.105008	7.53E-16	4.22E-15	6025768.942	2132495.259	22299.87258	530999.0596
282.0957	2.537833	7.47E-16	4.20E-15	873242.5334	114683.3247	35498.10964	2186744.43
287.056	3.27195	7.29E-16	4.11E-15	46452217.12	13870051.58	1309422.014	1522157.083
631.2638	3.057817	7.03E-16	3.97E-15	926701.0064	3353.99399	2264.321218	848625.7748
346.0237	3.1419	6.88E-16	3.90E-15	706126.3344	36342.18122	37345.10274	1017182.226
285.0752	2.598333	6.33E-16	3.60E-15	1966447.137	296891.4591	260253.3573	1236708.592
654.1954	2.810092	6.06E-16	3.45E-15	692392.1175	1206.43806	1836.29831	604145.0167
651.2464	3.580367	5.99E-16	3.42E-15	485258.2964	64635.097	1680.818089	254451.0089
191.034	2.607167	5.90E-16	3.38E-15	4590738.479	2802500.959	232449.7824	257029.3224
372.9844	3.532317	5.89E-16	3.38E-15	801959.3891	341412.471	2224.374055	59903.18307
445.1308	3.234758	5.90E-16	3.38E-15	2409640.759	648896.0632	25053.93641	46934.81037

Supplementary B- Kruskal-Wallis significant metabolites negative ionization mode

371.9893	3.53495	5.71E-16	3.29E-15	3886470.458	1470169.135	2194.749696	298454.2124
278.1008	3.296217	5.43E-16	3.14E-15	512984.1023	9625.314603	5647.264674	547835.3131
581.2083	2.58985	4.86E-16	2.82E-15	443608.0988	2503.492711	2711.812504	345554.9771
357.059	2.979817	4.77E-16	2.78E-15	29151284.96	10266663.43	1553760.443	1217369.811
599.0233	3.097433	4.74E-16	2.76E-15	1032868.605	333974.2184	1270.135605	23561.35586
864.2339	3.594892	4.44E-16	2.60E-15	230537.8295	1629.456975	1309.773346	202634.7427
449.1507	3.411217	4.35E-16	2.55E-15	800696.7998	57175.47682	15670.8734	658613.8151
448.0147	2.843375	4.26E-16	2.50E-15	468739.373	42757.15199	8152.577582	576636.5628
646.2559	3.414417	3.89E-16	2.29E-15	663186.0162	2255.222868	2187.213933	426408.4958
617.1061	2.94945	3.75E-16	2.22E-15	1214173.925	164359.3374	2176.769404	39453.67444
359.9941	2.702433	3.71E-16	2.20E-15	869316.0291	305485.0977	7446.600477	61940.52263
602.1801	2.808525	3.40E-16	2.02E-15	1225581.44	1445.8102	2927.862086	850861.2782
602.2569	3.4131	3.16E-16	1.88E-15	1067166.833	10339.39197	14557.50791	746442.3274
464.0883	2.8086	3.14E-16	1.87E-15	2162841.678	161505.0499	13006.01407	1457833.793
517.1111	2.816633	2.93E-16	1.76E-15	1377219.897	21668.25728	11289.12177	1318516.912
288.0593	3.270117	2.89E-16	1.73E-15	6586578.362	1682433.58	109409.1999	121014.0607
451.1243	2.702175	2.80E-16	1.69E-15	388367.9796	172664.6562	6666.090519	9337.319573
362.0493	2.598283	2.79E-16	1.68E-15	950381.5822	77184.86759	63627.97632	632908.4636
587.2113	2.808342	2.56E-16	1.55E-15	2415144.538	8036.900374	3289.331999	1713900.933
370.9861	3.538067	2.43E-16	1.47E-15	42307231.54	17287245.04	31688.27105	3444170.347
443.1152	3.245092	2.39E-16	1.45E-15	1765701.778	260153.69	30825.23035	15287.72356
388.1569	2.810733	2.36E-16	1.44E-15	2107837.471	12136.35398	25739.71743	2003852.199
833.3533	3.533967	2.34E-16	1.43E-15	787915.6693	1470.943383	1832.489498	443963.6984
613.1299	3.155533	2.32E-16	1.42E-15	367233.8692	4447.926521	1825.139401	725947.91
642.2611	3.565783	2.24E-16	1.38E-15	862431.7141	1954.232994	2898.865287	497431.72
409.0398	2.96845	2.22E-16	1.37E-15	2234469.89	553381.1956	186740.3788	461165.2066
510.1751	2.598333	2.19E-16	1.35E-15	1602797.371	6788.090577	6644.139938	869544.1694
257.0801	3.524525	2.17E-16	1.35E-15	591287.8658	41916.41315	58329.91164	452422.7954
461.0658	3.009242	2.14E-16	1.33E-15	558226.215	182484.8086	13636.19006	93477.02198
615.2496	3.2043	2.07E-16	1.29E-15	670292.3422	6187.467143	2829.479036	321816.7219
729.2626	3.580175	2.06E-16	1.29E-15	1056208.332	1531.608627	1808.744414	567353.0966
449.124	2.816567	1.78E-16	1.12E-15	5717826.199	351321.8772	36825.74038	4137041.4
435.1714	2.59005	1.72E-16	1.09E-15	885234.8217	39197.9815	72528.27787	612235.8183
543.1292	3.534383	1.72E-16	1.08E-15	1550302.94	551727.4425	4889.364063	77140.64664

Supplementary B- Kruskal-Wallis significant metabolites negative ionization mode

598.0204	3.097433	1.71E-16	1.08E-15	3390851.565	821344.7142	1348.845134	61547.17652
415.1848	3.528267	1.56E-16	9.94E-16	11219860.54	625498.2677	176181.8072	8143884.513
464.1695	3.517808	1.47E-16	9.36E-16	2163661.694	246733.4501	3384.714767	1277895.284
645.1469	2.5982	1.46E-16	9.33E-16	327903.0954	2028.735268	4135.937389	205586.2025
291.0757	2.98285	1.44E-16	9.24E-16	6521515.576	4277726.754	90731.33737	603407.1509
418.0505	2.81715	1.32E-16	8.47E-16	843202.8466	87152.01323	20556.42679	972904.4522
683.2569	3.560483	1.31E-16	8.47E-16	3964077.714	19222.01624	3749.130196	1520172.968
689.0549	3.537825	1.30E-16	8.39E-16	1856167.483	659931.1855	1529.454996	183722.4621
586.2347	3.31695	1.29E-16	8.35E-16	835892.4202	3582.206272	3246.251059	559997.3525
312.1451	3.644792	1.26E-16	8.22E-16	1078562.924	11273.42778	10875.84716	835088.3612
583.1399	2.5973	1.15E-16	7.53E-16	731992.2996	68446.91532	9384.263788	462566.12
583.1047	3.3536	1.15E-16	7.52E-16	1547694.688	309832.9226	3347.021665	17777.48362
915.1181	3.535033	1.10E-16	7.19E-16	1231484.422	340668.533	1266.358297	60080.7634
635.2752	3.061783	1.09E-16	7.16E-16	4309133.848	5897.579907	3071.712913	5426370.393
265.0884	3.101267	1.05E-16	6.94E-16	10860871.76	302702.3828	1225400.465	10544202.94
291.0873	2.858267	1.03E-16	6.84E-16	1414266.569	513012.5039	116477.2696	110701.2727
305.0664	2.963325	1.02E-16	6.81E-16	2481180.736	1311285.435	124686.0625	129467.9959
531.1535	3.51225	9.67E-17	6.44E-16	582454.9871	9361.302523	4604.315817	492211.1471
945.3511	2.589558	9.56E-17	6.38E-16	370608.8662	1206.43806	1206.43806	143940.4299
660.0567	3.538317	9.40E-17	6.30E-16	1195581.104	488124.6012	1670.366571	139180.1655
316.9976	2.490383	9.36E-17	6.29E-16	2351348.628	1633093.453	112012.882	279832.1408
762.1967	3.5865	8.36E-17	5.63E-16	403538.6399	1706.122651	1604.116347	279854.6599
650.2427	3.565167	8.33E-17	5.63E-16	1955869.957	307847.5051	1337.329726	908693.0405
604.1806	2.816558	8.29E-17	5.62E-16	1347443.853	1726.252997	3315.745952	1672260.932
189.0547	2.500625	8.16E-17	5.55E-16	7705823.018	4228381.958	376522.7455	416860.8278
197.0942	3.061717	8.15E-17	5.55E-16	50755259.79	915134.7829	312129.997	29142624.26
246.9907	1.902983	8.08E-17	5.55E-16	1125117.686	192196.6883	169707.1898	735664.8767
489.0423	2.5214	8.15E-17	5.55E-16	831020.3402	541493.6256	11390.68538	21177.94602
661.2751	3.538783	8.16E-17	5.55E-16	1715285.748	61627.46592	1823.708842	1017291.289
601.254	3.410567	7.78E-17	5.38E-16	6283181.373	111782.9213	74372.75833	4747418.603
796.2466	3.581417	7.77E-17	5.38E-16	796867.8804	1811.116603	1982.054174	497710.4084
390.9966	2.98985	7.46E-17	5.19E-16	979494.8288	529472.9534	6229.062612	236777.032
296.0623	2.8178	7.37E-17	5.14E-16	1177458.243	40683.38256	85077.11168	1098921.774
627.0545	3.534825	7.32E-17	5.13E-16	4730732.136	1105547.468	1465.608793	220299.8061

Supplementary B- Kruskal-Wallis significant metabolites negative ionization mode

751.2443	3.590117	7.28E-17	5.11E-16	441849.5931	2090.344459	2025.25591	282825.9368
614.2386	3.137517	7.22E-17	5.08E-16	1199428.762	3125.249721	2772.385552	971316.5188
210.1128	3.296817	7.07E-17	4.99E-16	1983113.008	89132.38834	63483.56656	1867261.409
812.3748	3.506742	6.98E-17	4.95E-16	894307.2936	2476.064192	1578.975418	443641.6007
1140.177	3.531833	6.09E-17	4.33E-16	1467502.377	235310.1182	1206.43806	41631.8104
409.0152	2.765858	5.45E-17	3.89E-16	1130898.814	330203.839	11485.03529	67489.53638
284.0718	3.517167	5.31E-17	3.80E-16	6174295.741	16508.41471	22310.39205	4038398.786
273.0403	3.115967	5.25E-17	3.77E-16	6950233.729	1389236.38	25567.63977	214368.3601
431.9781	3.26325	5.20E-17	3.75E-16	517481.1612	222419.7852	11393.00694	90577.2575
360.0517	1.904217	5.12E-17	3.70E-16	483243.8068	1682.28926	3275.714862	288102.8362
147.065	1.897175	5.06E-17	3.67E-16	34015960.26	2058550.949	3725426.023	22223490.55
263.0559	2.8439	4.85E-17	3.53E-16	1977241.635	584170.8206	151910.8322	165965.152
561.1146	3.142133	4.84E-17	3.53E-16	857277.6679	22607.7845	2288.924319	1005769.002
316.0469	3.063417	4.73E-17	3.46E-16	970664.3661	35954.86568	21429.26324	1332967.3
379.0409	2.9788	4.67E-17	3.44E-16	2121403.685	1826509.03	233290.2909	443547.3902
461.9888	3.547567	4.68E-17	3.44E-16	3365160.932	1909215.173	15293.25161	1097493.934
395.9781	2.501492	4.47E-17	3.30E-16	636657.357	593637.5506	154221.7801	164372.0986
441.0113	2.982867	4.12E-17	3.06E-16	1179800.13	870258.0568	11760.89764	150656.0296
255.0272	2.490383	3.97E-17	2.95E-16	1363962.809	860479.6577	53124.68693	108089.6098
497.1233	3.08595	3.76E-17	2.81E-16	1266591.698	80993.9446	10183.83935	1296379.188
387.1536	2.8167	3.62E-17	2.71E-16	12477260.09	89986.78382	119156.1974	11375825.96
215.053	1.903233	3.56E-17	2.69E-16	1718521.182	136106.8312	183906.9518	1142449.351
565.1857	2.81465	3.57E-17	2.69E-16	639114.1703	231689.0595	3213.490344	10614.59238
148.0683	1.903975	3.52E-17	2.67E-16	1352193.903	92303.49137	110060.5771	791295.0302
433.9937	3.549883	3.51E-17	2.67E-16	2369212.168	1220522.829	5107.984561	482905.0656
641.2239	3.0533	3.52E-17	2.67E-16	3783891.071	65108.39763	2295.001433	2508384.267
394.0428	3.162133	3.19E-17	2.44E-16	6010319.145	382762.4329	177428.7989	6054235.405
750.2409	3.5867	3.00E-17	2.31E-16	1689318.747	2994.279383	3958.396824	1119500.68
517.104	3.095617	2.95E-17	2.28E-16	2520091.451	812805.0295	2328.45839	35650.33886
282.0069	2.8175	2.85E-17	2.21E-16	503738.5033	54248.23186	42788.14407	450786.1986
490.1242	3.057142	2.85E-17	2.21E-16	2400397.344	26562.33934	16332.45528	2205018.559
515.0977	3.097683	2.84E-17	2.21E-16	52255923.69	10202925.89	4987.505506	1047917.225
617.2747	3.525492	2.82E-17	2.21E-16	1324897.509	4999.683592	2431.273784	711794.9635
342.0279	3.1366	2.80E-17	2.20E-16	3281824.937	253077.3017	117038.8748	4393064.977

Supplementary B- Kruskal-Wallis significant metabolites negative ionization mode

811.3714	3.508867	2.81E-17	2.20E-16	2488558.88	3242.014723	1389.107944	1153686.108
610.1613	3.044867	2.78E-17	2.20E-16	2238215.617	3985.321497	1293.322023	3835646.931
258.0485	3.106292	2.61E-17	2.07E-16	209225535	75469656.98	451735.7118	18097352.74
276.0592	3.184083	2.51E-17	2.00E-16	2920108.699	969821.4295	103937.6498	69358.67468
312.0667	3.51865	2.52E-17	2.00E-16	2287433.693	16374.42188	4166.574863	1124306.436
257.0452	3.107967	2.43E-17	1.95E-16	1385667361	508881279.1	3985573.57	135758062.9
849.3484	3.05865	2.40E-17	1.93E-16	3608430.524	2626.859035	1800.361365	4761480.225
250.0693	2.817867	2.36E-17	1.91E-16	20029447.03	132773.7197	285481.2491	18083790.45
277.024	1.902333	2.32E-17	1.88E-16	3630874.878	352412.8763	415223.9847	2412600.693
910.3159	3.071267	2.32E-17	1.88E-16	547094.6346	2712.504891	1543.382868	945878.1236
310.0781	3.140583	2.25E-17	1.84E-16	2646372.004	70759.89348	114684.3693	2730247.63
532.1572	2.597717	2.26E-17	1.84E-16	561772.0287	2909.603896	8510.191785	337522.9208
626.0516	3.535667	2.21E-17	1.82E-16	12517287.07	3094747.706	1206.43806	625630.7217
326.0556	3.121792	2.18E-17	1.80E-16	28775983.12	1795679.334	873858.918	26598905.4
281.0752	3.061717	2.16E-17	1.79E-16	4201076.814	138307.8847	205429.8941	5476788.974
299.0095	2.817567	2.06E-17	1.72E-16	1340588.813	21788.33791	26040.82183	1256575.282
284.0719	2.598417	2.05E-17	1.71E-16	21450458.42	2589177.721	614176.7231	12357500.02
406.9818	1.903983	1.98E-17	1.67E-16	418262.3467	38443.90414	40742.10157	312243.3287
704.1669	3.096783	1.99E-17	1.67E-16	1402594.134	5842.799228	2834.743429	204496.0333
449.1508	2.510117	1.93E-17	1.64E-16	1754536.441	5785.252036	23623.5302	1256880.992
688.0516	3.538283	1.94E-17	1.64E-16	5421242.883	1947926.662	1460.675272	514197.4774
254.0561	3.064425	1.90E-17	1.62E-16	5801661.694	169988.111	180616.7959	7594345.858
434.9969	2.98225	1.82E-17	1.56E-16	733171.1901	406255.2759	6046.153001	41919.04939
745.1406	2.598433	1.81E-17	1.55E-16	466588.4441	3812.720125	1328.525396	295377.6308
410.0156	3.1701	1.75E-17	1.50E-16	1653729.734	40920.21965	23079.85314	1781286.558
570.1048	3.465817	1.66E-17	1.44E-16	311414.1762	2428.97493	1679.119495	28804.48303
645.1362	3.070183	1.66E-17	1.44E-16	903365.1164	7610.025269	5430.420243	1266467.23
1045.437	3.054267	1.66E-17	1.44E-16	863325.6409	2247.027063	1402.516358	1140957.218
668.238	3.40955	1.63E-17	1.42E-16	1777688.938	13851.7888	2051.235466	1364556.11
246.0848	2.9655	1.61E-17	1.41E-16	2410204.048	505987.8235	28090.36236	21358.50496
462.0305	3.204383	1.57E-17	1.39E-16	869304.0574	28982.07529	10643.51631	1132091.832
435.1713	3.53385	1.54E-17	1.36E-16	2746908.786	28426.06476	7445.067198	1937685.019
503.159	2.5903	1.53E-17	1.36E-16	546401.801	34986.14513	52124.43089	458224.1211
456.9821	3.696042	1.52E-17	1.36E-16	440437.9118	153174.0714	4576.198748	12379.40846

Supplementary B- Kruskal-Wallis significant metabolites negative ionization mode

395.9999	2.825958	1.50E-17	1.34E-16	524772.0275	51091.13081	40943.11953	542688.9613
332.9704	2.489933	1.45E-17	1.30E-16	984575.2608	635053.9227	21263.37529	67444.22003
194.0812	3.0813	1.40E-17	1.26E-16	2617927.356	133319.0974	190663.8594	2449077.843
334.0543	2.597975	1.38E-17	1.26E-16	775643.5801	23598.73282	61292.69233	546410.5806
578.1627	2.596883	1.40E-17	1.26E-16	474869.3088	2123.848758	6716.169619	296598.9397
600.2505	3.413142	1.39E-17	1.26E-16	20076744.55	6355.642022	3079.006218	14073776.15
652.2392	3.58305	1.38E-17	1.26E-16	459619.6672	6469.142639	1394.346861	300702.8884
213.0954	2.70945	1.36E-17	1.25E-16	953340.8488	81157.66241	73994.43981	1102117.577
1076.384	3.05795	1.34E-17	1.24E-16	787919.7851	2196.175421	1206.43806	817272.6677
492.1198	3.532025	1.31E-17	1.22E-16	4369228.065	55146.38014	14978.25922	2313879.793
914.1147	3.53545	1.30E-17	1.22E-16	2566716.096	698406.1545	1206.43806	123783.2393
327.0045	2.8178	1.24E-17	1.17E-16	3724309.74	33687.31574	69233.74689	3493569.067
376.9966	2.702958	1.25E-17	1.17E-16	3315899.086	1082459.182	33395.82647	211076.094
516.1011	3.097367	1.20E-17	1.14E-16	15422444.71	4690805.844	4422.218983	301695.047
672.2249	3.5831	1.15E-17	1.09E-16	710058.8834	1905.46614	1398.818503	363657.454
417.1607	3.5346	1.11E-17	1.06E-16	5633456.714	14005.28251	22928.75063	4921988.275
436.1748	3.552383	1.11E-17	1.06E-16	708793.4024	3504.913676	2867.195469	397187.4878
273.0673	3.548083	1.08E-17	1.05E-16	7109445.076	4047575.587	48683.41442	1165518.176
330.0127	2.817417	1.09E-17	1.05E-16	750472.8837	10563.14103	13733.32404	786411.1552
441.9978	2.832267	1.09E-17	1.05E-16	693220.1342	13101.01618	7100.147987	677933.021
685.0848	2.98225	1.04E-17	1.02E-16	654052.6379	215527.3484	1586.833951	6955.450831
210.0762	3.053683	1.01E-17	9.93E-17	2966212.908	34370.58675	110163.5907	2224521.03
677.1529	2.597225	9.97E-18	9.83E-17	386687.5241	2514.657483	1513.138685	198562.8977
616.2722	3.527783	9.70E-18	9.60E-17	9264307.559	5330.94295	5142.130892	5534130.563
345.0589	2.70285	9.52E-18	9.46E-17	6198447.672	2055235.381	100781.4654	362894.9461
587.1361	3.345825	9.40E-18	9.40E-17	3707759.079	550655.9961	2376.481426	2040.88591
862.3053	3.054617	9.22E-18	9.25E-17	1961378.914	2692.577684	1588.876667	1734432.397
606.1127	3.121783	9.10E-18	9.17E-17	849695.6025	30229.7526	1847.616249	1170001.718
416.9912	3.541958	8.96E-18	9.15E-17	2713913.979	1625881.353	4242.107065	399465.8066
418.164	3.529517	9.03E-18	9.15E-17	1684394.132	8485.411844	6099.480034	1336937.753
435.9731	2.7392	9.03E-18	9.15E-17	648013.6233	192360.0801	8848.908722	14603.77521
296.0226	3.053583	8.72E-18	8.95E-17	1931097.627	25251.29287	17151.38359	1622112.518
312.0668	2.598367	8.42E-18	8.69E-17	8049439.316	90895.77982	247700.5214	4598106.914
317.9458	2.827017	8.42E-18	8.69E-17	738213.6856	25495.38985	16523.13641	663756.655

Supplementary B- Kruskal-Wallis significant metabolites negative ionization mode

321.9997	2.81765	8.27E-18	8.62E-17	613346.0643	9338.620398	6155.829276	622164.9177
646.2274	3.059925	8.19E-18	8.58E-17	30219168.3	12187.05828	2706.551292	25952632.28
212.0921	2.711725	7.89E-18	8.35E-17	13660009.4	603979.4395	511112.3735	16320002.33
256.0769	2.5984	7.94E-18	8.35E-17	26516366.54	152182.6662	289912.8139	15013061.84
647.231	3.059283	7.89E-18	8.35E-17	9269232.929	4721.062677	2762.129056	8109006.902
924.1517	2.965017	7.91E-18	8.35E-17	759961.5379	106223.7362	1206.43806	1206.43806
1044.435	3.056617	7.80E-18	8.35E-17	1635493.091	2746.59074	1497.663441	1838966.289
358.039	3.247567	7.31E-18	7.88E-17	1087467.79	239577.004	2134.026114	57387.8463
615.2693	3.53435	7.22E-18	7.82E-17	36893170.43	6105.729477	2302.937302	24575477.12
660.2716	3.54365	7.08E-18	7.70E-17	5008254.42	6097.552182	2203.644332	2900010.893
264.0851	3.096667	6.94E-18	7.66E-17	117726568.5	3697068.532	2084495.938	119719499.1
312.04	2.823517	6.98E-18	7.66E-17	12707934.39	58740.62423	86026.54088	12213050.08
495.1278	3.079925	6.91E-18	7.66E-17	4135052.815	24504.5083	39960.82927	4282493.985
732.2021	3.587617	7.00E-18	7.66E-17	1011564.651	1679.895908	1497.217859	626778.194
509.1716	2.5984	6.81E-18	7.60E-17	7771641.409	22226.83039	26421.98565	4328712.059
531.1537	2.598333	6.80E-18	7.60E-17	2702412.516	7284.417164	13792.0468	1688215.926
728.2592	3.5788	6.77E-18	7.60E-17	2561632.085	2451.84602	1451.73396	1175168.428
313.0253	3.099817	6.47E-18	7.33E-17	6040823.033	148602.3869	89634.67685	7561924.731
464.1697	2.5984	6.45E-18	7.33E-17	6831110.736	30041.15033	46290.56612	3922301.167
657.1612	2.998033	6.34E-18	7.26E-17	1931869.058	157518.0149	1809.545492	72846.57451
413.0463	2.701192	6.28E-18	7.22E-17	572531.1943	301131.6742	15033.56878	41873.04519
642.2118	3.04835	6.14E-18	7.10E-17	2914254.246	17807.86667	1962.962747	1892977.133
183.0845	2.818325	6.04E-18	7.06E-17	9275026.972	79445.64843	56418.16589	8133510.008
539.1284	3.060575	6.00E-18	7.06E-17	1027115.477	9803.537965	4355.555806	1121007.722
657.1986	3.053133	6.07E-18	7.06E-17	1597309.068	20357.51449	1559.859024	895138.7616
675.209	3.053317	6.07E-18	7.06E-17	2740809.578	3481.494918	1846.895877	1740156.579
328.0123	2.8176	5.87E-18	6.97E-17	1971542.666	6603.941349	9914.959215	1812129.927
256.0768	3.515075	5.83E-18	6.96E-17	7012547.349	8151.070139	2507.267447	4454479.948
492.1196	3.058667	5.79E-18	6.96E-17	46672654.73	41113.181	12909.119	46051795.8
546.146	3.127525	5.70E-18	6.88E-17	949633.3963	167347.8587	4826.059846	1303366.344
264.0852	3.544217	5.52E-18	6.84E-17	36928137.21	513938.9285	241966.4044	31898412.78
315.9488	2.826667	5.64E-18	6.84E-17	573051.0074	5086.171423	2275.497004	522248.9304
380.0274	2.826683	5.59E-18	6.84E-17	2589286.072	59810.76326	22992.32077	2725760.056
405.9989	2.984375	5.64E-18	6.84E-17	1198326.591	466790.7199	8923.073329	44533.12851

Supplementary B- Kruskal-Wallis significant metabolites negative ionization mode

468.056	3.112083	5.56E-18	6.84E-17	904259.6334	463820.2705	3084.662342	45846.47641
416.1881	3.059842	5.46E-18	6.82E-17	58059048.18	200838.3936	21004.37925	57951282.87
256.0532	3.0859	5.40E-18	6.79E-17	1688056.297	41396.68297	55709.31743	2019257.116
493.1227	3.064425	5.31E-18	6.71E-17	6943631.656	55588.17228	17766.8409	6313182.537
231.0658	2.71675	5.24E-18	6.66E-17	39092741.75	17821314.77	693724.2625	1548615.981
287.056	2.987317	5.23E-18	6.66E-17	60352954.65	23528735.52	910665.3676	1625506.153
680.2647	3.061233	5.09E-18	6.55E-17	1572474.964	4060.813736	2152.614392	1993939.214
909.3126	3.070083	5.10E-18	6.55E-17	2122480.156	2659.86576	1999.305422	2814574.427
182.0812	2.822583	4.97E-18	6.49E-17	115710514.3	428469.0164	213561.7073	100446416
196.097	3.51155	4.92E-18	6.49E-17	168619157.6	2045393.522	734715.226	154090384.7
274.0681	3.542417	4.98E-18	6.49E-17	1439283.522	769480.6673	2581.038609	461991.817
417.1911	3.061117	4.99E-18	6.49E-17	4716032.36	92088.22375	92056.00747	5291719.032
166.0861	3.053583	4.85E-18	6.46E-17	4796201.582	15740.57435	11358.09263	3495631.852
318.0568	2.817933	4.82E-18	6.46E-17	2450206.519	31040.05862	97696.99768	3040637.149
240.0404	2.819425	4.77E-18	6.43E-17	1403733.853	4114.953844	1966.640161	1285119.391
388.9947	3.697167	4.72E-18	6.41E-17	1066997.922	765999.9185	129172.2472	195169.6205
463.1665	3.522483	4.70E-18	6.41E-17	10070539.39	190969.3419	7576.69425	6505726.254
577.1592	2.5984	4.69E-18	6.41E-17	2218487.393	4430.850716	4676.002348	1383065.223
415.1847	3.05825	4.59E-18	6.35E-17	287795569.1	996038.2575	90719.76797	282507814.2
538.125	3.060267	4.50E-18	6.26E-17	6288166.414	29377.96304	2931.721929	6309728.505
697.2456	3.061717	4.34E-18	6.07E-17	3884813.114	7910.463697	7680.413681	4851689.063
840.3243	3.05215	4.30E-18	6.06E-17	5447245.862	1536.352945	1698.449261	3338234.554
649.2261	3.055983	4.24E-18	6.01E-17	3416024.844	5061.977572	4563.082892	2797998.797
868.3132	3.05445	4.11E-18	5.86E-17	850785.677	1993.155008	1309.301005	1227892.903
404.0014	2.738617	4.02E-18	5.78E-17	564789.4774	140880.5421	4751.574622	25422.72095
711.2065	3.063458	4.02E-18	5.78E-17	2265675.022	13364.38293	1927.0907	2780138.392
272.0644	3.541433	3.96E-18	5.77E-17	299165305.3	153595552.2	1017504.707	45227476.35
343.0432	3.18425	3.95E-18	5.77E-17	804274.1297	830021.5777	16816.00708	268742.4475
712.2139	3.065817	3.91E-18	5.76E-17	1994097.506	8385.93668	2669.840717	2330290.024
864.3172	3.053492	3.87E-18	5.75E-17	1939550.942	2169.608648	1574.232315	2112015.454
271.0611	3.543217	3.78E-18	5.69E-17	1815173480	943463862	7370066.822	289733405.6
545.1425	3.095375	3.80E-18	5.69E-17	6244940.459	97749.85767	42781.09268	7720330.56
278.0749	2.710775	3.75E-18	5.68E-17	23772005.89	6418577.181	375080.1376	887999.0042
281.0752	3.541583	3.74E-18	5.68E-17	4001880.29	31145.31066	21418.06399	3112024.157

Supplementary B- Kruskal-Wallis significant metabolites negative ionization mode

275.0561	2.739258	3.63E-18	5.58E-17	10007686.55	3775512.602	389253.2267	572078.9349
633.0657	2.966358	3.58E-18	5.58E-17	4062608.81	424367.7349	1972.715356	6855.635153
634.2717	3.061717	3.61E-18	5.58E-17	14938996.83	4956.568842	2921.593638	17180633.29
1062.418	3.061317	3.58E-18	5.58E-17	833009.5839	2270.917374	1206.43806	1178156.736
478.1584	3.07985	3.50E-18	5.54E-17	6173296.02	108811.9508	7498.555401	5825483.117
614.2659	3.53315	3.45E-18	5.49E-17	153016108.7	5187.065145	3213.971391	91052810.63
632.2475	3.060967	3.42E-18	5.48E-17	3137618.491	2957.30521	2644.772361	2805660.75
369.0084	3.697783	3.33E-18	5.38E-17	1151355.996	304320.8034	12912.877	45668.57251
644.2229	3.551425	3.27E-18	5.33E-17	2429777.252	2177.469322	2076.569452	1589609.332
915.3295	3.0618	3.23E-18	5.29E-17	935402.231	2380.692694	1467.821391	1360303.245
198.101	3.056033	3.18E-18	5.26E-17	8198621.946	27592.34499	20992.77993	6968409.057
301.0326	2.48715	3.14E-18	5.24E-17	1522405.602	1172145.576	58936.95713	159477.6076
408.0327	2.702117	3.15E-18	5.24E-17	537407.6183	165852.4012	5042.626072	12009.3836
641.0719	2.959608	3.06E-18	5.24E-17	846337.3845	127047.7781	1392.645156	1884.004329
832.3674	3.053667	3.13E-18	5.24E-17	3477218.723	2544.307404	1528.043633	3341627.524
841.3277	3.049383	3.10E-18	5.24E-17	2554624.23	1373.862549	1460.956529	1518008.136
341.02	3.111917	2.99E-18	5.19E-17	18679224.13	776744.4287	138383.0626	19785610.9
1079.388	3.054883	3.00E-18	5.19E-17	726010.9773	2627.597401	1330.766548	1318256.415
344.0282	3.129633	2.85E-18	5.01E-17	3001990.011	284764.0125	45088.34493	3678067.698
418.9708	2.744817	2.77E-18	4.91E-17	1963629.069	418620.7031	6216.518947	39790.74479
696.2422	3.060967	2.75E-18	4.91E-17	13263562.02	6657.097199	1689.776585	15495818.38
847.3418	3.060617	2.76E-18	4.91E-17	39008892.02	4450.335012	1814.63461	43491102.62
410.0631	2.860867	2.70E-18	4.90E-17	1661860.429	307814.2801	15484.28676	43782.50288
463.1666	2.598433	2.67E-18	4.90E-17	32510389.18	68299.75509	40989.5359	18640423.51
483.172	3.062233	2.66E-18	4.90E-17	13530123.23	160124.9546	41669.61951	16051004.68
628.2546	3.060267	2.58E-18	4.81E-17	187717195.9	9165.440845	1736.159761	158683416
198.1035	3.057183	2.44E-18	4.74E-17	6865822.822	28187.97459	23563.3493	5475646.4
367.0127	3.701883	2.51E-18	4.74E-17	33784782.12	7132153.818	59114.69106	837044.2496
435	3.697225	2.52E-18	4.74E-17	572785.4833	119714.1045	1833.719753	14863.18073
447.0317	2.4926	2.50E-18	4.74E-17	1222721.899	672203.6581	16899.21703	52893.72866
629.2582	3.05965	2.36E-18	4.74E-17	59609781.58	8505.223725	1600.826266	50411717.3
642.2312	3.053317	2.43E-18	4.74E-17	3063728.384	19129.78286	2269.076433	2021772.45
643.2192	3.053625	2.49E-18	4.74E-17	59953073.08	10639.43506	2316.051635	45740586.39
831.3645	3.053967	2.41E-18	4.74E-17	6655318.538	2704.780677	1206.43806	6691489.822

Supplementary B- Kruskal-Wallis significant metabolites negative ionization mode

863.3136	3.053667	2.38E-18	4.74E-17	5224808.264	2762.59771	1747.978968	5803147.182
431.055	3.016167	2.24E-18	4.65E-17	9206493.806	1764418.92	42400.3307	771867.0217
630.2613	3.059117	2.25E-18	4.65E-17	10870774.84	4166.43734	1617.197128	9290826.269
651.2223	3.050308	2.29E-18	4.65E-17	865712.7758	1460.433628	1971.348192	749192.2419
1060.412	3.059842	2.27E-18	4.65E-17	8343928.09	3205.431589	1275.713259	9556809.197
682.2534	3.561633	2.20E-18	4.65E-17	11121321.75	2598.178842	1695.854087	4181620.661
288.0635	2.97685	2.17E-18	4.63E-17	10002679.72	2695662.184	48624.46107	219806.7608
420.0328	2.984867	2.16E-18	4.63E-17	839583.4518	390398.6148	3400.847552	36742.76962
848.3452	3.060517	2.10E-18	4.58E-17	17112949.36	3663.506286	1584.673377	19219553.08
764.2298	3.063483	2.05E-18	4.52E-17	1213524.432	5618.118615	2228.207535	2566837.473
643.2195	3.55455	2.01E-18	4.47E-17	8200838.168	2081.322212	1701.663572	4855535.74
336.0155	3.061233	1.98E-18	4.45E-17	3081463.601	48402.63483	12158.50022	4538553.938
475.0166	2.702275	1.94E-18	4.39E-17	580315.4856	203649.569	6041.235753	27013.91379
865.3147	3.054358	1.92E-18	4.39E-17	7697276.169	5298.329119	1762.874408	10693254.26
256.0374	3.539933	1.83E-18	4.23E-17	3158595.237	1488144.175	7458.265718	527843.6758
484.1755	3.065042	1.81E-18	4.23E-17	2558306.622	10354.11609	6818.185849	3456455.545
1061.416	3.056983	1.81E-18	4.23E-17	4453050.302	2649.608221	1206.43806	5261387.683
867.3088	3.056842	1.75E-18	4.17E-17	1672474.975	2216.245435	1401.766883	2451402.715
342.031	2.746417	1.70E-18	4.11E-17	1105180.189	304562.738	8000.209239	72133.1399
386.97	2.973192	1.70E-18	4.11E-17	1783761.198	472592.6073	5332.644497	403168.6685
409.0075	3.171033	1.68E-18	4.11E-17	2496952.333	41780.34299	36091.61118	3257345.578
648.2226	3.057142	1.66E-18	4.11E-17	10420064.08	6201.131668	1789.792876	8862009.766
503.0579	2.977675	1.63E-18	4.11E-17	2821918.756	1116953.745	16889.53412	72174.787
744.2239	3.56495	1.62E-18	4.11E-17	758826.0006	2181.488483	1630.992172	340640.2433
477.155	3.073883	1.58E-18	4.09E-17	30183609.07	657316.9909	13216.94812	27164765.7
645.2305	3.053583	1.52E-18	3.97E-17	14635947.72	3256.146635	2565.602336	11582410.52
476.1322	3.1058	1.48E-18	3.92E-17	2722279.954	550129.3506	18261.66019	580366.4385
614.2734	3.075067	1.46E-18	3.91E-17	1743828.204	3067.335095	1445.826057	1199447.854
287.0559	3.5435	1.41E-18	3.88E-17	9702543.597	3935766.57	52135.95854	475965.6403
311.0535	2.979167	1.42E-18	3.88E-17	6674462.258	2535872.578	65653.42847	381219.9528
635.07	2.963633	1.43E-18	3.88E-17	1358723.054	225098.0931	1868.166967	5633.096354
866.318	3.05645	1.43E-18	3.88E-17	3007215.311	2739.408051	1986.126634	4411224.835
197.1003	3.060517	1.36E-18	3.88E-17	292987531.6	954838.8844	393345.843	226246394.8
508.1582	3.044233	1.36E-18	3.88E-17	2460613.319	60033.60746	6833.893233	27370.38963

Supplementary B- Kruskal-Wallis significant metabolites negative ionization mode

714.2151	3.06805	1.32E-18	3.88E-17	911404.5372	3145.394148	1564.988948	1342911.763
196.097	3.0624	1.08E-18	3.87E-17	2786107255	15205226.01	5736091.454	2145801089
245.0814	2.966217	1.29E-18	3.87E-17	20969862.87	5260675.289	124731.7451	233241.6104
275.056	3.18755	1.02E-18	3.87E-17	23575849.74	5995688.712	50980.8735	603232.4958
330.9438	2.487167	1.29E-18	3.87E-17	887983.17	359996.7015	28159.82767	25286.67203
368.0161	3.7008	1.26E-18	3.87E-17	4894555.516	1040589.709	7658.405097	102078.657
388.9966	2.971392	1.25E-18	3.87E-17	13337415.7	3139839.423	28911.67171	197410.1014
393.9989	2.702483	1.30E-18	3.87E-17	843566.503	260620.5192	7374.876845	140543.4978
405.0282	3.014	1.29E-18	3.87E-17	2503753.223	331511.7588	22516.84466	80983.66742
425.0465	2.989567	1.20E-18	3.87E-17	508831.2839	251331.3175	3485.248048	32533.54192
433.9938	2.978617	1.30E-18	3.87E-17	5707419.762	2514956.401	33179.63368	203911.1667
445.0707	3.107283	1.09E-18	3.87E-17	52068298.76	11833031.44	71714.77542	640511.0627
446.0742	3.107283	1.07E-18	3.87E-17	10449929.37	3794824.36	29264.58234	1136646.961
560.1069	3.0948	1.25E-18	3.87E-17	2768819.506	22945.37485	3881.634626	3587697.201
581.1563	2.963283	1.25E-18	3.87E-17	9125839.412	927575.0236	2284.125638	9028.154343
587.1357	2.963325	1.23E-18	3.87E-17	6649931.05	449147.1485	1637.492857	2771.162145
612.2771	3.05825	1.14E-18	3.87E-17	29103447.12	6939.631083	1830.655358	20606371.64
613.28	3.05645	1.04E-18	3.87E-17	11583030.28	10956.07277	1562.09169	8191657.031
644.2263	3.056817	1.16E-18	3.87E-17	49644569.8	5942.081611	2191.574736	36591171.38
470.9853	2.739175	9.76E-19	3.84E-17	1039146.429	301424.4203	4249.537584	27732.76841
554.0749	3.1053	9.51E-19	3.84E-17	1263667.445	10572.71922	4653.523732	98947.54477
624.1176	2.972442	9.78E-19	3.84E-17	1341868.834	288944.5897	2020.224016	8218.281863
234.0484	2.492583	9.12E-19	3.78E-17	10152332.51	4322659.622	47987.95146	457738.8826
233.045	2.500925	8.65E-19	3.66E-17	84957505.76	39971636.73	798228.9498	2442120.486
603.1084	2.964092	8.35E-19	3.60E-17	2232282.861	623135.598	1841.693908	7480.29625
232.0691	2.71925	7.79E-19	3.42E-17	4519705.226	2063198.907	66181.2768	204441.8142
419.0294	2.977508	7.55E-19	3.39E-17	5508965.376	2969835.748	22729.28622	209474.3723
289.0207	2.967108	7.08E-19	3.26E-17	1394201.537	620083.4761	3765.966105	60375.49306
409.0597	2.860775	7.00E-19	3.26E-17	10147685.04	1754785.107	113534.6228	348773.4424
463.9679	2.7458	7.12E-19	3.26E-17	1738378.692	453699.822	11036.8198	63069.28794
1078.385	3.058275	6.92E-19	3.26E-17	2158306.302	2653.971706	1206.43806	2814614.388
319.0457	2.764017	5.77E-19	2.88E-17	58147535.1	13047361.03	67509.59334	1388419.977
494.1434	3.163883	5.30E-19	2.80E-17	1093681.307	10743.47974	3730.041912	250534.8412
632.0616	2.9655	5.40E-19	2.80E-17	13147310.92	1210068.721	2046.736551	11556.64549

Supplementary B- Kruskal-Wallis significant metabolites negative ionization mode

664.1063	2.974608	5.49E-19	2.80E-17	1039529.745	275333.0889	1660.047068	6279.66124
603.1383	2.96655	4.82E-19	2.69E-17	5050581.45	616437.5118	2195.548636	7693.400179
643.231	3.115017	4.89E-19	2.69E-17	8164440.63	9823.078048	2836.215767	1839691.352
289.0716	2.972783	3.17E-19	1.84E-17	1831369830	370447542.5	1639474.763	13819588.72
429.0257	2.96925	2.97E-19	1.84E-17	5859125.357	1550952.206	190999.4718	368306.7664
601.1322	2.966033	3.08E-19	1.84E-17	85472558.36	6598069.247	2560.512197	81915.70334
663.1029	2.972125	3.18E-19	1.84E-17	3481805.601	857734.6359	1903.002923	25802.56761
247.0608	2.745467	2.79E-19	1.81E-17	32480221.5	6259089.489	53383.75162	562572.4332
467.0525	3.107967	2.63E-19	1.75E-17	5762223.701	1775028.098	18366.26729	373168.078
602.1356	2.966117	2.31E-19	1.58E-17	27362209.68	2497527.535	1983.117057	28886.9412
623.1142	2.970283	2.20E-19	1.56E-17	4780974.643	871954.2893	2940.741972	45846.32033
811.1203	3.096783	2.14E-19	1.56E-17	1413956.275	2688.014329	1525.168222	28714.71777
908.1796	2.963517	2.18E-19	1.56E-17	3260077.087	288287.8491	1206.43806	1854.382526
553.0674	3.106067	1.92E-19	1.51E-17	1093528.976	2668.661127	3034.600876	198641.9061
320.049	2.755767	1.55E-19	1.35E-17	10208686.35	1744372.869	7755.814755	204789.9197
407.0295	2.702142	1.62E-19	1.35E-17	3509398.886	1491087.262	51497.61763	175457.7626
421.994	2.702358	1.66E-19	1.35E-17	2481620.575	601213.6953	2983.195803	91231.34603
907.1763	2.9644	1.36E-19	1.24E-17	6873615.704	566652.0417	1206.43806	2718.965273
277.0715	2.718167	1.24E-19	1.23E-17	162360396.8	41091741.48	557375.1931	5368815.945
347.0302	2.997642	1.19E-19	1.23E-17	1149147.171	568426.2364	6184.088456	77216.4369
432.0657	3.049767	1.09E-19	1.23E-17	3029373.094	670130.4893	31023.83695	5632676.465
580.1535	2.963283	1.20E-19	1.23E-17	46608456.92	4548151.474	2188.888128	20083.89591
735.0699	2.967783	1.06E-19	1.23E-17	884375.9393	57635.88371	1540.875048	2531.72694
929.1584	2.966075	1.29E-19	1.23E-17	861729.7136	123804.5697	1206.43806	1476.99535
290.0748	2.969267	6.37E-20	8.69E-18	299230712.9	67346896.63	294403.0053	2718484.989
923.1481	2.963367	6.72E-20	8.69E-18	1998057.603	222208.0713	1206.43806	1297.599231
579.1501	2.96495	5.00E-20	7.33E-18	142938936.6	16768204.29	7934.1136	192086.433
963.223	3.09255	4.65E-20	7.30E-18	1647029.947	1936.740287	1383.264092	32624.28203
402.9981	2.746483	3.67E-20	6.21E-18	4075361.324	918144.5234	6941.059605	96776.48973
599.0729	3.106033	3.50E-20	6.21E-18	1344167.449	34934.24071	1662.057345	346863.6365
408.0474	2.968667	2.53E-20	5.06E-18	10494489.06	1600494.009	15950.94553	244843.7976
341.0276	2.746767	2.00E-20	4.80E-18	10258540.18	2424005.354	14171.16852	470098.7098
721.2287	3.045425	2.07E-20	4.80E-18	2979119.949	8717.01817	2840.188804	7047.367707
750.1725	3.095	2.18E-20	4.80E-18	2459309.263	1959.33412	1666.408282	291299.3949

Supplementary B- Kruskal-Wallis significant metabolites negative ionization mode

407.0439	2.9705	1.29E-20	4.05E-18	60558587.64	10057957.59	64110.65775	1126093.419
570.1289	3.011983	4.44E-21	2.43E-18	774489.4419	25403.40328	2030.956141	12714.13752
887.338	3.540133	6.64E-21	2.43E-18	2680186.814	1610.61247	1587.401594	197179.6653
962.2196	3.093642	6.58E-21	2.43E-18	3120883.696	1503.863225	1206.43806	87755.59232
736.1931	3.047075	1.87E-21	1.37E-18	4823888.979	3421.361979	2125.737807	5187.160787
737.1974	3.0446	7.16E-22	1.34E-18	1599101.723	1917.06368	1446.199464	1902.774331
886.3346	3.541417	1.22E-21	1.34E-18	5931034.552	2215.461524	1678.017677	530261.8047

Supplementary C

Table S1- List of 128 *Alternaria* sp. with host, location of sample collection, chemotype group assignment and phylogenetic species identification based on *RPB*2 sequences.

Specimen Code	RPB2 ID	PCA Group	Host	City, Province
KAS5401	*A. alternata*	1	*Malus domestica*	Vineland, Ontario
KAS5400	*A. arborescens*	4	*Malus domestica*	Vineland, Ontario
KAS5399	*A. arborescens*	2	*Malus domestica*	Vineland, Ontario
KAS5398	*A. arborescens*	1	*Malus domestica*	Vineland, Ontario
KAS5387	*A. arborescens*	3	*Lunaria annua*	Orleans, Ontario
KAS5385	*A. alternata*	4	*Thalictrum* sp.	Orleans, Ontario
KAS5384	*A. alternata*	2	*Begonia* sp.	Orleans, Ontario
KAS5382	*A. alternata*	1	*Ipomea* sp.	Orleans, Ontario
KAS5381	ND	2	*Ipomea* sp.	Orleans, Ontario
KAS5380	ND	1	*Astilbe* sp.	Orleans, Ontario
KAS5379	*A. alternata*	1	Unknown	Ottawa, Ontario
KAS5378	*A. arborescens*	1	*Euchera americana*	Orleans, Ontario
KAS5377	ND	1	*Thuja officinalis*	Nepean, Ontario
KAS5375	*A. alternata*	4	Unknown	Leitrim, Ontario
KAS5373	*A. alternata*	1	*Silene vulgaris*	Ottawa, Ontario
KAS5372	*A. alternata*	2	*Asclepias* sp.	Leitrim, Ontario
KAS5369	*A. alternata*	4	*Linaria vulgaris*	Orleans, Ontario
KAS5368	*A. arborescens*	1	*Daucus carota*	Ottawa, Ontario
KAS5365	*A. arborescens*	1	*Brassica* sp.	Orleans, Ontario
KAS5337	*A. alternata*	2	*Rhus typhina*	Orleans, Ontario
KAS5327	ND	4	*Hibiscus rosa-sinensis*	Orleans, Ontario
KAS5325	*A. alternata*	1	*Scirpus* sp.	Orleans, Ontario
KAS5324	*A. alternata*	1	*Delphinium* sp.	Orleans, Ontario
KAS5322	*A. alternata*	1	*Acer* sp.	Orleans, Ontario
KAS5321	*A. arborescens*	1	*Lycopersicon esculentum*	Orleans, Ontario
KAS5320	*A. alternata*	2	*Daucus carota*	Ottawa, Ontario
KAS5319	*A. alternata*	1	*Sphagnum* sp.	Orleans, Ontario
KAS5313	*A. alternata*	1	*Lycopersicon esculentum*	Ottawa, Ontario
KAS5312	*A. alternata*	4	*Pinus* sp.	Ottawa, Ontario
KAS5309	*A. alternata*	1	*Aesculus flava*	Ottawa, Ontario
KAS5308	*A. alternata*	4	*Malus baccata*	Ottawa, Ontario
KAS5307	*A. arborescens*	1	*Ipomea baccata*	Orleans, Ontario
KAS5305	*A. alternata*	1	*Juniperus virginiana*	Ottawa, Ontario
KAS5304	*A. alternata*	2	*Weigela* sp.	Orleans, Ontario
KAS5303	*A. alternata*	4	*Sedum* sp.	Ottawa, Ontario
KAS5301	ND	1	*Brassica* sp.	Ottawa, Ontario
KAS5300	*A. arborescens*	1	*Tamarix ramoissisima*	Orleans, Ontario
KAS5299	*A. alternata*	2	*Lewisia longipetala*	Orleans, Ontario
KAS5297	ND	2	*Lewisia longipetala*	Orleans, Ontario
KAS5296	*A. alternata*	4	*Salix* sp.	Ottawa, Ontario
KAS5295	*A. alternata*	1	*Hippeastrum* sp.	Ottawa, Ontario
KAS5293	*A. alternata*	1	*Pilosella aurantiaca*	Nepean, Ontario
KAS5287	*A. alternata*	2	*Hedera* sp.	Ottawa, Ontario
KAS5286	*A. arborescens*	2	*Rosa* sp.	Orleans, Ontario
KAS5274	*A. alternata*	1	*Papaver rhoeas*	Orleans, Ontario
TOM2	*A. alternata*	1	*Solanum lycopersicum*	London, Ontario
TOMSTEM3a	*A. alternata*	1	*Solanum lycopersicum*	London, Ontario
TOMSTEM3b	*A. alternata*	1		
TOMSTEM3c	*A. alternata*	1	*Solanum lycopersicum*	London, Ontario
TOMSTEM3d	*A. alternata*	4	*Solanum lycopersicum*	London, Ontario
BBJS14b	ND	4	*Vaccinium* sp.	Vineland, Ontario

JSP4AR5	ND	1	*Vitis vinifera*	Vineland, Ontario
NS1	ND	1	*Vitis vinifera*	Nova Scotia
NSFoch2	ND	1	*Vitis vinifera*	Nova Scotia
MBBC37c	*A. alternata*	2	*Vaccinium angustifolium*	Mt. Thom, Nova Scotia
RJS1-2a	*A. alternata*	1	*Rubus* sp.	Vineland, Ontario
RJS2-2c	*A. arborescens*	1	*Rubus* sp.	Vineland, Ontario
RJS1-4a	*A. arborescens*	1	*Rubus* sp.	Vineland, Ontario
KAS5521	*A. arborescens*	1	*Hordeum vulgare* L. (Canada Eastern)	P.E.I
KAS5520	*A. alternata*	4	*Secale cereale* (Canada Eastern)	Quebec
KAS5519	*A. alternata*	4	*Secale cereale* (Canada Eastern)	Quebec
KAS5518	*A. alternata*	4	*Secale cereale* (Canada Eastern)	Quebec
KAS5517	*A. alternata*	1	*Secale cereale* (Canada Eastern)	Quebec
KAS5516	*A. alternata*	1	*Secale cereale* (Canada Eastern)	Quebec
KAS5515	*A. alternata*	4	*Secale cereale* (Canada Eastern)	Quebec
KAS5514	*A. alternata*	4	*Secale cereale* (Canada Eastern)	Quebec
KAS5513	*A. alternata*	1	*Avena sativa* (Canada Western)	Edmonton, Alberta
KAS5512	*A. alternata*	2	*Triticum* spp. (Amber Durum)	Beechy, Saskatchewan
KAS5511	*A. alternata*	1	*Triticum* spp. (Amber Durum)	Beechy, Saskatchewan
KAS5510	*A. alternata*	1	*Triticum* spp. (Amber Durum)	Beechy, Saskatchewan
KAS5509	*A. alternata*	4	*Triticum* spp. (Amber Durum)	Beechy, Saskatchewan
KAS5508	*A. alternata*	4	*Triticum* spp. (Amber Durum)	Beechy, Saskatchewan
KAS5507	*A. infectoria*	3	*Brassica napus* (Canada Western Canola)	Western Canada
KAS5506	*A. infectoria*	3	*Brassica napus* (Canada Western Canola)	Western Canada
KAS5505	*A. infectoria*	3	*Brassica napus* (Canada Western Canola)	Western Canada
KAS5504	ND	1	*Triticum* spp. (Canadian Eastern Red Winter)	Western Canada
KAS5503	*A. alternata*	2	*Triticum* spp. (Canadian Eastern Red Winter)	Western Canada
KAS5502	ND	3	*Triticum* spp. (Canadian Eastern Red Winter)	Western Canada
KAS5500	*A. alternata*	1	*Triticum* spp. (Canadian Eastern Red Winter)	Western Canada
KAS5499	*A. alternata*	1	*Brassica napus* (Canada Western Canola)	Western Canada
KAS5498	*A. infectoria*	3	*Brassica napus* (Canada Western Canola)	Western Canada

KAS5497	*A. alternata*	4	*Brassica napus* (Canada Western Canola)	Western Canada
KAS5496	*A. alternata*	4	*Hordeum vulgare* L. (Canada Western)	Western Canada
KAS5495	*A. alternata*	4	*Hordeum vulgare* L. (Canada Western)	Western Canada
KAS5494	*A. alternata*	1	*Hordeum vulgare* L. (Canada Western)	Western Canada
KAS5493	*A. alternata*	1	*Hordeum vulgare* L. (Canada Western)	Western Canada
KAS5492	*A. alternata*	1	*Hordeum vulgare* L. (Canada Western)	Western Canada
KAS5491	ND	4	*Hordeum vulgare* L. (Canada Western)	Western Canada
KAS5490	ND	3	*Hordeum vulgare* L. (Canada Western)	Western Canada
KAS5489	*A. alternata*	4	*Hordeum vulgare* L. (Canada Western)	Western Canada
KAS5487	*A. alternata*	2	*Triticum* spp. (Canada Western Amber Durum)	Western Canada
KAS5485	*A. alternata*	1	*Triticum* spp. (Canada Western Amber Durum)	Western Canada
KAS5484	ND	1	*Triticum* spp. (Canada Western Amber Durum)	Western Canada
KAS5483	*A. infectoria*	3	*Triticum* spp. (Canada Western Amber Durum)	Western Canada
KAS5482	*A. infectoria*	3	*Triticum* spp. (Canada Western Amber Durum)	Western Canada
KAS5481	*A. alternata*	4	*Triticum* spp. (Canada Western Amber Durum)	Western Canada
KAS5480	ND	3	*Triticum* spp. (Red)	Western Canada
KAS5479	*A. alternata*	4	*Triticum* spp. (Red)	Western Canada
KAS5478	*A. alternata*	4	*Triticum* spp. (Red)	Western Canada
KAS5477	*A. infectoria*	3	*Triticum* spp. (Red)	Western Canada
KAS5476	*A. infectoria*	3	*Triticum* spp. (Canada Western Amber Durum)	Western Canada
KAS5475	ND	3	*Triticum* spp. (Canada Western Amber Durum)	Western Canada
KAS5474	*A. infectoria*	3	*Triticum* spp. (Canada Western Amber Durum)	Western Canada
KAS5473	*A. infectoria*	3	*Triticum* spp. (Canada Western Red Winter)	Western Canada
KAS5472	*A. infectoria*	3	*Triticum* spp. (Canada Western Red Winter)	Western Canada
KAS5471	ND	1	*Triticum* spp. (Canada Western Red Winter)	Western Canada
KAS5470	*A. infectoria*	3	*Triticum* spp. (Canada Western Red Winter)	Western Canada
KAS5469	ND	3	*Triticum* spp. (Canada Western Red Winter)	Western Canada

KAS5468	*A. alternata*	4	*Triticum* spp. (Canada Western Red Winter)	Western Canada
KAS5467	ND	3	*Triticum* spp. (Canada Western Red Winter)	Western Canada
KAS5465	*A. alternata*	1	*Triticum* spp. (Canada Western Prairie Spring Red)	Western Canada
KAS5464	*A. alternata*	1	*Triticum* spp. (Canada Western Prairie Spring Red)	Western Canada
KAS5463	*A. infectoria*	3	*Triticum* spp. (Canada Western Prairie Spring Red)	Western Canada
KAS5462	*A. alternata*	1	*Hordeum vulgare* L. (Canada Western)	Western Canada
KAS5461	*A. alternata*	4	*Hordeum vulgare* L. (Canada Western)	Western Canada
KAS5460	*A. alternata*	1	*Hordeum vulgare* L. (Canada Western)	Western Canada
KAS5459	*A. alternata*	1	*Hordeum vulgare* L. (Canada Western)	Western Canada
KAS5458	*A. alternata*	1	*Hordeum vulgare* L. (Canada Western)	Western Canada
KAS5457	*A. alternata*	1	*Hordeum vulgare* L. (Canada Western)	Western Canada
KAS5456	*A. alternata*	2	*Hordeum vulgare* L. (Canada Western)	Western Canada
KAS5454	ND	3	*Triticum* spp. (Canada Western Hard Red Spring)	Western Canada
KAS5453	*A. alternata*	1	*Avena sativa* (Canada Western)	Edmonton, Alberta
KAS5452	*A. infectoria*	3	*Avena sativa* (Canada Western)	Edmonton, Alberta
KAS5450	*A. alternata*	4	*Triticum* spp. (Canada Western Amber Durum)	Corrine, Saskatchewan
KAS5449	*A. infectoria*	3	*Triticum* spp. (Canada Western Amber Durum)	Corrine, Saskatchewan
KAS5448	*A. alternata*	4	*Triticum* spp. (Canada Western Amber Durum)	Corrine, Saskatchewan
KAS5447	ND	3	*Triticum* spp. (Canada Western Red Winter)	Killam, Alberta
KAS5446	*A. infectoria*	3	*Triticum* spp. (Canada Western Red Winter)	Dunmore, Alberta

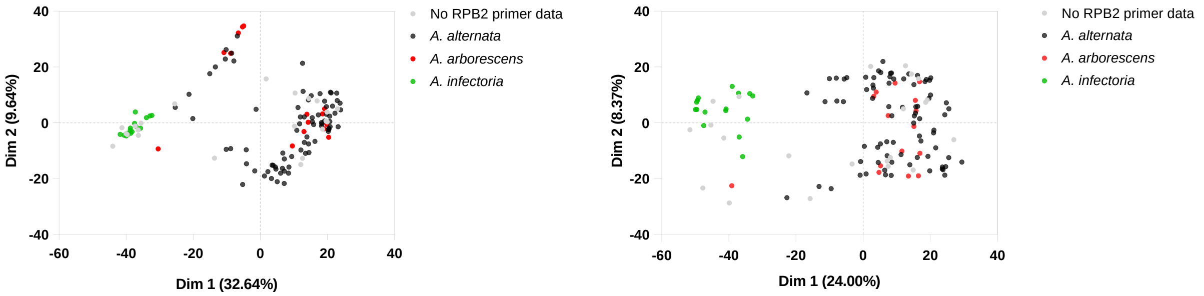

Figure S1- PCA plots in negative and positive ionization modes coloured by available RPB2 primer results

Table S2- Peak list generation conditions in xcms in R

Condition	Setting
Prefilter=c(k,I)	k = 3, I = 5000
ppm	2
snthresh	5
peakwidth=c(min,max)	min = 5, max = 20
noise	100000
retcor method	orbiwarp
bw	5
minfrac	0.25
mzwid	0.015
